# Tubular β-catenin alleviates mitochondrial dysfunction and cell death in acute kidney injury

**DOI:** 10.1038/s41419-022-05395-3

**Published:** 2022-12-20

**Authors:** Hongyu Li, Joseph C. K. Leung, Wai Han Yiu, Loretta Y. Y. Chan, Bin Li, Sarah W. Y. Lok, Rui Xue, Yixin Zou, Kar Neng Lai, Sydney C. W. Tang

**Affiliations:** Division of Nephrology, Department of Medicine, The University of Hong Kong, Hong Kong, China

**Keywords:** Experimental models of disease, Acute kidney injury

## Abstract

Mitochondria take part in a network of intracellular processes that regulate homeostasis. Defects in mitochondrial function are key pathophysiological changes during AKI. Although Wnt/β-catenin signaling mediates mitochondrial dysfunction in chronic kidney fibrosis, little is known of the influence of β-catenin on mitochondrial function in AKI. To decipher this interaction, we generated an inducible mouse model of tubule-specific β-catenin overexpression (TubCat), and a model of tubule-specific β-catenin depletion (TubcatKO), and induced septic AKI in these mice with lipopolysaccharide (LPS) and aseptic AKI with bilateral ischemia-reperfusion. In both AKI models, tubular β-catenin stabilization in TubCat animals significantly reduced BUN/serum creatinine, tubular damage (NGAL-positive tubules), apoptosis (TUNEL-positive cells) and necroptosis (phosphorylation of MLKL and RIP3) through activating AKT phosphorylation and p53 suppression; enhanced mitochondrial biogenesis (increased PGC-1α and NRF1) and restored mitochondrial mass (increased TIM23) to re-establish mitochondrial homeostasis (increased fusion markers OPA1, MFN2, and decreased fission protein DRP1) through the FOXO3/PGC-1α signaling cascade. Conversely, kidney function loss and histological damage, tubular cell death, and mitochondrial dysfunction were all aggravated in TubCatKO mice. Mechanistically, β-catenin transfection maintained mitochondrial mass and activated PGC-1α via FOXO3 in LPS-exposed HK-2 cells. Collectively, these findings provide evidence that tubular β-catenin mitigates cell death and restores mitochondrial homeostasis in AKI through the common mechanisms associated with activation of AKT/p53 and FOXO3/PGC-1α signaling pathways.

## Introduction

Kidney tubular dysfunction and cell death contribute heavily to the pathophysiology of acute kidney injury (AKI). Catenin beta-1, also known as β-catenin, is a crucial signal transducer that regulates cell adhesion and maintains kidney cell homeostasis. Its activity remains largely quiescent in the adult kidney where in the absence of Wnt stimulation, β-catenin is degraded by ubiquitination-dependent proteolysis in the proteosome after its phosphorylation through the assembly of the destruction complex consisting of tumor suppressor proteins adenomatous polyposis coli (APC), dishevelled (DVL) protein, and two serine-threonine kinases, casein kinase 1 (CK1) and glycogen synthase kinase 3β (GSK3β). β-catenin becomes reactivated in pathological states such as glomerulonephritis, diabetic kidney disease and lupus nephritis [1–4]. In the canonical activation pathway, Wnt signaling inhibits β-catenin phosphorylation leading to its cytosolic accumulation and subsequent translocation into the nucleus where it interacts with the T cell factor/lymphoid enhancer binding factor (TCF/LEF) family of transcription factors to activate Wnt/β-catenin-responsive genes [5]. Non-canonical β-catenin signaling via binding TCF/LEF independent transcription factors, including androgen receptor [6], hypoxia-inducible factor-1α (HIF-1α) [7], and the class O of forkhead box (FOXO) family [8], and their downstream targets is increasingly observed. Studies have suggested a protective role of β-catenin in AKI as it regulates tubular epithelial cell dedifferentiation to promote tubule restoration after acute injury [9]. Activation of β-catenin protects tubular cells from apoptosis by upregulating anti-apoptotic factors and suppressing pro-apoptotic factors [10–12]. Inhibiting β-catenin aggravates kidney injury by enhancing both intracellular and mitochondrial levels of reactive oxygen species (ROS) in cisplatin-induced AKI rats [13]. In chronic models of kidney disease, β-catenin signaling blockade alleviates kidney injury and fibrosis [14, 15]. Paradoxically, β-catenin interaction with non-canonical transcription factors exerts protection in chronic kidney disease (CKD) [16, 17].

Mitochondrial dysfunction orchestrates key pathophysiological changes during AKI in which mitochondrial biogenesis, mitochondrial dynamics and mitophagy were altered [18–20]. Mitochondrial biogenesis maintains mitochondrial homeostasis through regulating mitochondrial DNA (mtDNA) replication, nuclear encoded mitochondrial protein importation and mitochondrial renewal. Peroxisome proliferator-activated receptor gamma co-activator 1 alpha (PGC-1α) is the master regulator of mitochondrial biogenesis [21–23]. Clinically, PGC-1α expression was suppressed during AKI [24]. Experimentally, proximal tubule-specific PGC-1α knockout mice were more susceptible to septic AKI [18]. Furthermore, β-catenin inhibits mitochondrial biogenesis in age-related renal fibrosis and AKI-to-CKD transition [15, 16]. However, no studies to date have shown how differential tubular expression of β-catenin regulates mitochondrial function in the development of AKI.

In this study, we adopt a strategy of loss- and gain-of-β-catenin function by generating two strains of transgenic mice with tubule-specific β-catenin deletion or stabilization (through exon 3 deletion) to delineate the role of tubular β-catenin and the mechanistic pathways associated with mitochondrial biogenesis and cell death in murine septic and aseptic AKI.

## Materials and methods

### Mice and genotyping

All animal experiments were approved by the Committee on the Use of Live Animals in Teaching and Research (Centre for Comparative Medicine Research, The University of Hong Kong) and followed strictly the National Institute of Health Guide for the Care and Use of Laboratory Animals. A tubular cell-specific β-catenin stabilization mouse (TubCat mice, *KspCre*^*ERT2*^*; Catnb*^*lox(ex3)/wt)*^) was previously generated in our laboratory [25]. Their littermates without the *KspCre*^*ERT2*^ allele served as control (CTL mice, *Catnb*^*lox(ex3)/wt*^). The homozygous β-catenin-floxed (exon 2-exon 6) mouse was generated as previously described [26]. By mating β-catenin-floxed mice with *KspCre*^*ERT2*^ mice, renal tubular epithelial cell-specific conditional knockout mice (TubCatKO mice, *KspCre*^*ERT2*^*; Catnb*^*lox/lox*^) were obtained. Their littermates without the *KspCre*^*ERT2*^ allele served as the corresponding control (KO CTL mice, *Catnb*^*lox/lox*^). Mouse ear punch samples were used for genotyping by KAPA2G Fast HS Genotyping Mix kit (Sigma-Aldrich, St. Louis, MO) according to the manufacturer’s instructions. The genotyping primer sequences were listed in Supplemental Table S1. Male mice at 7 weeks of age received tamoxifen (1 mg/g body weight, Sigma-Aldrich) dissolved in corn oil containing 10 % ethanol for 5 consecutive days via intraperitoneal injection to induce *Cre* recombination.

### Mouse models of AKI

(1) Ischemia-reperfusion injury (IRI)-induced AKI was established as previously described with modifications [12]. After anesthesia, mice received midline incision and bilateral renal pedicles were clamped for 28 min. During the ischemia period, mice were kept at 37 °C. After removal of the clamps, mice underwent 24 h of reperfusion before sacrifice. (2) Lipopolysaccharide (LPS)-induced AKI was established as previously described [27]. Mice received a single injection of LPS (20 mg/kg body weight, Sigma-Aldrich) dissolved in saline and were sacrificed after 24 h. TubCat mice and TubCatKO mice were randomly assigned to the control (sham or vehicle) or AKI group (IRI or LPS), *n* = 5 per group. In both models, blood and kidney tissues were collected for subsequent analyses. Investigators were blinded when assessing the outcomes (kidney function, histology, immunostaining, and Western blot).

### Kidney function

Serum creatinine (sCr) and blood urea nitrogen (BUN) were determined by Stanbio Direct Creatinine LiquiColor Procedure Kit (Stanbio Laboratory, Boerne, TX) and QuantiChrom^TM^ Urea Assay Kit (BioAssay Systems, Hayward, CA) according to the manufacturer’s instructions.

### Histology and immunohistochemical staining

Paraffin-embedded mouse kidney sections (4-μm thickness) were prepared for Periodic acid Schiff (PAS) staining (Abcam, Cambridge, UK). Percentages of intact, moderately, and severely damaged renal tubules were quantified on ten non-overlapping high-power cortical fields of PAS-stained sections by three blinded examiners as previously described [28] using the following criteria: Intact tubules are identified by normal morphology. Complete loss of cell nuclei indicated severely damaged tubules. Tubules with dilatation, cast formation, brush border loss were counted as moderately damaged tubules. Immunohistochemical staining of neutrophil gelatinase-associated lipocalin (NGAL) (ab70287, Abcam) was performed [25]. For double immunostaining of β-catenin and NGAL, sections were incubated with antibodies against rabbit anti-mouse β-catenin (ΕP-35, Becton, Dickinson and Company, Franklin Lakes, NJ) plus rat anti-mouse NGAL at 4 °C overnight, followed by incubation with anti-rat horseradish peroxidase (HRP) plus rabbit alkaline phosphatase (AP) polymer for 30 min. Permanent Red and Emerald chromogens were used for color development (red color from AP and blue color from HRP).

### Transmission electron microscopy

Mouse kidney samples were freshly collected in 1 mm^3^ blocks and fixed in 2.5% glutaraldehyde in cacodylate buffer (0.1 M sodium cacodylate-HCl buffer pH 7.4) at 4 °C overnight and cut into 100 nm ultra-thin sections before staining with 2% aqueous uranyl acetate (performed by the Electron Microscope Unit, The University of Hong Kong). Digital images were obtained with a Philips CM 100 transmission electron microscope (Philips Electro Optics, Cambridge, UK) at an operating voltage of 100 kV.

### TUNEL staining

Τerminal deoxynucleotidyl transferase dUTP nick end labeling (TUNEL) assay (ApopTag® Peroxidase In Situ Apoptosis Detection Kit; EMD Millipore, Billerica, MA) was used to detect apoptosis following the manufacturer’s procedure.

### Immunofluorescence staining

Paraffin-embedded mouse kidney sections (4-μm thickness) and paraformaldehyde-fixed HK-2 cells were used for immunofluorescence staining, and reagents were obtained from Abcam. Briefly, kidney paraffin-sections were dewaxed and rehydrated. Citrate buffer (pH 6.0) was used for antigen retrieval. After treating with hydrogen peroxide blocking reagent for 10 min, slides were blocked with mouse-on-mouse blocking solution followed by 5% goat serum for 30 min. Sections were incubated with monoclonal anti-mouse β-catenin (ΕP-35, Becton, Dickinson and Company) plus rabbit anti-mouse PGC-1α (ab54481, Abcam) overnight, followed by incubation with AlexaFluor 488 goat anti-rabbit plus AlexaFluor 594 goat anti-mouse secondary antibodies (Thermo Fisher Scientific, Grand Island, NY). For immunofluorescent staining on tubular epithelial cells, HK-2 cells were cultured on LabTek chamberslides (Thermo Fisher Scientific). After pretreatment with or without LPS treatment for 30 minutes, cells were fixed by 4% paraformaldehyde for 15 min, following by 2% BSA blocking for 30 min. Subsequently, cells were incubated with anti-mouse β-catenin monoclonal antibody (610154, Becton, Dickinson and Company) plus rabbit anti-mouse FOXO3 antibody (ab109629, Abcam) overnight, followed by incubation with AlexaFluor 488 goat anti-mouse plus AlexaFluor 594 goat anti-rabbit secondary antibodies (Thermo Fisher Scientific) for 1 h. Both kidney sections and fixed cells were co-stained with DAPI for 10 min, then mounted with antifade mounting medium (Vector Laboratories, Burlingame, CA). Fluorescence images were visualized and captured under a fluorescence microscope (Olympus, Tokyo, Japan).

### Cytoplasmic/nuclear fraction

Nuclear fractions were isolated from kidney cortical tissue and HK-2 cells by NE-PER kit (Pierce, Rockford, IL). Protein concentrations of nuclear fractions were determined by BCA Protein Assay Kit (Pierce).

### Cell culture and treatment

Human proximal tubular epithelial cell line, HK-2 cells, was purchased from American Type Culture Collection (ATCC, Manassas, VA), cultured in DMEM/F12, GlutaMAX^TM^ supplement medium (Gibco, Grand Island, NY) containing 10% fetal bovine serum (Gibco) and 1% penicillin–streptomycin (Gibco) in an incubator at 37 °C with 5% CO_2_. HK-2 cells were treated with 100 ng/µl LPS for various time points. RNA and protein were collected for subsequent experiments. To stabilize β-catenin, an empty vector pcDNA3-2AB Flag and β-catenin-4A mutant (S33/S37/T41/S45) plasmid (Addgene plasmid #24024, Cambridge, MA) were transiently transfected into HK-2 cells with Lipofectamine 2000 (Invitrogen, Carlsbad, CA) according to manufacturer’s instructions. In all experiments, transfected cells were treated with 100 ng/µl LPS.

### MitoTracker Red staining

HK-2 cells were seeded onto Chamber Slide (Thermo Fisher Scientific) at 2 × 10^4^ cells per well. Cells were transfected with empty vector or β-catenin-4A mutant plasmid followed by LPS treatment. MitoTracker^®^ Red CMXRos (300 nM, Invitrogen) was added into culture media and incubated at 37 °C for 30 min. Cells were then fixed by cold methanol and co-stained with DAPI. MitoTracker staining was visualized by confocal microscopy (LSM980 with Airyscan, Carl Zeiss, Germany). Intensity of MitoTracker was quantified by Image J software on 10 random fields at 400 × magnification.

### Co-immunoprecipitation assay

The interaction of β-catenin and FOXO3 was determined by co-immunoprecipitation assay (Classic Magnetic co-IP kit, Pierce) as previously described [29]. HK-2 cells were transfected with β-catenin-4A mutant plasmid transfection and incubated with or without LPS for 30 min. Nuclear fractions were immunoprecipitated with anti-β-catenin antibody (610154, Becton, Dickinson and Company) at 4 °C overnight. Magnetic protein A/G beads were added and incubated at RT for 2 h. Input and immunoprecipitates were immunoblotted with the following antibodies: β-catenin (8480, Cell Signaling Technology, Danvers, MA), FOXO3 (ab109629, Abcam) and GAPDH (ABS16, EMD Millipore).

### Chromatin immunoprecipitation assay

Chromatin immunoprecipitation (ChIP) assay was performed using a Magnetic ChIP kit (26157, Thermo Fisher Scientific). Briefly, HK-2 cells were transfected with vector plasmid or β-catenin-4A mutant plasmid. 24 h after transfection, protein-DNA complex was cross-linked with 1% formaldehyde for 10 min at RT. Chromatin fragmentation was achieved by micrococcal nuclease digestion and ultrasound sonication. 10% sonicated samples were saved as 10% input. The remaining samples were immunoprecipitated with either FOXO3 ChIP-grade antibody (720128, Thermo Fisher Scientific) or normal rabbit IgG (2729, Cell Signaling Technology) at 4 °C overnight. ChIP-grade protein A/G magnetic beads were added and incubated for 2 h at 4 °C. After DNA recovery and purification, FOXO3-PGC-1α promoter binding within precipitated chromatin was analyzed by qPCR. The PGC-1α promoter region primers were designed by Primer-BLAST and listed in Supplementary Table S1. Fold enrichment for PGC-1α is calculated as the ratio of FOXO3 ChIP to IgG ChIP. ChIP assays were repeated three times.

### RNA extraction and real-time RT-PCR

Kidney cortical RNA was extracted by using NucleoSpin® kit (Macherey-Nagel, Düren, Germany). High-capacity cDNA Reverse Transcription Kit (Applied Biosystems, Foster City, CA) was used for RNA reverse transcription to cDNA. Real-time PCR was performed using specific primers (Supplemental Table S1) via StepOne software v2.3 (Applied Biosystems).

### Western blot

Renal cortical protein lysate was isolated by NucleoSpin® kit (Macherey-Nagel). Total protein concentration was quantified using BCA Protein Assay Kit (Pierce). Western blot was used to determine protein expression [12]. Primary antibodies: p-MLKL (ab196436), PGC-1α (ab54481, on mouse tissue), NRF1 (ab175932), OPA1 (ab157457), MFN2 (ab56889), DRP1 (ab184247), FOXO3 (ab12162 on mouse tissue and ab109629 on HK-2 cell lysates) were obtained from Abcam; p-RIP3 (91702), p-AKT (4060), AKT (9272), p-p53 (9284) were purchased from Cell Signaling Technology; β-ACTIN (Ms1295) were from Thermo Fisher Scientific; TIM23 (sc-514463), HDAC1 (sc-81598) were from Santa Cruz (Santa Cruz, CA) and PGC-1α (A12348 on HK-2 cell lysates) from ABclonal (Cambridge, MA).

### Statistical analysis

Statistical analysis was performed using GraphPad Prism 9 (GraphPad Software, San Diego, CA). All data were expressed as mean ± SEM. Comparisons among groups were made by one-way ANOVA, followed by the Turkey test. *p* < 0.05 was considered statistically significant.

## Results

### Tubular β-catenin protects against kidney injury after IRI

**Fig. 1:**
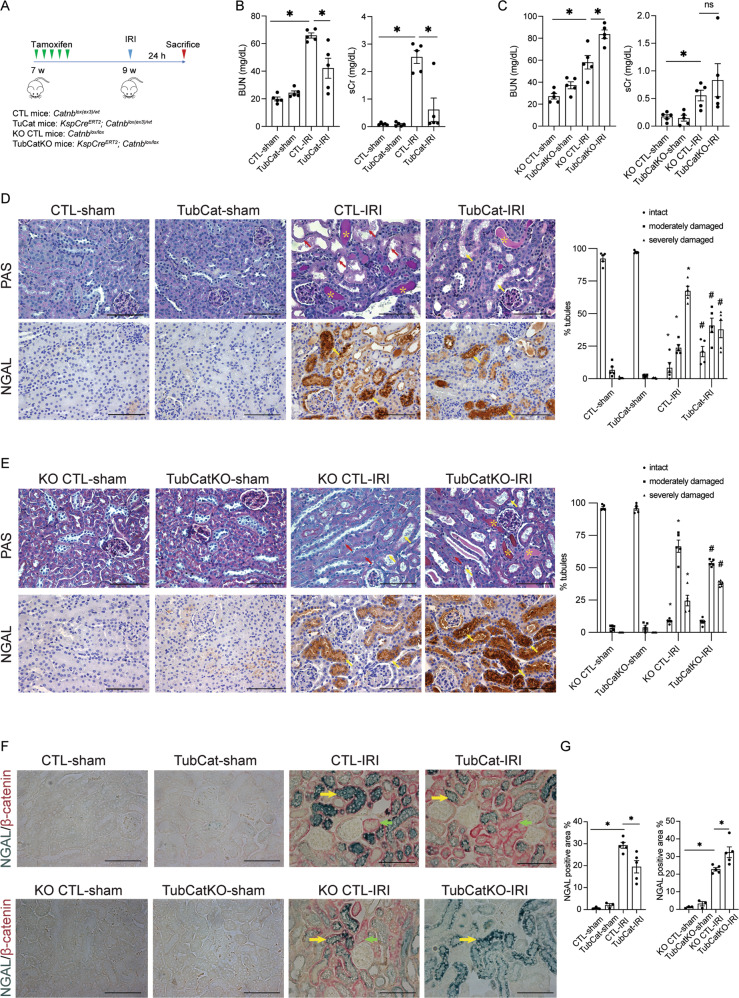
Tubular β-catenin protects against acute kidney injury after IRI. **A** Experimental design of IRI-induced AKI. **B** BUN levels and sCr levels in TubCat mice after IRI. *p < 0.05; *n* =5 in each group. **C** BUN levels and sCr levels in TubCatKO mice after IRI. BUN, blood urea nitrogen; sCr, serum creatinine; *p < 0.05; *n* = 5 in each group. **D**, **E** Representative micrographs of PAS staining and NGAL immunohistochemical staining at 24 h after IRI in TubCat mice (**D**) or TubCatKO mice (**E**), and their quantitative data on histologic injury presented as percentage of intact, moderately, and severely damaged tubules, respectively, after IRI. Dilated or necrotic tubules are indicated by yellow or red arrows; tubular cast formation is marked with yellow asterisks; yellow arrows denote NGAL-positive areas. *p < 0.05 vs. CTL-sham or KO CTL-sham; ^#^*p* < 0.05 vs. CTL-IRI or KO CTL-IRI; *n* = 5 in each group. **F** Double-immunohistochemical staining of NGAL (blue, as indicated by yellow arrows) and β-catenin (red, green arrows) in TubCat-IRI mice or TubCatKO-IRI mice. Scale bar = 100 μm. **G** Quantitative data on tubular injury presented as percentage NGAL-positive area per HPF in TubCat-IRI mice or TubCatKO-IRI mice, respectively. *p < 0.05; *n* = 3 in sham groups and *n* = 5 in IRI groups.

TubCat (tubule-specific β-catenin stabilized) and TubCatKO (tubule-specific β-catenin deleted) mice and their respective control animals (CTL and KO CTL) were subjected to bilateral renal ischemia followed by 24 h reperfusion (Fig. 1A). AKI was evident from ≥ two-fold increase in BUN and sCr after IRI (Fig. 1B, C), which was reduced in TubCat mice (30% reduction in BUN and 70% reduction in sCr) but elevated in TubCatKO mice (40% increase in BUN) vs. their respective controls. As shown in Fig. 1D, E, kidney tubular damage (cast formation, dilatation, and tubule necrosis) after IRI from both mouse strains was evident from a loss of intact tubules (8.54% in CTL-IRI vs. 92.39% in CTL-sham; 8.72% in KO CTL-IRI vs. 96.44% in KO CTL-sham) and increased percentage of both moderately damaged tubules (23.89% in CTL-IRI vs. 6.92% in CTL-sham; 66.65% in KO CTL-IRI vs. 3.56% in KO CTL-sham) and severely damaged tubules (67.57% in CTL-IRI vs. 0.69% in CTL-sham; 24.63% in KO CTL-IRI vs. 0% in KO CTL-sham). Tubular injury was mitigated in TubCat-IRI mice with significant increase in intact tubule (20.86%) and decrease in severely damaged tubule (38.11%) compared to CTL-IRI mice. However, there was also an increase in moderately damaged tubule (41.03%) in TubCat-IRI mice. On the other hand, TubCatKO-IRI had significantly higher number of severely damaged tubule (37.91%) and lower number of moderately damaged tubule (53.76%) when compared to KO CTL-IRI mice, while the percentage of intact tubule (8.33%) reminded unchanged. Detailed percentage of damaged tubules was showed in Suppl Table S2. Tubular NGAL expression, a marker of acute tubular injury, was significantly upregulated after IRI. NGAL-positive area was reduced in TubCat mice but increased in TubCatKO mice vs. their corresponding controls (Fig. 1G). Co-staining of NGAL and β-catenin showed that β-catenin-positive tubules have less NGAL expression (Fig. 1F), implying that β-catenin expression might protect tubular injury in IRI-induced AKI.

### Tubular β-catenin protects against LPS-induced AKI

**Fig. 2:**
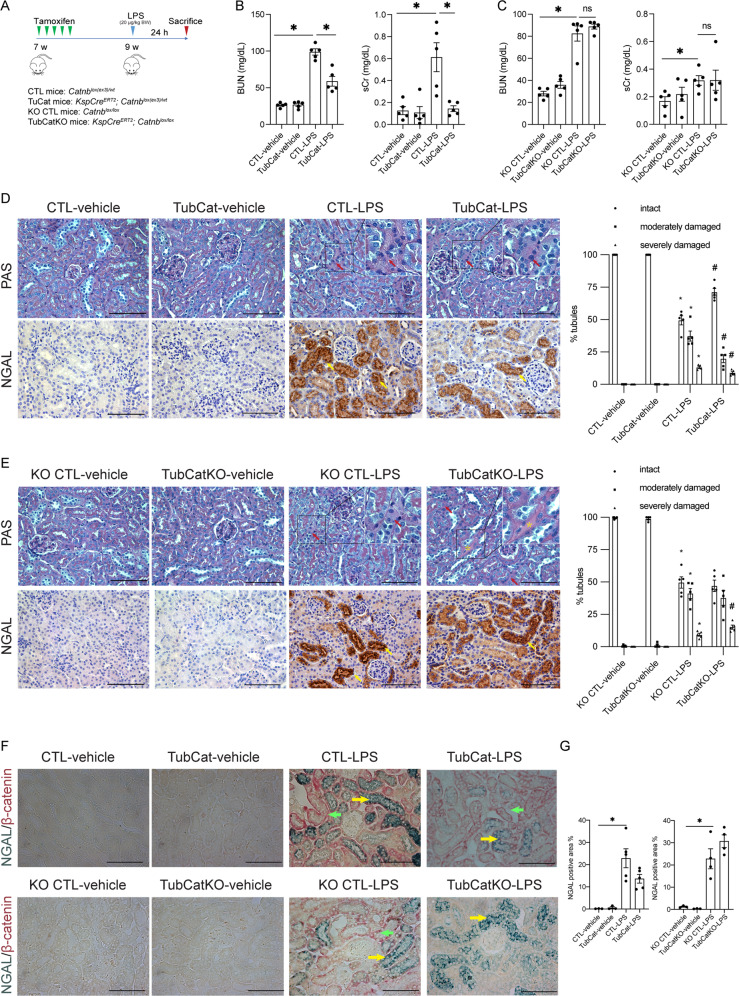
Tubular β-catenin protects against LPS-induced AKI. **A** Experimental design of LPS-induced AKI. **B** BUN levels and sCr levels in TubCat mice at 24 h after LPS injection. *p < 0.05; *n* = 5 in each group. **C** BUN levels and sCr levels in TubCatKO mice at 24 h after LPS injection. *p < 0.05; *n* = 5 in each group. **D**, **E** Representative micrographs of PAS staining and NGAL immunohistochemical staining after LPS injection in TubCat mice (**D**) or TubCatKO mice (**E**), and their quantification of percentage injured tubules after LPS injection. Necrotic tubules are indicated by red arrows; tubular cast formation is marked with yellow asterisks; yellow arrows denote NGAL-positive areas. *p < 0.05 vs. CTL-vehicle or KO CTL-vehicle; #*p* < 0.05 vs. CTL-LPS or KO CTL-LPS; *n* = 5 in each group. **F** Double-immunohistochemical staining of NGAL (blue, as indicated by yellow arrows) and β-catenin (red, green arrows) in TubCat-LPS mice or TubCatKO-LPS mice. Scale bar = 100 μm. **G** Quantitative data on tubular injury presented as percentage NGAL-positive area per HPF in TubCat-LPS mice or TubCatKO-LPS mice, respectively. *p < 0.05; *n* = 3 in vehicle groups and *n* = 5 in LPS groups.

In the septic AKI model (Fig. 2A), increase in BUN and sCr levels in CTL-LPS (three-fold) and KO CTL-LPS (>two-fold) mice were significant after LPS injection vs. their respective controls, which were reduced in TubCat mice (50% reduction) but unchanged in TubCatKO mice (Fig. 2B, C). Histologically, LPS-treated mice had reduced number of intact tubules (49.47% in CTL-LPS vs. 100% in CTL-vehicle; 49.62% in KO CTL-LPS vs. 99.50% in KO CTL-vehicle), increased proportion of moderately damage tubules (37.31% in CTL-LPS vs. 0% in CTL-vehicle; 41.16% in KO CTL-LPS vs. 0.50% in KO CTL-vehicle) and severely damaged tubules (13.22% in CTL-LPS vs. 0% in CTL-vehicle; 9.22% in KO CTL-LPS vs. 0% in KO CTL-vehicle) compared to their respective controls. Tubular injury in TubCat-LPS mice was ameliorated with an increased percentage of intact tubule (71.26%) and reduction in moderately (19.84%) and severely (8.90%) damaged tubules compared to CTL-LPS mice (Fig. 2D and Suppl Table S2). TubCatKO-LPS mice had higher number of severely damaged tubule (15.12%) while the percentage of intact tubule (47.08%) and moderately damaged tubules (37.80%) showed no statistical difference compared to KO CTL-LPS (Fig. 2E and Suppl Table S2). NGAL overexpression in the tubules were ameliorated in TubCat mice but exacerbated in TubCatKO mice (Fig. 2G). NGAL/β-catenin co-staining showed that reduced NGAL overexpression was observed mainly in β-catenin-positive tubules (Fig. 2F).

### β-catenin reduces kidney cell death in AKI via AKT/p53 signaling

**Fig. 3:**
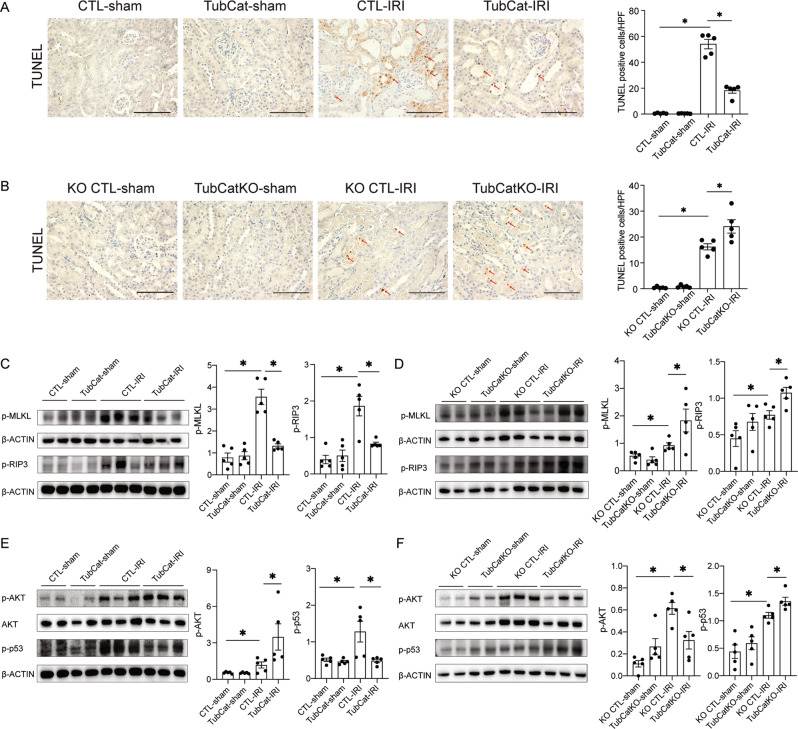
β-catenin reduces kidney cell death in IRI-induced AKI via AKT/p53 signaling. **A**, **B** Apoptotic cell death: Representative TUNEL staining and quantification of apoptotic cells per HPF in kidney cortex from TubCat-IRI mice (**A**) and TubCatKO-IRI mice (**B**). Red arrows, apoptotic cells; Scale bar = 100 μm. HPF high-power field. *p < 0.05; *n* = 5 in each group. **C**, **D** Necroptotic cell death expressed as protein expression of phosphorylated necroptotic executive proteins, MLKL and RIP3 (p-MLKL and p-RIP3) by Western blots of kidney cortical lysates and quantitative analyses in TubCat-IRI mice (**C**) and TubCatKO-IRI mice (**D**). *p < 0.05; *n* = 5 in each group. **E**, **F** Representative Western blots of p-AKT and p-p53 and their quantification in TubCat-IRI mice (**E**) and in TubCatKO-IRI mice (**F**). *p < 0.05; *n* = 5 in each group.

IRI-induced tubule cell apoptosis was reduced in TubCat mice but increased in TubCatKO animals (Fig. 3A, B). IRI also induced necroptotic cell death as shown by an increase in phosphorylation of mixed lineage kinase domain like pseudokinase protein (MLKL) and receptor-interacting protein kinase 3 (RIP3), which was attenuated in TubCat mice but increased in TubCatKO mice (Fig. 3C, D). The downstream signaling molecules involved in β-catenin regulation of IRI-induced cell death, phosphorylated AKT and p53, were examined by Western blotting (Fig. 3E, F). Compared with their corresponding controls, increased p-AKT accompanied with reduced p-p53 were shown in TubCat mice. In TubCatKO mice, reduced p-AKT and increased p-p53 expression were observed.

**Fig. 4:**
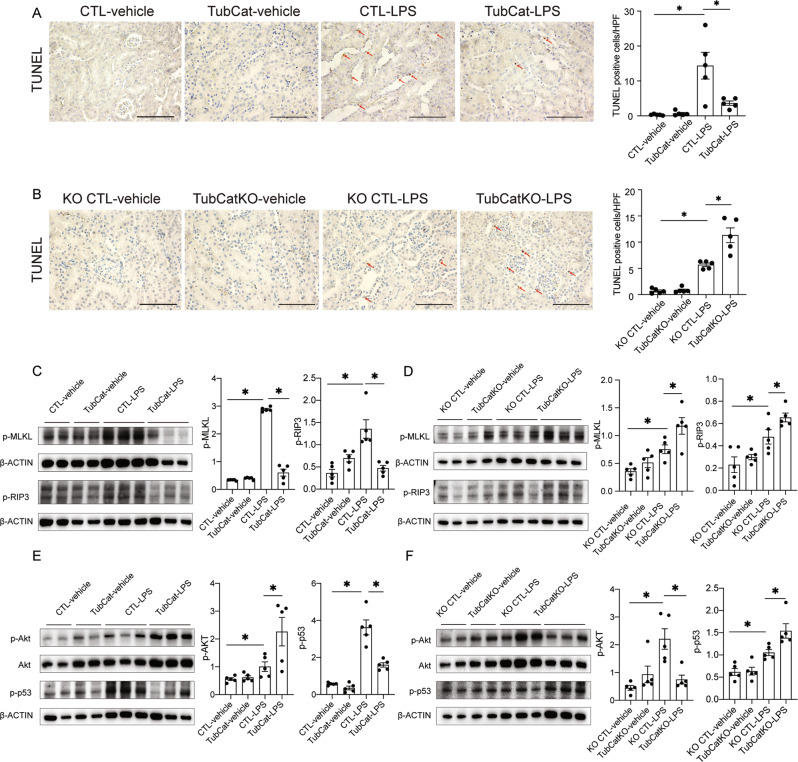
β-catenin reduces kidney cell death in LPS-induced AKI via AKT/p53 signaling. **A**, **B** Apoptotic cell death: representative TUNEL staining and quantification of apoptotic cells per HPF in kidney cortex from TubCat-LPS mice (**A**) and TubCatKO-LPS mice (**B**). Red arrows, apoptotic cells; Scale bar = 100 μm. *p < 0.05; *n* = 5 in each group. **C**, **D** Necroptotic cell death expressed as protein expression of p-MLKL and p-RIP3 by western blots of kidney cortex and quantitative analyses in TubCat-LPS mice (**C**) and TubCatKO-LPS mice (**D**). *p < 0.05; *n* = 5 in each group. **E**, **F** Representative Western blots of p-AKT and p-p53 and their quantification in TubCat-LPS mice (**E**) and in TubCatKO-LPS mice (**F**). *p < 0.05; *n* = 5 in each group.

The protective role of β-catenin against tubular apoptosis and necroptosis was confirmed in septic AKI induced by LPS in which apoptosis and overexpression of p-MLKL and p-RIP3 were ameliorated in TubCat mice but exacerbated in TubCatKO mice (Fig. 4A–D). Changes in p-AKT and p-p53 expression were similar to the IRI model (Fig. 4E, F).

### β-catenin restores tubular mitochondrial homeostasis in AKI

**Fig. 5:**
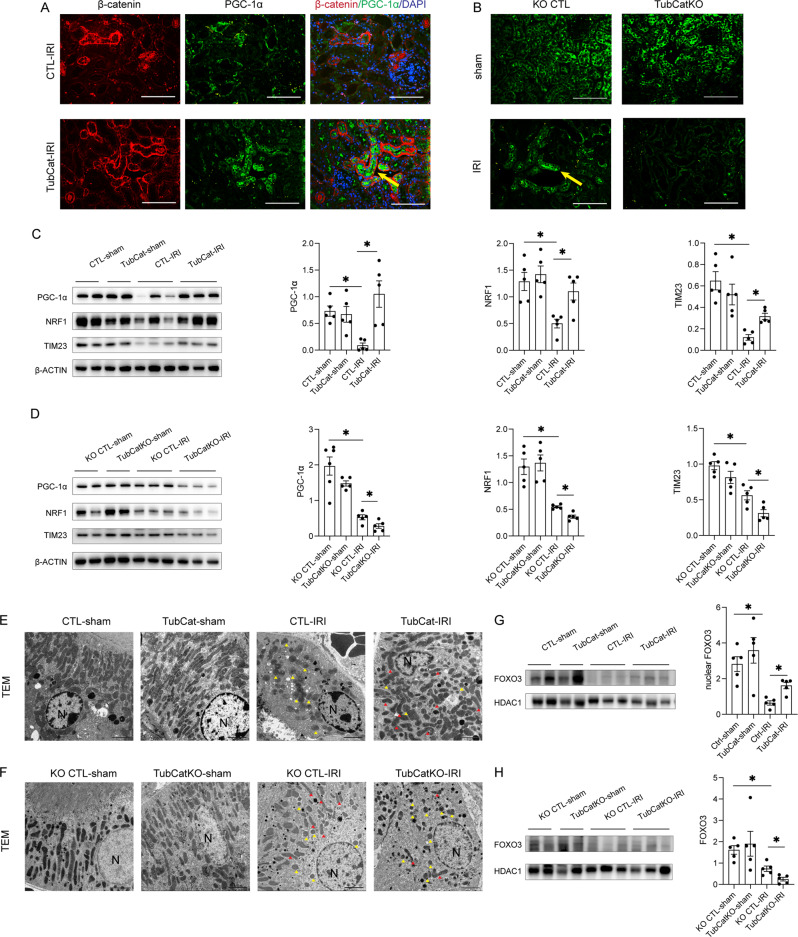
β-catenin restores tubular mitochondrial biogenesis in IRI-induced AKI. **A** Double-immunofluorescent staining of β-catenin (red) and PGC-1α (green) in kidney section from TubCat-IRI mice. DAPI (blue) reveals nuclear staining, and yellow arrow denotes co-localization of β-catenin and PGC-1α. Scale bar = 100 μm. **B** Immunofluorescent staining of PGC-1α (green) in kidney cortex from TubCatKO-IRI mice. Yellow arrow indicates PGC-1α-positive tubule. Scale bar = 100 μm. **C** Representative western blots and their quantification of mitochondrial marker TIM23 and mitochondrial biogenesis regulators (PGC-1α, NRF1) in kidney cortical lysates in TubCat-IRI mice. *p < 0.05; *n* = 5 in each group. **D** Representative western blots and their quantification of TIM23, PGC-1α and NRF1 in kidney cortical lysates in TubCatKO-IRI mice. *p < 0.05; *n* = 5 in each group. **E**, **F** Representative transmission electronic microscopy images in TubCat-IRI mice (**E**) and TubCatKO-IRI mice (**F**). Yellow arrowheads are swollen mitochondria, red arrowheads refer to intact mitochondria. N, nucleus. Scale bar = 2 μm. **G**, **H** representative Western blot of FOXO3 in kidney nuclear fraction of TubCat-IRI and TubCatKO-IRI group, HDAC1 was used as nuclear protein loading control. *p < 0.05. *n* = 5 in each group.

Double-immunofluorescence staining showed that PGC-1α, the master regulator of mitochondrial biogenesis, was upregulated in TubCat-IRI kidneys and co-stained with β-catenin-positive tubules (Fig. 5A), whereas in TubCatKO-IRI kidneys, PGC-1α was downregulated vs. KO CTL-IRI mice (Fig. 5B). PGC-1α protein expression showed the same results (Fig. 5C, D). Reduced expression of PGC-1α and its downstream transcription factor nuclear respiratory factor 1 (NRF1) after IRI was restored in TubCat-IRI kidneys (Fig. 5C). In TubCatKO mice, expression of PGC-1α and NRF1 was further downregulated vs. the KO CTL-IRI group (Fig. 5D). Mitochondrial mass was significantly decreased after IRI as reflected by reduced expression of the mitochondrial structure protein TIM23, which was restored in TubCat-IRI kidneys but further decreased in TubCatKO mice vs. their controls (Fig. 5C, D).

Transmission EM (Fig. 5E, F) showed reduced mitochondrial number as well as damaged mitochondrial structure in IRI kidneys. In TubCat-IRI kidneys, mitochondria were less swollen and there were more intact mitochondria. On the contrary, TubCatKO-IRI kidneys had less mitochondria, and displayed more swollen mitochondria than intact mitochondria, compared to KO CTL-IRI kidneys. Consistent with the EM findings, ATP levels and mtDNA copy number were significantly reduced in CTL-IRI/LPS and KO CTL-IRI/LPS mice (Suppl Fig. S1) compared to corresponding sham and vehicle controls. ATP production and mtDNA duplication were rescued in TubCat-IRI group but there was no further depletion in TubCatKO-IRI group.

Forkhead box O3 (FOXO3), an upstream regulator of PGC-1α, was significantly downregulated in cortical kidney nuclear fraction after IRI (Fig. 5G, H). It was restored in TubCat-IRI kidneys but further suppressed in the TubCatKO group.

**Fig. 6:**
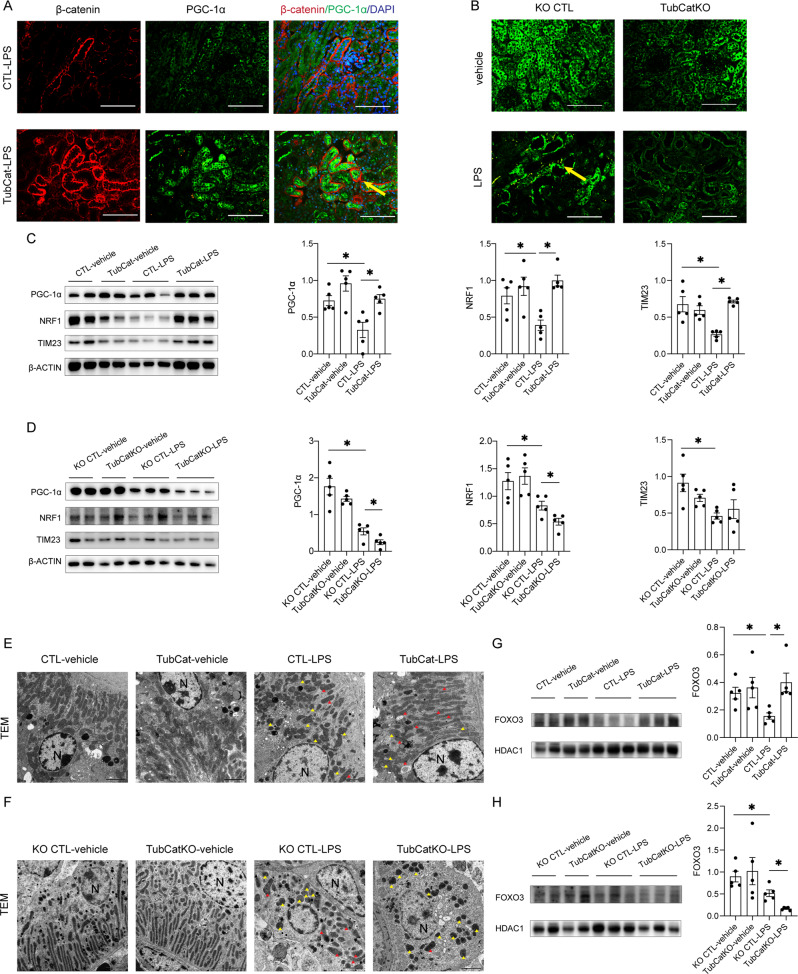
β-catenin restores tubular mitochondrial biogenesis in LPS-induced AKI. **A** Double-immunofluorescent staining of β-catenin (red) and PGC-1α (green) in kidney cortex from TubCat-LPS mice. DAPI (blue) reveals nuclear staining. Yellow arrow denotes the co-localization of β-catenin and PGC-1α. Scale bar = 100 μm. **B** Immunofluorescent staining of PGC-1α (green) in kidney cortex from TubCatKO-LPS mice. Yellow arrow indicates PGC-1α-positive tubule. **C** Representative western blots and their quantification of mitochondrial marker TIM23 and mitochondrial biogenesis regulators (PGC-1α, NRF1) in kidney cortical lysates in TubCat-LPS mice. *p < 0.05; *n* = 5 in each group. **D** Representative western blots and their quantification of TIM23, PGC-1α and NRF1 in kidney cortical lysates in TubCatKO-LPS mice. *p < 0.05; ns, no significance. *n* = 5 in each group. **E**, **F** Representative TEM images in TubCat-LPS mice (**E**) and TubCatKO-LPS mice (**F**). Yellow arrowheads are swollen mitochondria, red arrowheads refer to intact mitochondria. N, nucleus. Scale bar = 2 μm. **G**, **H** representative western blot of FOXO3 in kidney nuclear fraction of TubCat-LPS and TubCatKO-LPS group, HDAC1 was used as nuclear protein loading control. *p < 0.05. *n* = 5 in each group.

Similar rescue of mitochondrial function was reproduced in the LPS-induced septic AKI model (Fig. 6 and Suppl Fig. S1), although the mitochondrial phenotype was not different between KO CTL-LPS and TubCatKO-LPS kidneys.

### β-catenin regulates tubular mitochondrial dynamics in AKI

**Fig. 7:**
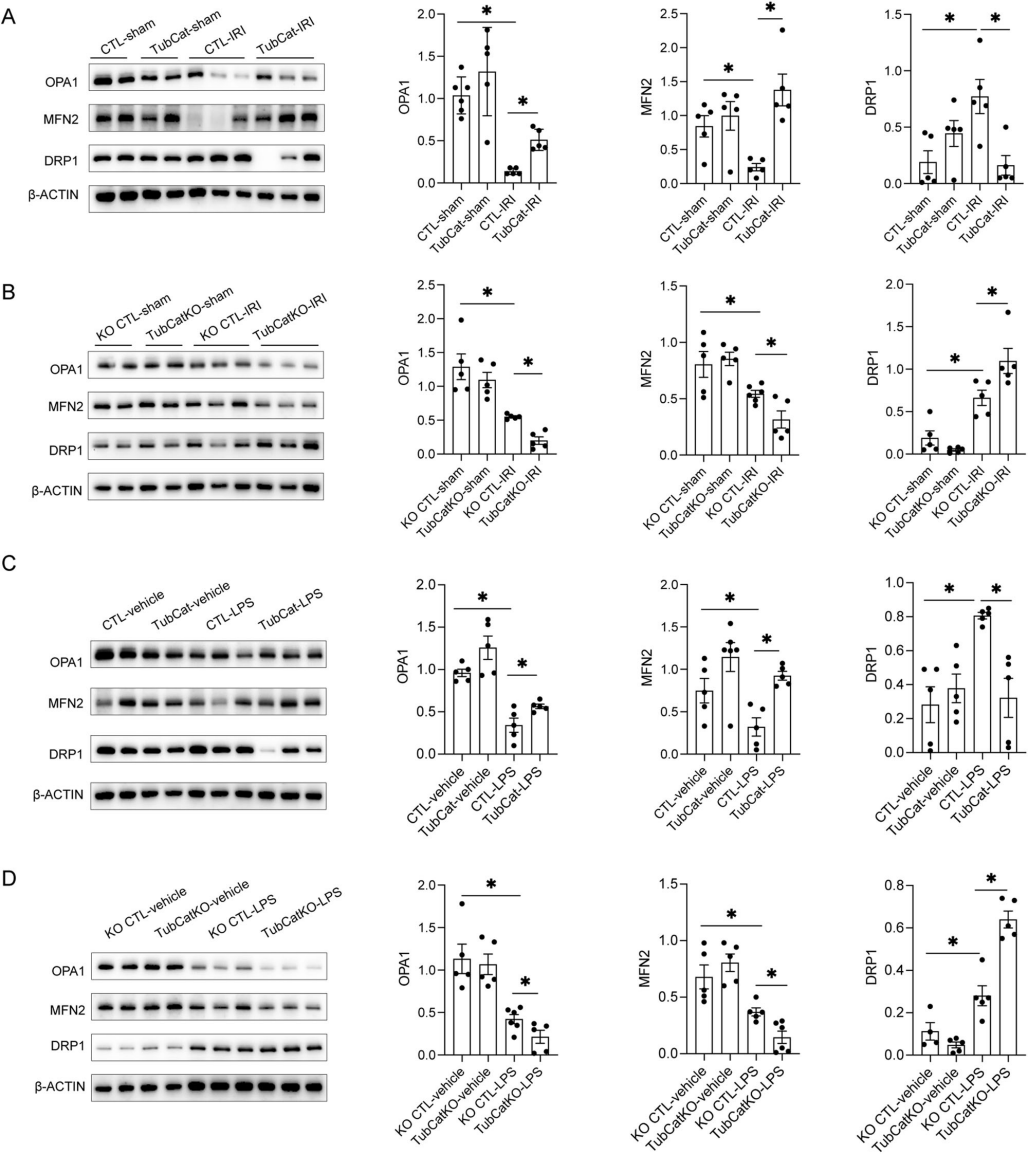
β-catenin regulates tubular mitochondrial dynamics in IRI-induced- and LPS-induced AKI. **A** Representative western blots for mitochondrial fusion markers (OPA1, MFN2) and mitochondrial fission protein DRP1 and their quantitation in TubCat-IRI mice. **B** Representative western blots for OPA1, MFN2, and DRP1 and their quantification in TubCatKO-IRI mice. **C** Representative western blots for mitochondrial fusion markers (OPA1, MFN2) and mitochondrial fission protein DRP1 and their quantitation in TubCat-LPS mice. **D** Representative western blots for OPA1, MFN2, and DRP1 and their quantitation in TubCatKO-LPS mice. *p < 0.05; *n* = 5 in each group.

Fusion-fission dynamics are critical in maintaining mitochondrial homeostasis. Οptic atrophy 1 (OPA1) and mitofusin-2 (MFN2) are two molecules regulating mitochondrial fusion, and dynamin-related protein 1 (DRP1) is a GTPase that controls mitochondrial fission. Mitochondrial dynamics shifts towards fission during IRI with downregulation of fusion regulators OPA1 and MFN2, but upregulation of fission regulator DRP1 (Fig. 7). In TubCat-IRI kidneys, markedly reduced OPA1 and MFN2 was restored, and DRP1 overexpression was suppressed vs. CTL-IRI kidneys (Fig. 7A). Conversely, TubCatKO-IRI kidneys had further reduction of OPA1 and MFN2 and higher DRP1 levels than KO CTL-IRI mice (Fig. 7B). Similar changes in mitochondrial dynamics and its regulation by tubular β-catenin AKI were reproduced in the LPS-induced AKI model (Fig. 7C, D).

### β-catenin regulates mitochondrial biogenesis via FOXO3/PGC-1α axis in vitro

**Fig. 8:**
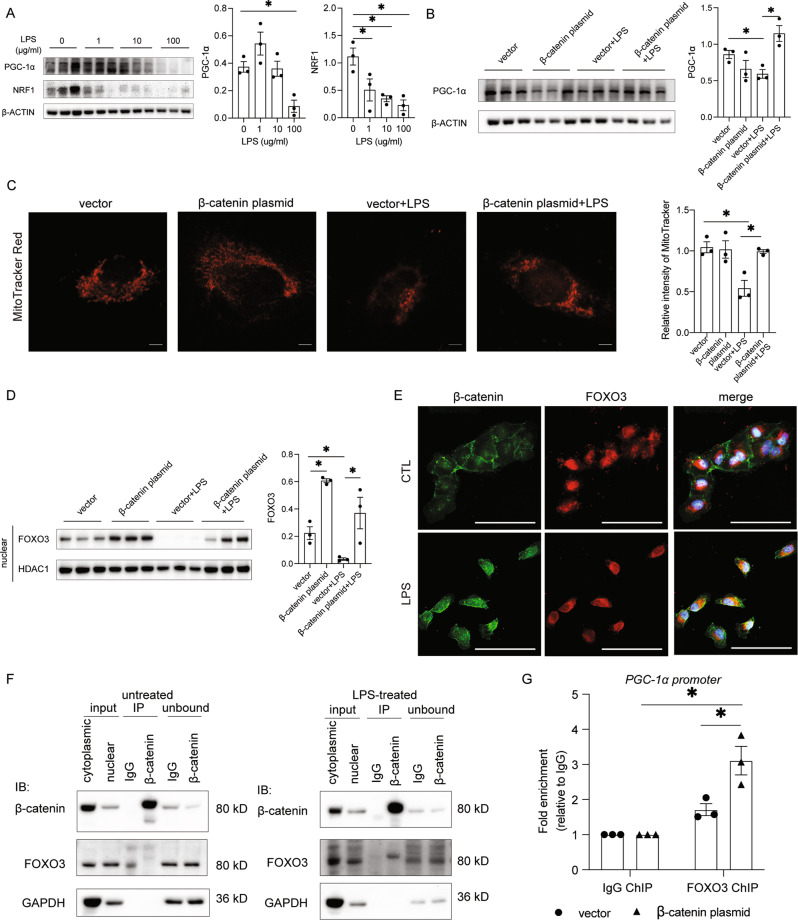
β-catenin enhanced FOXO3-dependent PGC-1α transcription that drives mitochondrial biogenesis upon LPS treatment in HK-2 cells. **A** PGC-1α, NRF1 protein expression and quantification upon different dose of LPS (0, 1, 10, and 100 μg/ml) for 3 h. *p < 0.05; *n* = 3 in each group. **B** Representative western blot and quantification on PGC-1α expression in vector or β-catenin transfected HK-2 cell lysate under LPS stimulation. *p < 0.05; *n* = 3 in each group. **C** Representative MitoTracker Red staining and intensity quantification in vector or β-catenin plasmid transfected HK-2 cells under LPS stimulation. Scale bar = 5 μm. *p < 0.05; *n* = 3 in each group. **D** Nuclear FOXO3 expression and quantification in HK-2 cell nuclear fraction upon β-catenin overexpression. *p < 0.05; *n* = 3 in each group. **E** Co-immunofluorescence staining of β-catenin (green) and FOXO3 (red) in HK-2 cells with or without LPS stimulation (100 μg/ml, 30 min). DAPI (blue) is used for nuclear staining. Scale bar = 100 μm. **F** Co-immunoprecipitation result on nuclear fraction of β-catenin overexpressing HK-2 cell in untreated or LPS-treated condition with β-catenin antibody and immunoblots with FOXO3. **G** Chromatin immunoprecipitation-qPCR showed FOXO3 binding to the promoter region (−1242 from TSS) of PGC-1α in HK-2 cells with β-catenin overexpression. *p < 0.05; *n* = 3 in each group. TSS transcription starting site.

In HK-2 cells exposed to LPS, the mitochondrial biogenesis proteins PGC-1α and NRF1 were reduced in a dose-dependent manner (Fig. 8A). In HK-2 cells transfected with a β-catenin-4A mutant plasmid to stabilize β-catenin expression with high efficiency (Suppl Fig. S2A, B), LPS treatment could no longer reduce PGC-1α expression (Fig. 8B). Likewise, reduction of mitochondrial mass, determined by MitoTracker Red fluorescence intensity, upon LPS treatment was abolished in β-catenin stabilized HK-2 cells (Fig. 8C).

To ascertain the mechanism through which tubular β-catenin maintains mitochondrial biogenesis, we studied FOXO3 expression in nuclear lysates of LPS-treated HK-2 cells (Suppl Fig. S2C). After 30 min of LPS exposure, nuclear FOXO3 decreased significantly, and this was not only prevented but even enhanced in β-catenin stabilized HK-2 cells vs. vector control (Fig. 8D).

### β-catenin interacts with FOXO3 and enhanced FOXO3-dependent PGC-1α transcription in HK-2 cells

To further dissect the mechanism of β-catenin enhancing FOXO3/PGC-1α, double-immunofluorescence staining on β-catenin and FOXO3 and co-IP assay were performed in HK-2 cells under control condition or LPS stimulation (100 ng/µl, 30 min). β-catenin is mainly expressed in the cytoplasm and cell-cell junctions under the unstimulated condition, whereas FOXO3 is predominantly expressed in the nucleus (Fig. 8E). After LPS stimulation for 30 min, β-catenin was activated and translocated into the nucleus, whereas FOXO3 was reduced and remained in the nucleus (Suppl Fig. S2C), and co-localized with β-catenin to form a FOXO3/β-catenin complex detected by co-IP (Fig. 8F). In untreated cells, there was no detectable FOXO3/β-catenin interaction.

To investigate whether such binding affected the downstream FOXO3/PGC-1α signaling, which regulates mitochondrial biogenesis, FOXO3 ChIP antibody or IgG antibody was used for immunoprecipitation of the fragmented chromatin of HK-2 cells under vector or β-catenin plasmid transfection. ChIP output DNA and input DNA were used as the template in ChIP-qPCR with three predicted FOXO3-binding PGC-1α promoter primer sets (Suppl Table S1). A 3-fold enrichment of PGC-1α promoter binding of FOXO3 ChIP antibody was detected in cells that overexpressed β-catenin compared with vector-treated cells (Fig. 8G and Suppl Fig. S3).

## Discussion

For the first time, we showed a protective role of kidney tubular β-catenin in AKI by using tubule-specific loss- and gain-of-β-catenin function in septic and aseptic models. β-catenin signaling in tubular cells protects the kidney from cell death and restores mitochondrial homeostasis. We also unraveled a novel cellular mechanism mediated by the FOXO3/PGC-1α axis in which a nuclear β-catenin/FOXO3 complex is formed to activate PGC-1α transcription followed by restoration of mitochondrial biogenesis during AKI.

Despite a consensus that β-catenin is protective during AKI, there are significant limitations from studies using β-catenin knockout mice or pharmacological intervention due to the congenital and non-specific nature of β-catenin ablation or off-target effects of pharmacological inhibition [10, 12, 30].

Here, we tackled this shortcoming by adopting a loss- and gain-of-β-catenin function strategy by generating a transgenic mouse with tubule-specific β-catenin stabilization and ablation to validate the role of β-catenin in AKI [25]. We extended previous findings by showing that tubule-specific β-catenin stabilization not only mitigated apoptosis of tubular cells, but also alleviated necroptosis. Necroptosis contributes to the pathophysiology of different models of AKI induced by IRI, iodinated contrast, cisplatin, and folic acid [31–34]. The therapeutic efficacy of pan-caspase inhibition in experimental kidney IRI is controversial, suggesting apoptosis is not the only culprit in its pathogenesis [35, 36]. By contrast, treatment with a necroptosis inhibitor ameliorated IRI-induced tubular injury and improved kidney function [35]. Moreover, RIP3 knockout mice were protected from IR [37], suggesting that necroptosis might be an alternative cell death pathway in kidney IRI. Nevertheless, targeting necroptosis per se does not offer full protection in AKI, suggesting that multiple pathways contribute to kidney injury. Tubular cells are more vulnerable to necroptosis during IRI [35] and the involvement of β-catenin has yet to be proven. In IRI-induced hepatocellular injury, inhibition of FOXO1/β-catenin signaling attenuated RIP3-mediated necroptosis [38]. Our data showed that stabilization of β-catenin activity reduced the expression of necroptosis execution molecules p-MLKL and p-RIP3, probably via AKT/p53 signaling. This is supported by observations that AKT and p53 also mediated necroptosis in myocardial infarction [39] and stroke [40]. Our study is the first to report an anti-necroptotic effect of β-catenin in AKI via activating AKT signaling to suppress p53. Although the degree of cell death in murine experimental AKI models seem to differ from that in human AKI [41, 42], critically ill patients with AKI are unlikely biopsied and there is a knowledge gap in the degree of tissue insult in such patients. As such, the changes in the severity of cell death in aseptic/septic TubCat and TubCatKO mice do shed light on the role of β-catenin against apoptosis and necroptosis.

Given their high metabolic demand, tubular cells have the greatest number of mitochondria in the kidney, and they are the most vulnerable target for AKI, which compromises mitochondrial energetics. For decades, a dysmorphic mitochondrial ultrastructure has been observed in kidney tubules after AKI [18, 19, 43]. Transient ischemia in the human kidney led to mild structural changes other than mitochondrial swelling [44], whereas persistent disruption of mitochondrial homeostasis leads to an adverse kidney outcome [45]. Mitochondrial fission or fragmentation is an early event that precedes tubular cell death in AKI [45, 46], while sustained mitochondrial dysfunction propagates cellular injury through releasing mtDNA, mitochondrial ROS, and pro-apoptotic factors. In cisplatin-induced kidney injury, activation of β-catenin alters mitochondrial homeostasis, leading to disturbance of mitochondrial redox balance and mitochondria-dependent apoptosis [13].

Our data shows that perturbation of mitochondrial dynamics in AKI was partly corrected by β-catenin. Tubular stabilization of β-catenin promoted mitochondrial fusion and reduced mitochondrial fragmentation as indicated by increased MFN2, OPA1, and decreased DRP1 expression. In agreement with these results, the reverse was found in tubular β-catenin knockout mice, which confirmed a mitochondrial protective function of β-catenin signaling. In addition, tubular β-catenin signaling regulates PGC-1α expression, which is reported to be decreased in various AKI models (IRI, septic, folic acid, and cisplatin) [18, 24, 47, 48]. Enhancing PGC-1α expression by pharmacological strategies shows a protective effect in AKI [46, 49]. Direct evidence showing the participation of β-catenin in modulation of PGC-1α expression came from cancer or developmental studies. In breast cancer cells, knockdown of β-catenin repressed PGC-1α expression [50]. During endodermal differentiation, pharmacological inhibition of GSK3β led to β-catenin activation and PGC-1α upregulation [51]. In cisplatin-induced AKI, Wnt/β-catenin inhibition repressed PGC-1α expression. In rat tubular cell line with β-catenin knockdown, there was downregulated expression of PGC-1α and mitochondrial biogenesis transcription factors TFAM and NRF1 [13]. To our knowledge, this is the first in vivo study establishing a link between β-catenin and PGC-1α in AKI. Our data show that in both septic and aseptic AKI models, restored PGC-1α co-existed with increased β-catenin in the same kidney tubules of TubCat mice, suggesting PGC-1α derangement to represent a common mechanism that propagates kidney injury.

The cyclic adenosine monophosphate (cAMP) response element-binding protein (CREB) has been proposed as a positive regulator of PGC-1α transcription in neural cells [52, 53]. In lung cancer, β-catenin interacts with CREB to promote cell proliferation [54]. In squamous cell carcinoma, β-catenin regulates its downstream effector CREB to promote cell growth [55]. However, our results did not show that β-catenin acts on CREB to modulate PGC-1α expression in AKI (Supplemental Fig. S4).

FOXO3 belongs to the FOXO family, which regulates vital cellular processes including metabolism, proliferation, stress resistance, and apoptosis [56–59]. In *C. elegans*, the FOXOs transcription factor DAF-16 requires the β-catenin gene, *bar-1*, for longevity [60], suggesting β-catenin–FOXOs interaction might be pivotal for mammals’ survival. FOXO competes with TCF for β-catenin interaction [61]. Shifting the binding partner of β-catenin from TCF to FOXO alleviated unilateral ureteric obstruction- and ischemia-induced kidney fibrosis [17, 62]. The FOXO3 subcellular localization in response to β-catenin accumulation has not been fully understood. In this study, β-catenin overexpression alone in HK-2 cells could not induce β-catenin–FOXO3 interaction. With LPS stimulation, β-catenin was activated and entered the nucleus to form a β-catenin–FOXO3 interaction. Oxidative stress induces β-catenin–FOXO3 interaction in proximal tubular cells [16], suggesting that β-catenin–FOXO3 is disease-dependent. Nuclear FOXO3 was upregulated with β-catenin stabilization and downregulated upon β-catenin ablation in murine AKI. The β-catenin–FOXO3 interaction retained FOXO3 in the nucleus and prevented it from translocating into the cytoplasm for degradation, thus setting up a positive feedback loop to promote nuclear accumulation of β-catenin [63]. In endothelial cells, FOXO3 interacted with PGC-1α to initiate the transcription of antioxidant genes [64]. Deacetylation of FOXO3 and PGC-1α stabilized the PGC-1α/FOXO3 transcriptional complex to deliver an antioxidative effect in endothelial cells [65]. These observations prompted us to examine the mechanism of β-catenin-induced PGC-1α expression via FOXO3 in AKI. Significant fold enrichment of FOXO3 binding to PGC-1α promoter was enhanced by stabilizing β-catenin in HK-2 cells, which also increased PGC-1α expression and preserved mitochondrial mass. These findings suggest that β-catenin activates mitochondrial biogenesis by increasing PGC-1α transcription via FOXO3. To our knowledge, this report is the first on the protective role of the β-catenin/FOXO3/PGC-1α axis in AKI-induced mitochondrial dysbiogenesis.

Although the protective effect of β-catenin accumulation was ascertained in TubCat-IRI and TubCat-LPS models, the detrimental effect of β-catenin deficiency in renal tubules remained debatable in AKI models. In TubCatKO mice, IRI only marginally increased sCr and kidney function loss was not different between KO CTL-LPS and TubCatKO-LPS mice. Histologically, TubCatKO mice had slightly higher proportion of severely damaged tubules than KO CTL mice in both septic and aseptic AKI models. Nevertheless, the acute tubular injury marker (NGAL), cell death molecules, and mitochondrial-related proteins showed a significant difference between TubCatKO and KO CTL mice. One explanation for this discrepancy could be that the reduction in β-catenin levels in TubCatKO-IRI/LPS vs. KO CTL-IRI/LPS mice was only sufficient to affect mitochondrial function and cell death, but not kidney function. The discrepancy in kidney function between the IRI and LPS models could arise from the fact that IRI is a direct ischemia injury to the kidney, whereas LPS is a systemic inflammatory syndrome that induces a more subtle in situ injury to the kidney. It is therefore not surprising that IRI induced more kidney function loss and histologic damage than LPS. Another explanation might be the relatively short timepoint (24 h). A longer timepoint, up to 48 h or even 7 days, might cast a more global view of the effect of tubular β-catenin deficiency in AKI. Finally, the discrepancy in TUNEL-positive cells between TubCat and TubCatKO mice in the IRI model could arise from their heterogeneous genetic background that affects their propensity to apoptosis induced by IRI.

**Fig. 9:**
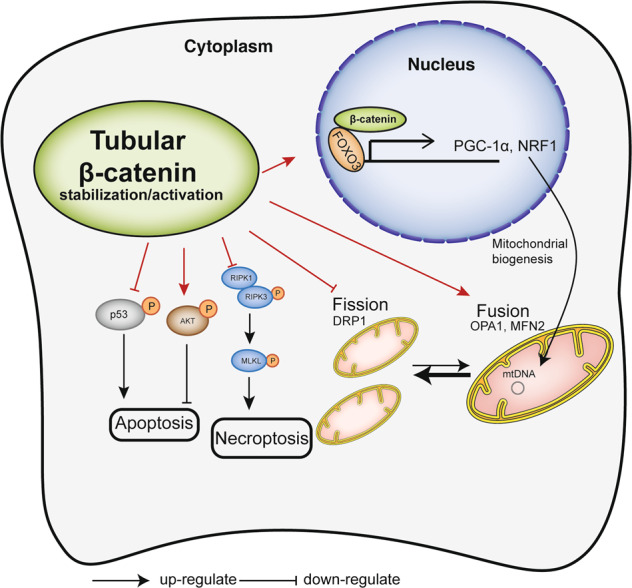
Proposed mechanism of β-catenin regulating cell death and mitochondrial function in kidney tubule. Exposed to ischemia or LPS stress leads to kidney tubular mitochondrial dysfunction and cell death. As β-catenin stabilize in tubular cells, more active β-catenin translocates into the nucleus, enhances PGC-1α transcription and activation through its binding and interacting with FOXO3 to form a transcription factor co-activator complex. Downstream gene NRF1 expression increase to active mitochondrial biogenesis. Mitochondrial dynamic remodels towards mitochondrial fusion than fission. Consequently, mitochondrial mass and structure are preserved. In addition, β-catenin stabilization also ameliorates necroptosis via RIP3/MLKL pathway and apoptosis via AKT/p53 signaling.

In conclusion, tubular β-catenin activation alleviates septic and aseptic AKI by reducing kidney cell death and restoring mitochondrial biogenesis via β-catenin/AKT/p53 and β-catenin/FOXO3/PGC-1α signaling pathways. A schema for the protective mechanism is shown in Fig. 9. Further efforts are needed to render the β-catenin/FOXO3/PGC-1α axis amendable to manipulation as a therapeutic target of AKI.

## Supplementary information

Supplemental Materials

Original Western blots

## Data Availability

All data from this work are available upon request.
