# Supplementary material for: Tubular β-catenin alleviates mitochondrial dysfunction and cell death in acute kidney injury
Source: Cell Death Dis. 2022 Dec 20;13(12):1061. doi: 10.1038/s41419-022-05395-3 (PMC9768165; doi:10.1038/s41419-022-05395-3)
Supplement: Supplementary file 1 — Supplemental Materials [file 41419_2022_5395_MOESM1_ESM.docx]

**Supplemental material Table of Contents**

Supplemental Table S1. Primer sequences

Supplemental Table S2. Injured tubule percentage in AKI models

Supplemental Figure S1. Tubular β-catenin maintains mitochondrial mtDNA copy number and ATP levels in AKI

Supplemental Figure S2. Supplemental data on in vitro study

Supplemental Figure S3. β-catenin interaction with FOXO3 and promote PGC-1α transcription

Supplemental Figure S4. Tubular β-catenin fail to regulate CREB in IRI-induced AKI

**Supplemental Table S1. Primer sequences**

| Name | Sequence | Application |
| --- | --- | --- |
| *KspCre^ERT2^* |  | genotyping |
| Forward | 5'-AGGTTCGTGCACTCATGGA-3' |  |
| Reverse | 5'-TCGACCAGTTTAGTTACCC​-3' |  |
| *Catnb^lox(ex3)/wt^* |  | genotyping |
| Forward | 5'-AGGTTCGTGCACTCATGGA-3' |  |
| Reverse | 5'-TCGACCAGTTTAGTTACCC​-3' |  |
| *Catnb^lox/lox^* |  | genotyping |
| catnbyc1F | 5'-CAGCAAGCCACCGATGGGATC-3' |  |
| catnbyc1B | 5'-CTGAAAATGCTACCTGAAGAAGC-3' |  |
| catnbyc2B | 5'-CTCCCTCACCCTTAAGGTCCTT-3' |  |
| *MuPgc-1α* |  | Real time PCR |
| Forward | 5'-CAACAATGAGCCTGCGAACA-3' |  |
| Reverse | 5'-CTTCATCCACGGGGAGACTG-3' |  |
| *MuNrf1* |  | Real time PCR |
| Forward | 5'-GCACCTTTGGAGAATGTGGT-3' |  |
| Reverse | 5'-CTGAGCCTGGGTCATTTTGT-3' |  |
| *MuOpa1* |  | Real time PCR |
| Forward | 5'-TACCACAGTCCGGAAGAACC-3' |  |
| Reverse | 5'-ATTCGCCAAAACAGGACCAC-3' |  |
| *MuMfn2* |  | Real time PCR |
| Forward | 5'-ATGTTACCACGGAGCTGGAC-3' |  |
| Reverse | 5'-AACTGCTTCTCCGTCTGCAT-3' |  |
| *MuDrp1* |  | Real time PCR |
| Forward | 5'-GCAACTGGAGAGGAATGCTG-3' |  |
| Reverse | 5'-CACAATCTAGCTGTTCTCGG-3' |  |
| *MuCreb* |  | Real time PCR |
| Forward | 5'-TCAGCCGGGTACTACCATTC-3' |  |
| Reverse | 5'-CTCTCTTCCGTGCTGCTTCT-3' |  |
| *PGC-1α promoter primer -1242 from TSS* | | ChIP-qPCR |
| Forward | 5'-AACTGGGGGATTGTTTTCAGGTA-3' |  |
| Reverse | 5'-TTAACCCTTCCAAGTTCCCAGG-3' |  |
| *PGC-1α promoter primer -624 from TSS* | | ChIP-qPCR |
| Forward | 5'-TTCGGGAGCTGGTATTCCCTA-3' |  |
| Reverse | 5'-AAGGTATAAACAAACTCCTCCACCC-3' |  |
| *PGC-1α promoter primer -1493 from TSS* | | ChIP-qPCR |
| Forward | 5'-CAGCTCTCACAGCACACAAATC-3' |  |
| Reverse | 5'-AAACATGGTTATTTGCCCTAACAC-3' |  |

**Supplemental Table S2. Injured tubule percentage in AKI models**

| **Groups** | **Intact** | | **Moderately damaged** | | **Severely damaged** | |
| --- | --- | --- | --- | --- | --- | --- |
|  | **Mean (n=5)** | **SEM** | **Mean (n=5)** | **SEM** | **Mean (n=5)** | **SEM** |
| **TubCat-IRI** |  |  |  |  |  |  |
| CTL-sham | 92.39 | 2.16 | 6.92 | 2.26 | 0.69 | 0.33 |
| TubCat-sham | 97.28 | 0.47 | 2.14 | 0.50 | 0.58 | 0.17 |
| CTL-IRI | 8.54* | 3.95 | 23.89* | 2.28 | 67.57* | 3.52 |
| TubCat-IRI | 20.86^#^ | 3.97 | 41.03^#^ | 5.53 | 38.11^#^ | 6.57 |
| **TubCatKO-IRI** |  |  |  |  |  |  |
| KO CTL-sham | 96.44 | 0.94 | 3.56 | 0.94 | 0.00 | 0.00 |
| TubCatKO-sham | 95.94 | 1.48 | 4.06 | 1.48 | 0.00 | 0.00 |
| KO CTL-IRI | 8.72* | 0.77 | 66.65* | 4.72 | 24.63* | 4.06 |
| TubCatKO-IRI | 8.33 | 1.21 | 53.76^#^ | 1.17 | 37.91^#^ | 1.02 |
| **TubCat-LPS** |  |  |  |  |  |  |
| CTL-vehicle | 100.00 | 0.00 | 0.00 | 0.00 | 0.00 | 0.00 |
| TubCat-vehicle | 100.00 | 0.00 | 0.00 | 0.00 | 0.00 | 0.00 |
| CTL-LPS | 49.47* | 3.42 | 37.31* | 3.88 | 13.22* | 0.73 |
| TubCat-LPS | 71.26^#^ | 2.86 | 19.84^#^ | 3.11 | 8.90^#^ | 0.89 |
| **TubCatKO-LPS** |  |  |  |  |  |  |
| KO CTL-vehicle | 99.50 | 0.34 | 0.50 | 0.34 | 0.00 | 0.00 |
| TubCatKO-vehicle | 98.90 | 0.74 | 1.10 | 0.74 | 0.00 | 0.00 |
| KO CTL-LPS | 49.62* | 4.37 | 41.16* | 3.88 | 9.22* | 1.16 |
| TubCatKO-LPS | 47.08 | 4.44 | 37.80 | 5.78 | 15.12^#^ | 1.56 |

*p<0.05 vs. sham or vehicle; #p<0.05 vs. IRI or LPS.

**Supplemental Figure S1. Tubular β-catenin maintains mitochondrial mtDNA copy number and ATP levels in AKI**

(A) mtDNA copy number in TubCat-IRI, TubCatKO-IRI, TubCat-LPS and TubCatKO-LPS. *p<0.05; ns = no significance; n=5 in each group. (B) ATP concentration per wet weight in kidney of TubCat-IRI, TubCatKO-IRI, TubCat-LPS and TubCatKO-LPS. *p<0.05; ns = no significance; n=4 in each group.

**Supplemental Figure S2. Supplemental data on in vitro study**

(A) β-catenin protein expression upon β-catenin plasmid transfection in HK-2 cells. *p<0.05; n=3 in each group. (B) Gene expression and quantification of β-catenin upon β-catenin plasmid transfection in HK-2 cells. *p<0.05; n=3 in each group. (C) Nuclear FOXO3 expression and quantification in HK-2 cell nuclear fractions upon LPS (100 μg/ml) stimulation at different time points. *p<0.05; n=3 in each group.

**Supplemental Figure S3. β-catenin interaction with FOXO3 and promote PGC-1α transcription**

(A) Chromatin immunoprecipitation-qPCR showed FOXO3 binding to the promoter region of PGC-1α (-624 from TSS) in HK-2 cells with β-catenin overexpression. *p<0.05; n=3 in each group. (B) Chromatin immunoprecipitation-qPCR showed FOXO3 binding to the promoter region (-1493 from TSS) of PGC-1α in HK-2 cells with β-catenin overexpression. *p<0.05; n=3 in each group. TSS: Transcription starting site.

**Supplemental Figure S4. Tubular β-catenin fail to regulate CREB in IRI-induced AKI**

(A) Representative Western blots for p-CREB and CREB in TubCatKO-IRI mice and its quantitation. (B) Gene expression of Creb in in TubCatKO-IRI mice. ns = no significance.
