# Supplementary material for: Tubular β-catenin alleviates mitochondrial dysfunction and cell death in acute kidney injury
Source: Cell Death Dis. 2022 Dec 20;13(12):1061. doi: 10.1038/s41419-022-05395-3 (PMC9768165; doi:10.1038/s41419-022-05395-3)

Review files– western blot

1. Fig 3C, p-MLKL

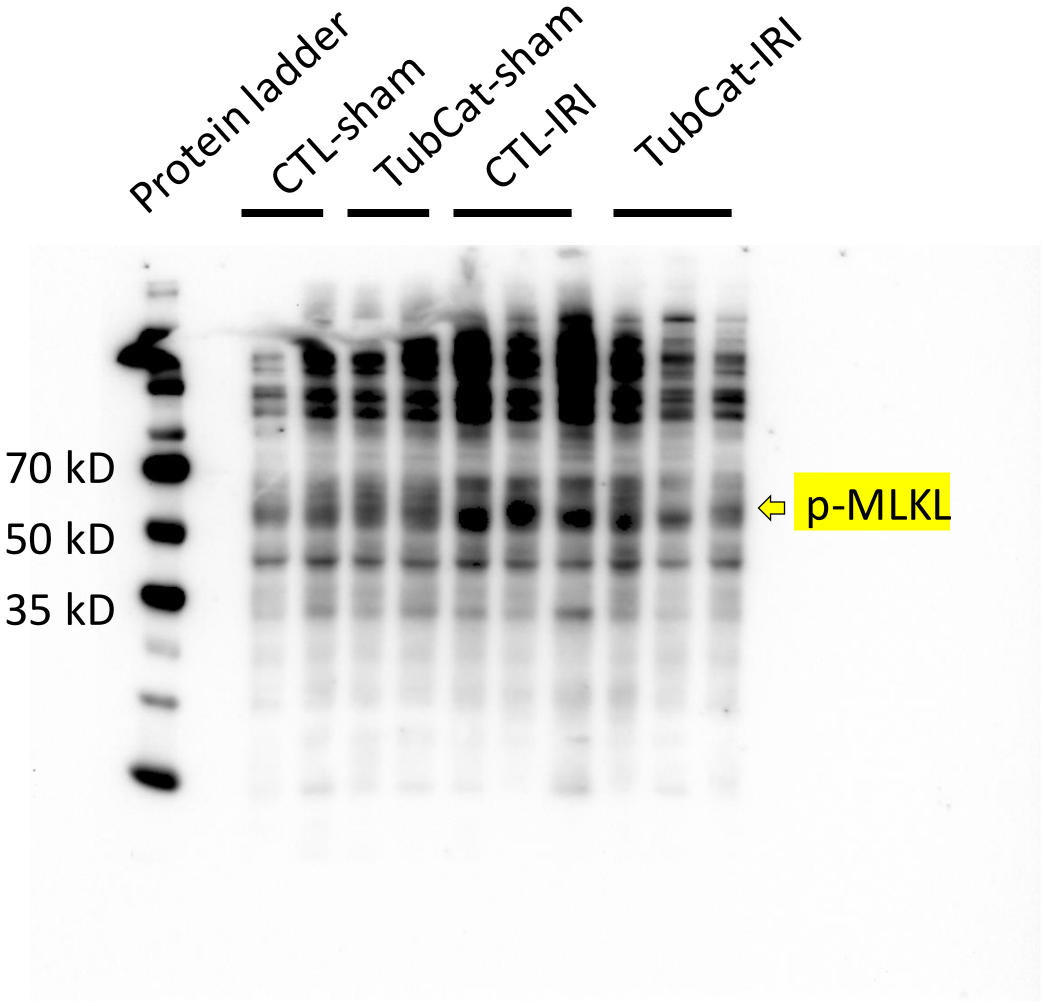

Review files– western blot

2. Fig 3C, β-ACTIN for p-MLKL

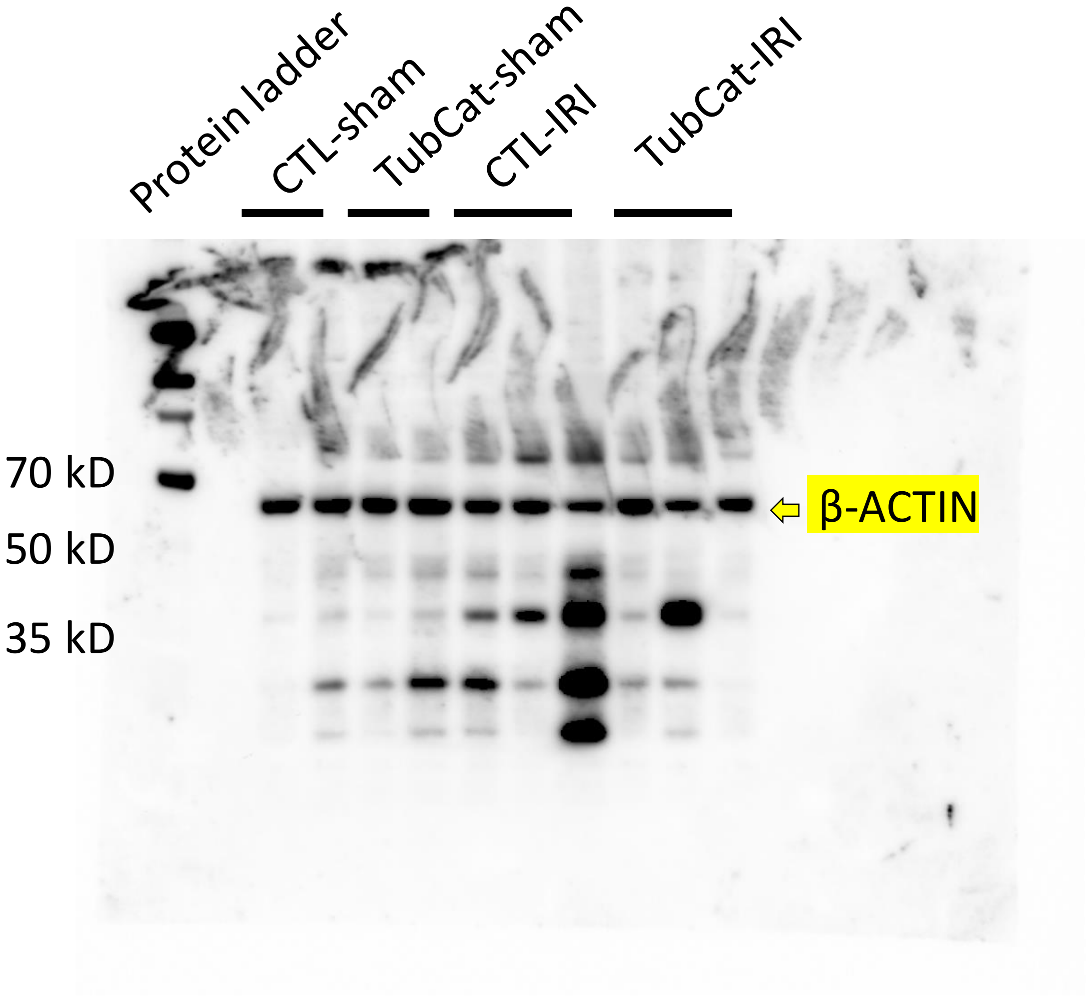

Review files– western blot

3. Fig 3C, p-RIP3

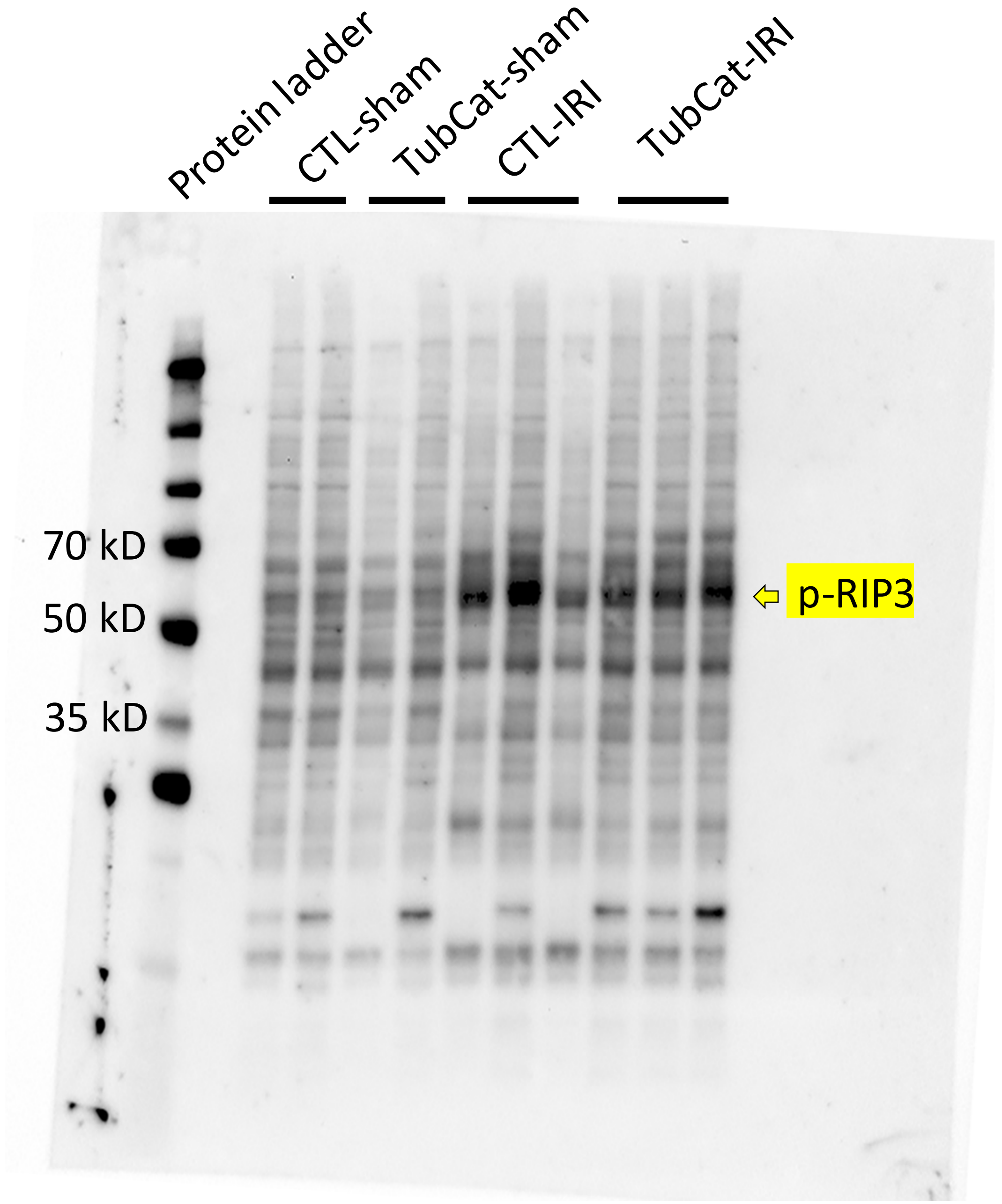

Review files– western blot

4. Fig 3C, β-ACTIN for p-RIP3

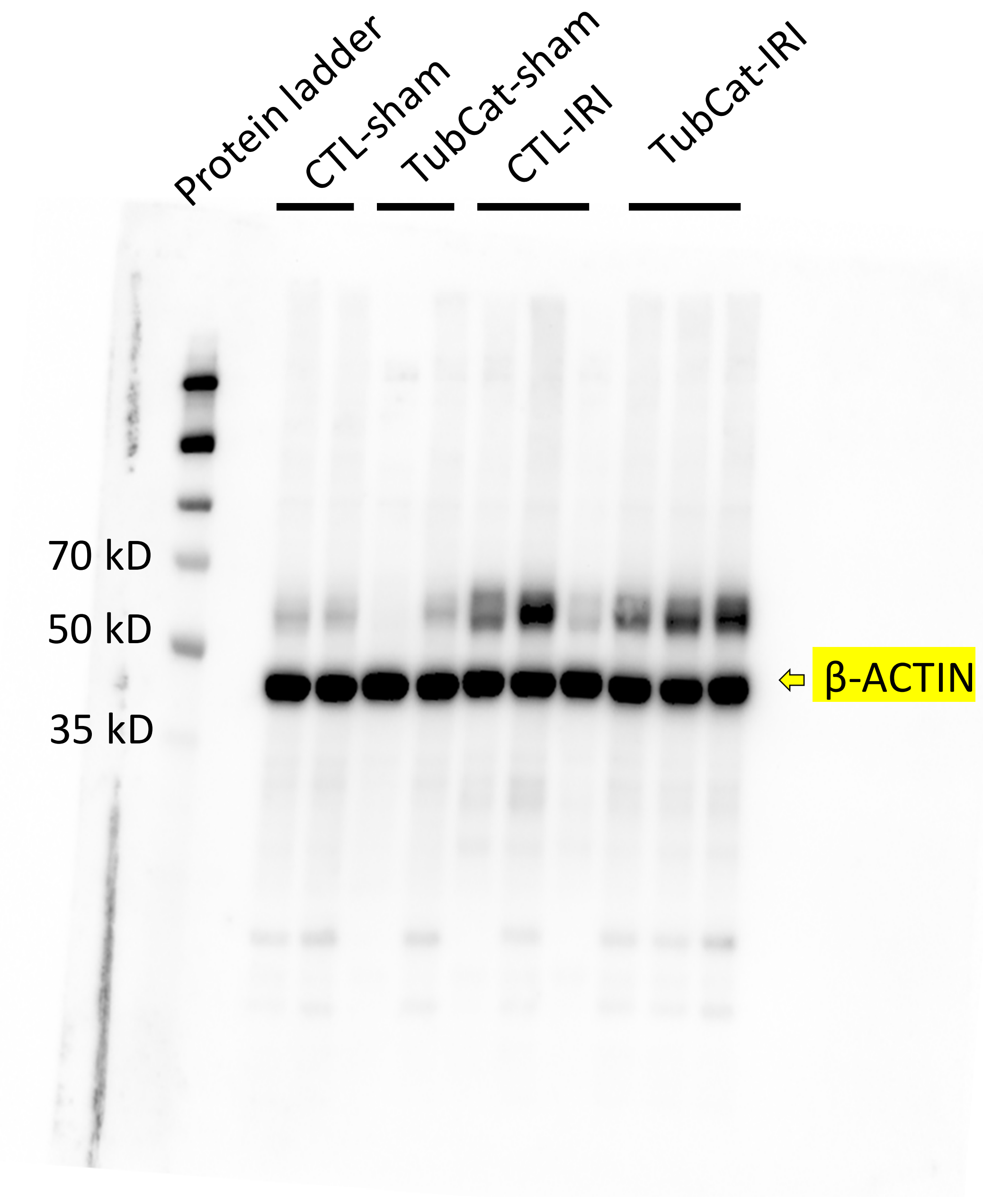

Review files– western blot

5. Fig 3D, p-MLKL

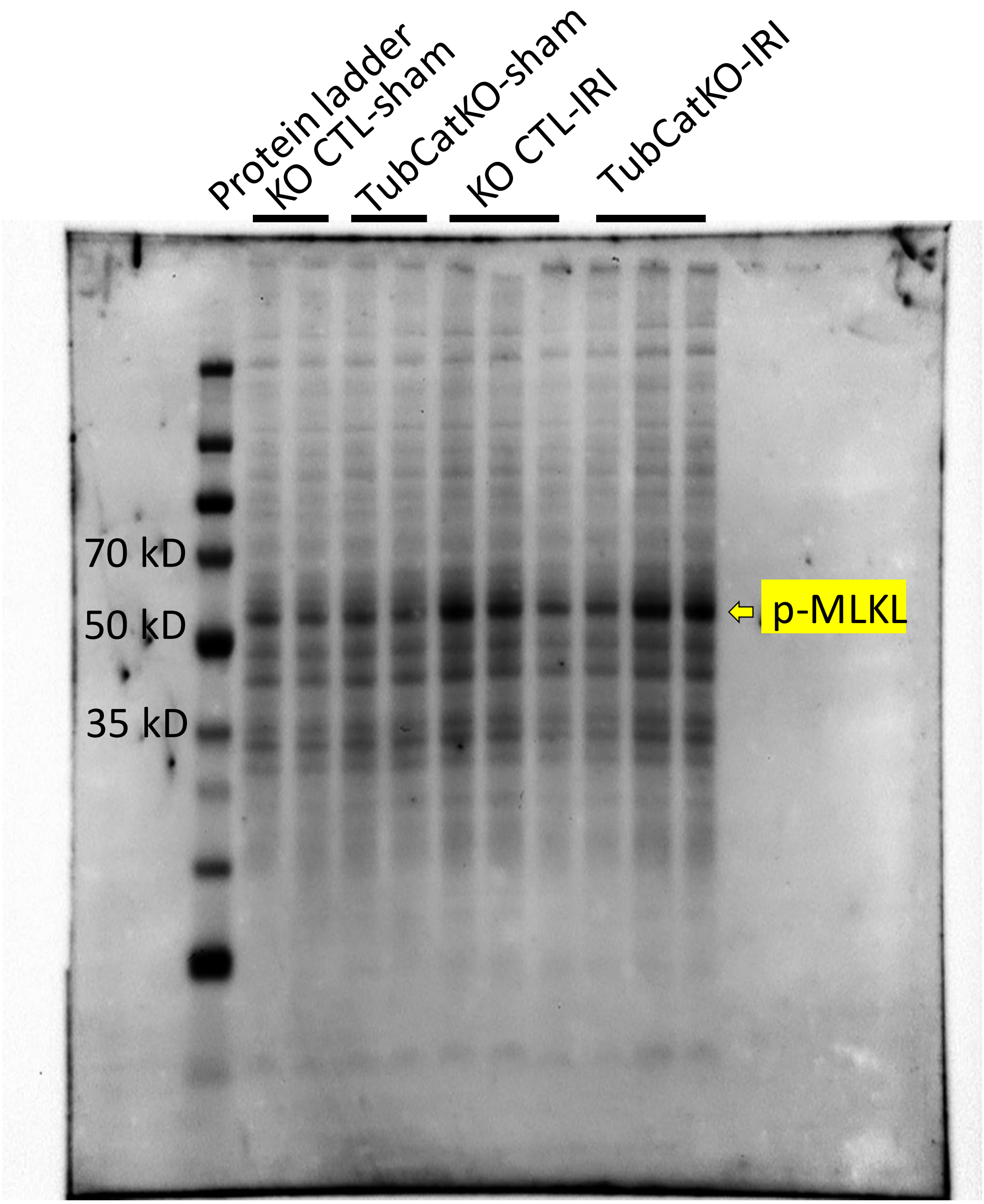

Review files– western blot

6. Fig 3D, β-ACTIN for p-MLKL

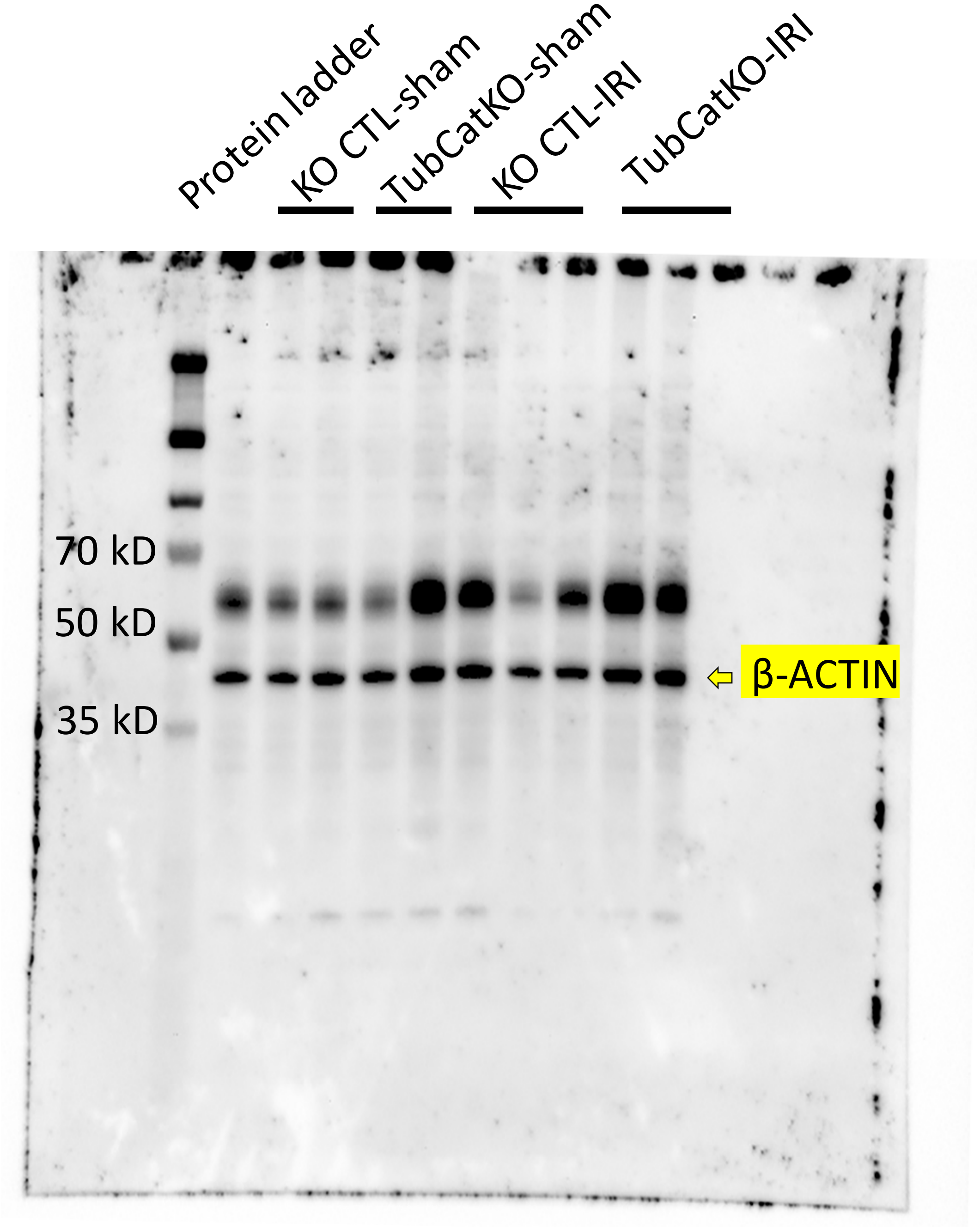

Review files– western blot

7. Fig 3D, p-RIP3

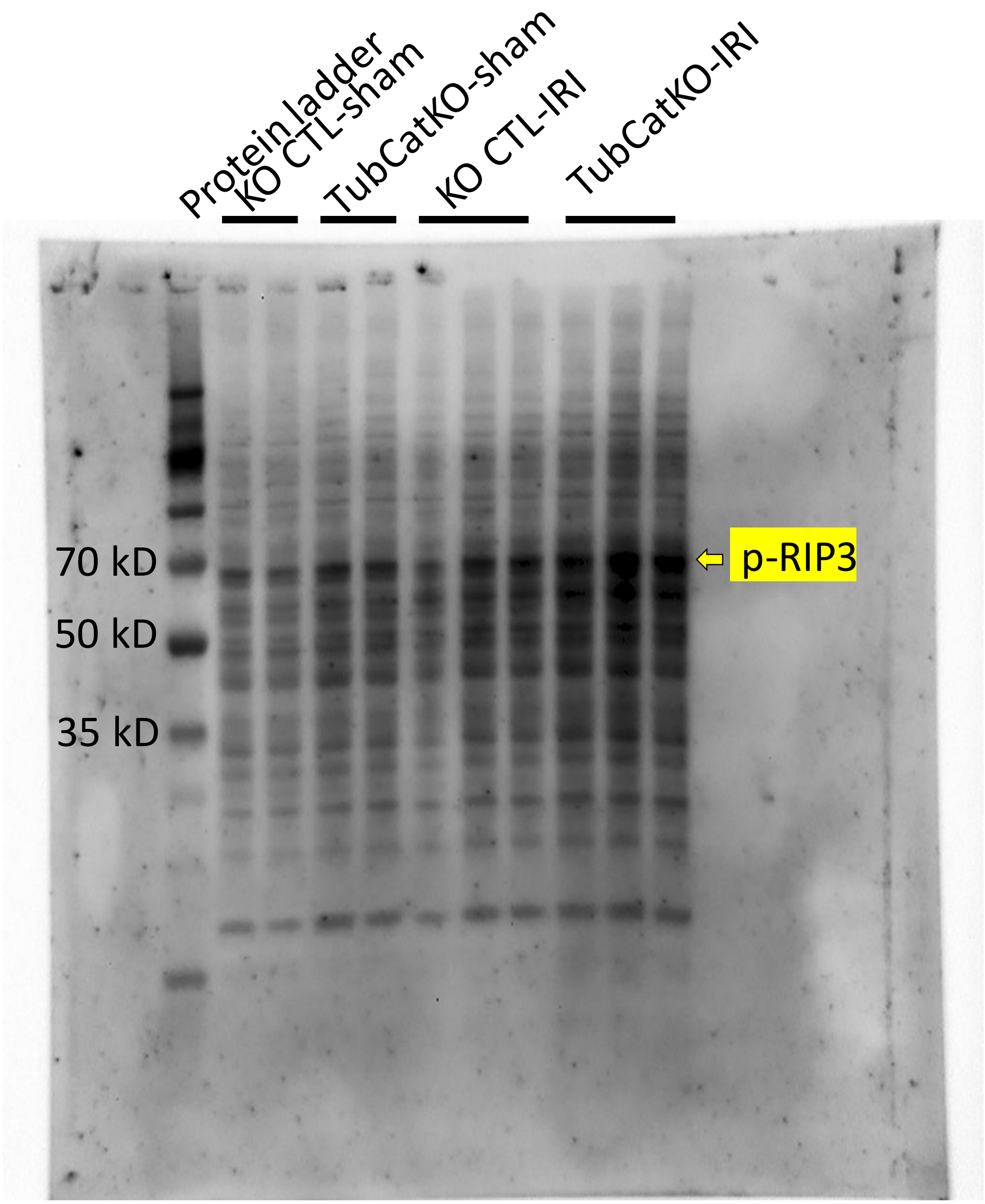

Review files– western blot

8. Fig 3D, β-ACTIN for p-RIP3

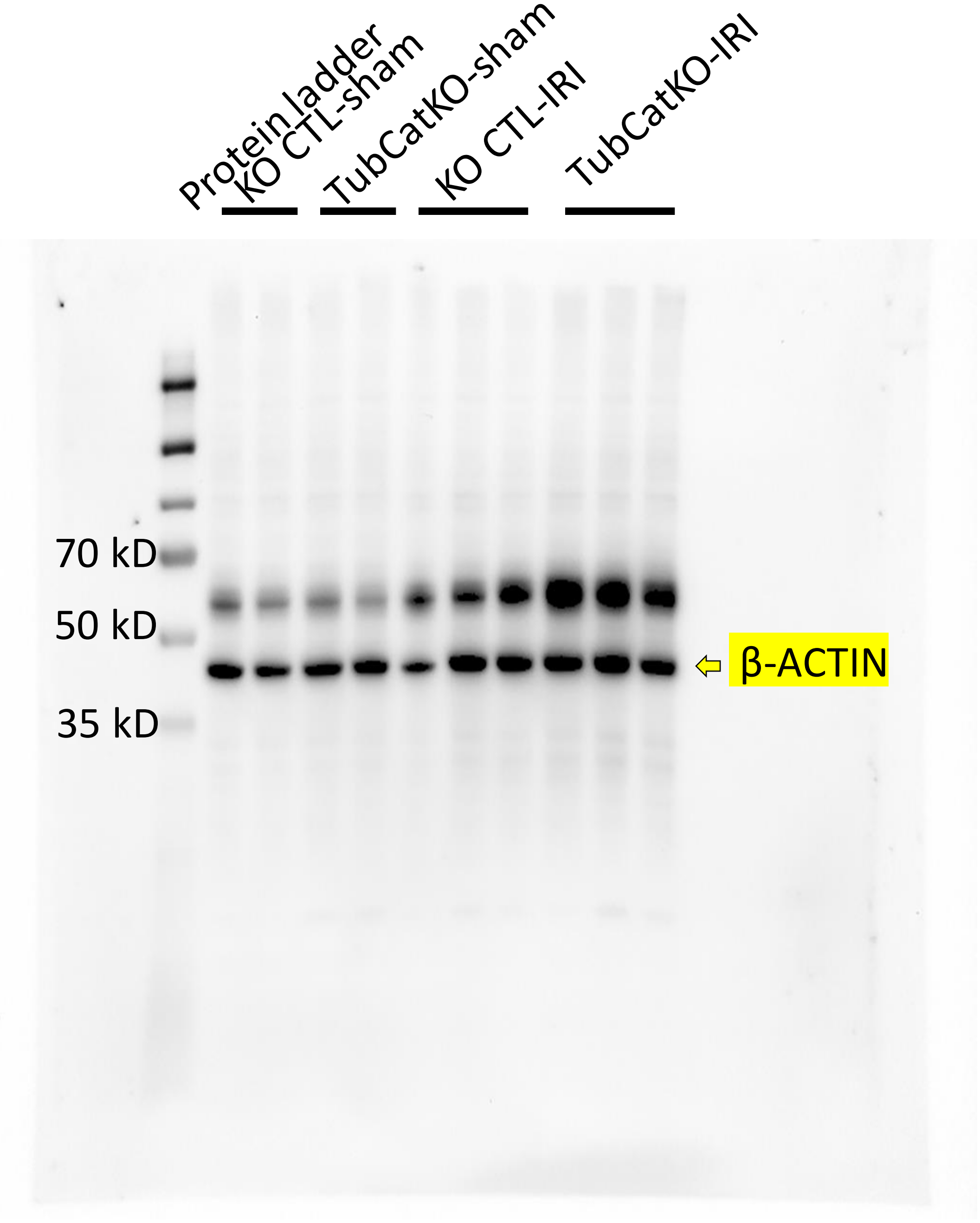

Review files– western blot

9. Fig 3E, p-AKT

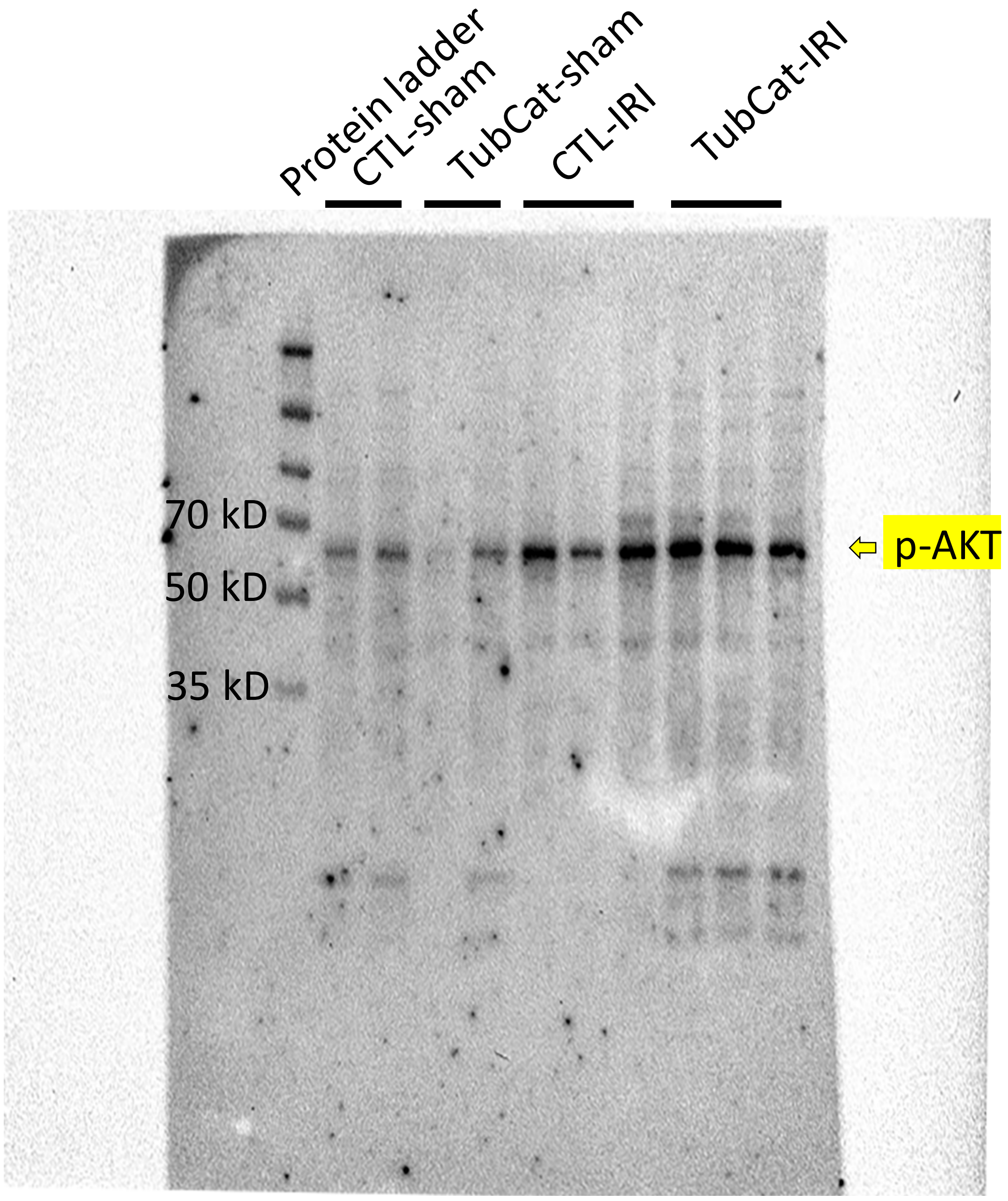

Review files– western blot

10. Fig 3E, AKT

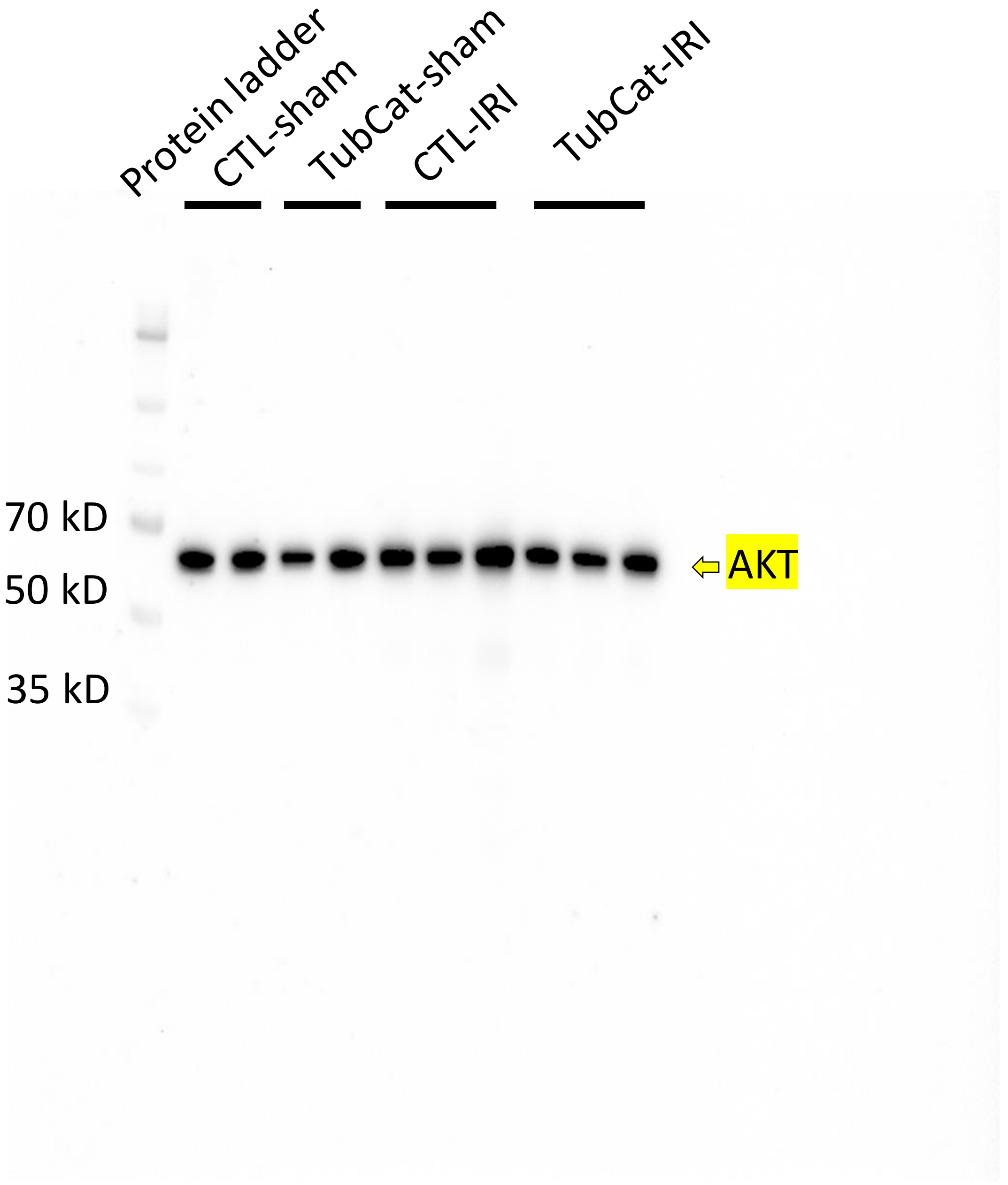

Review files– western blot

11. Fig 3E, p-p53

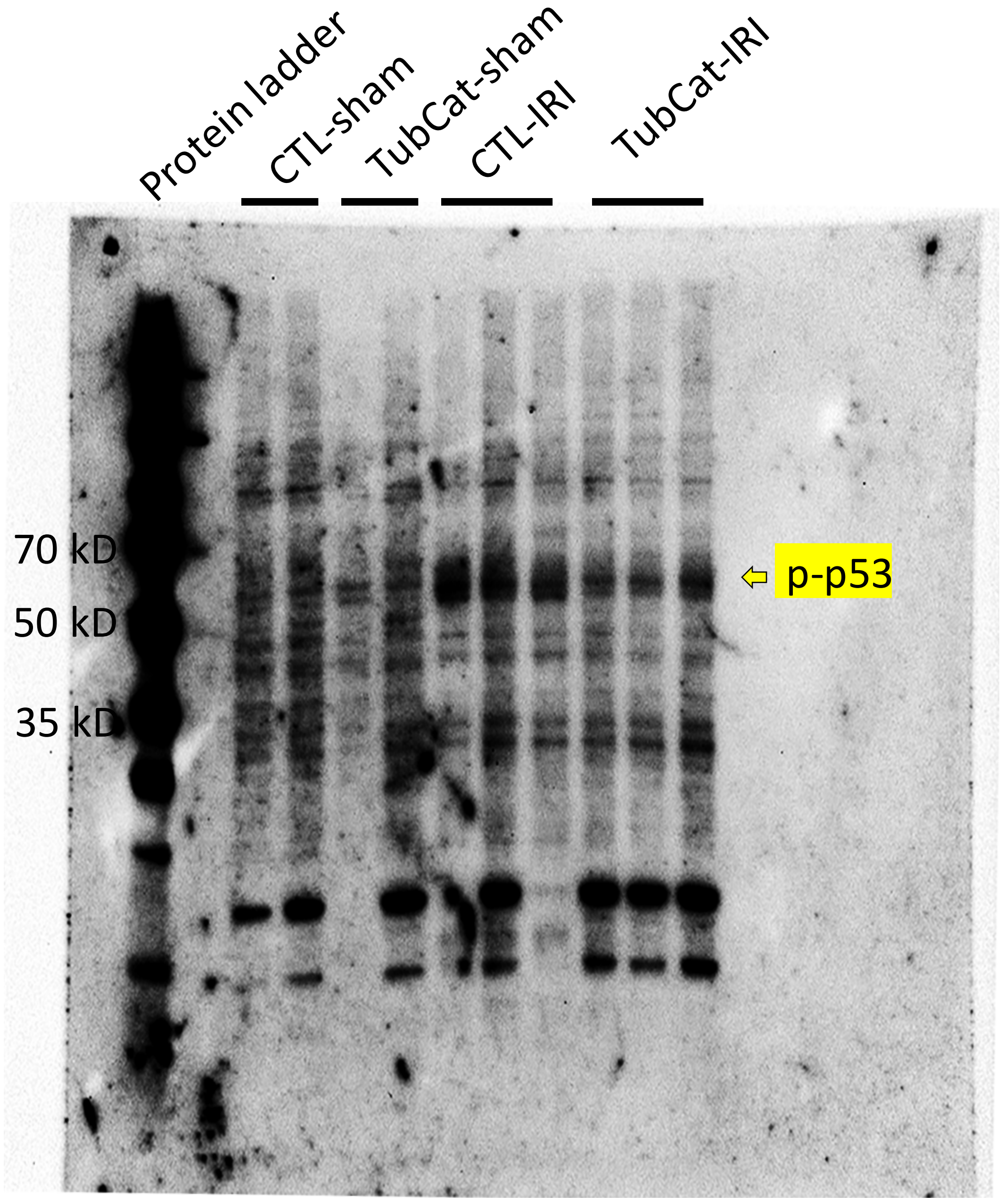

Review files– western blot

12. Fig 3E, β-ACTIN for p-p53

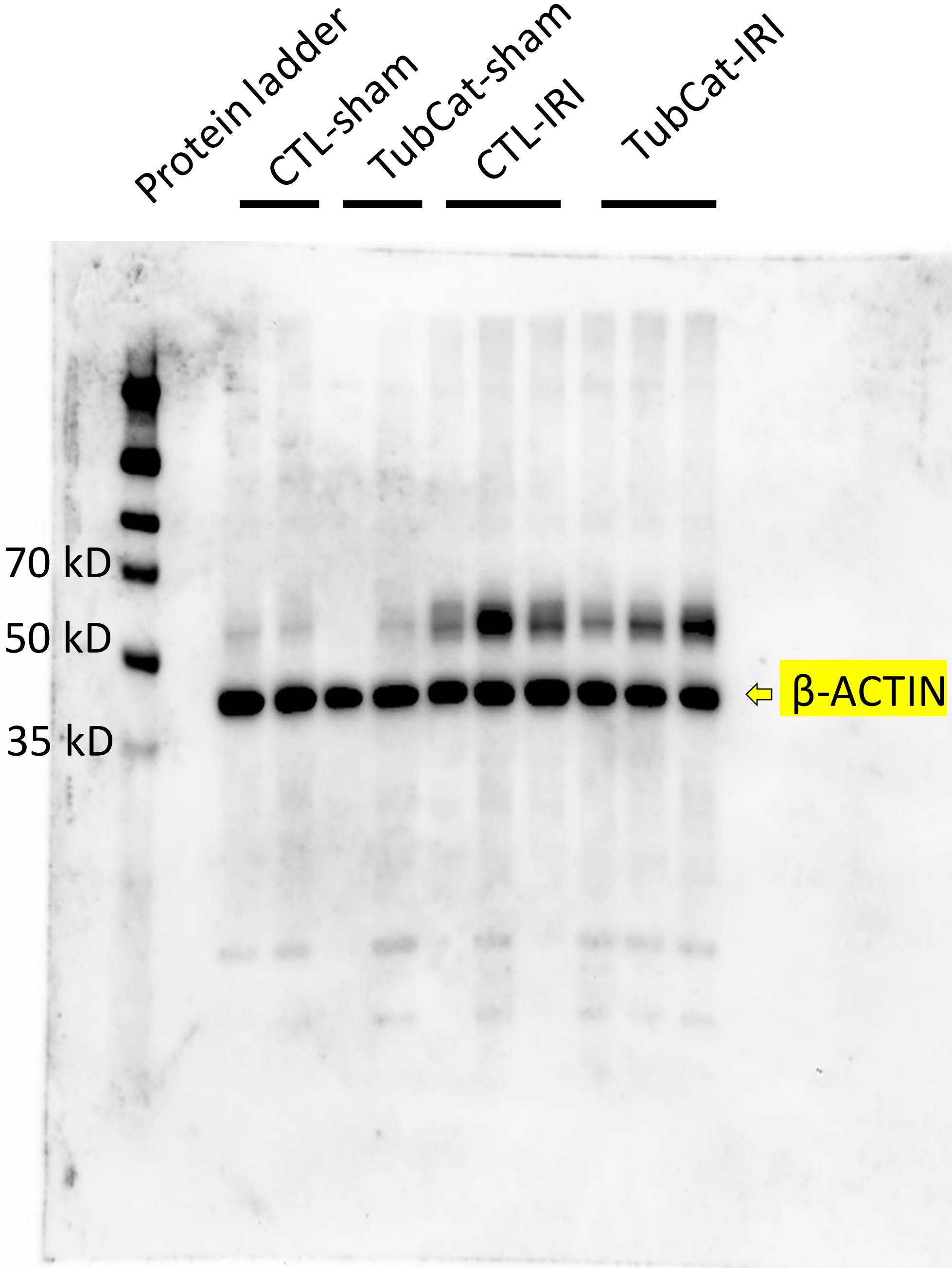

Review files– western blot

13. Fig 3F, p-AKT

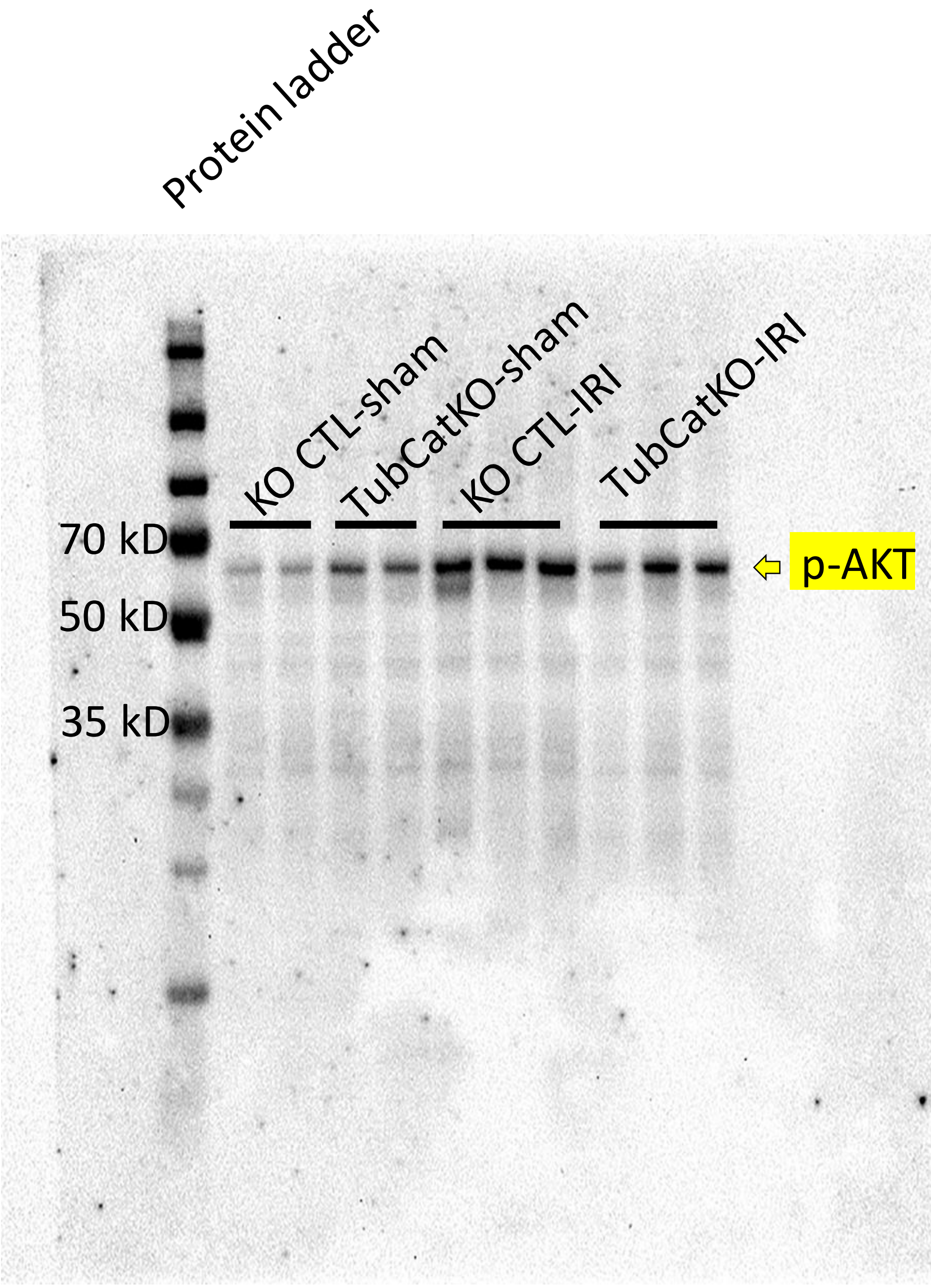

Review files– western blot

14. Fig 3F, AKT

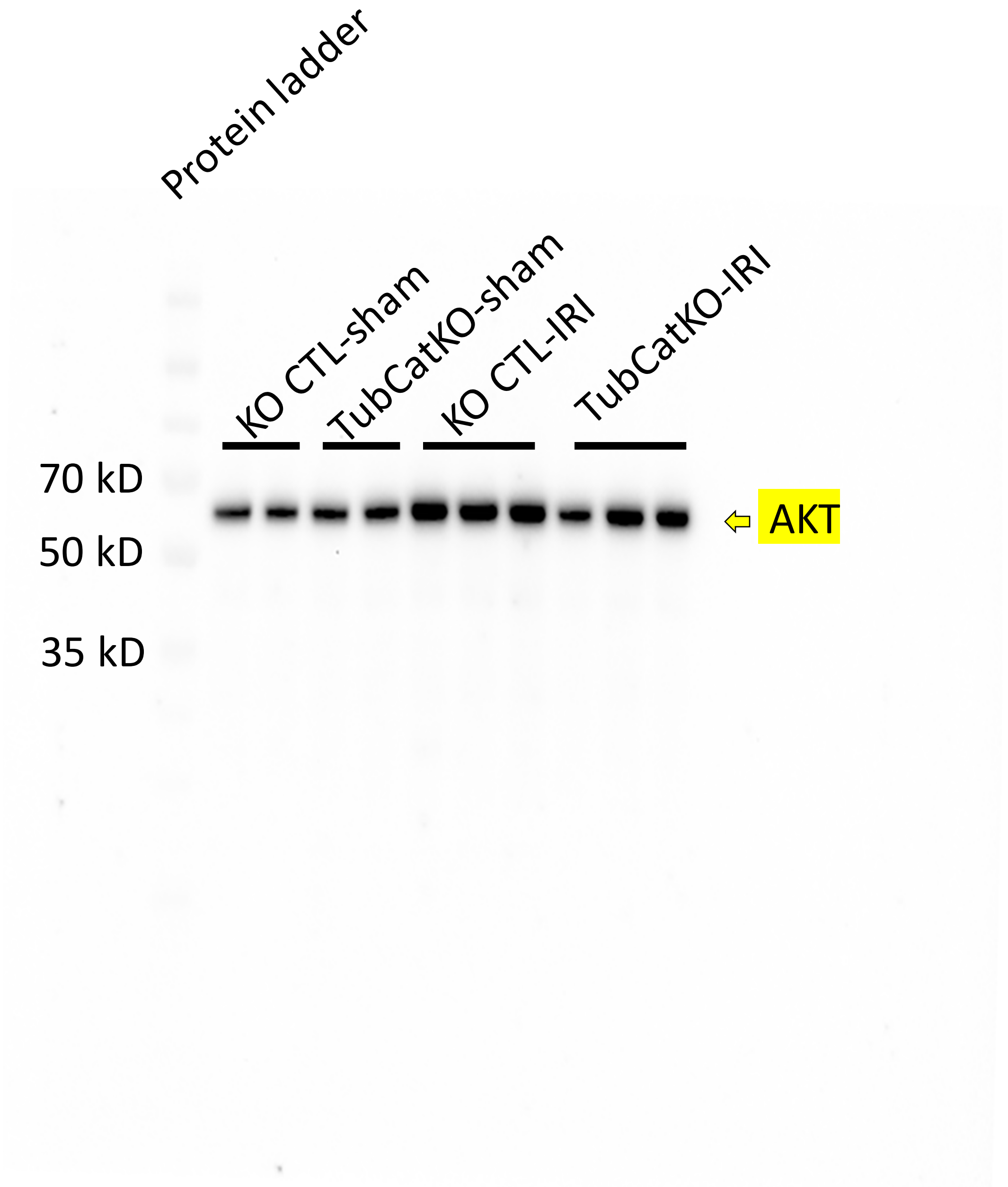

Review files– western blot

15. Fig 3F, p-p53

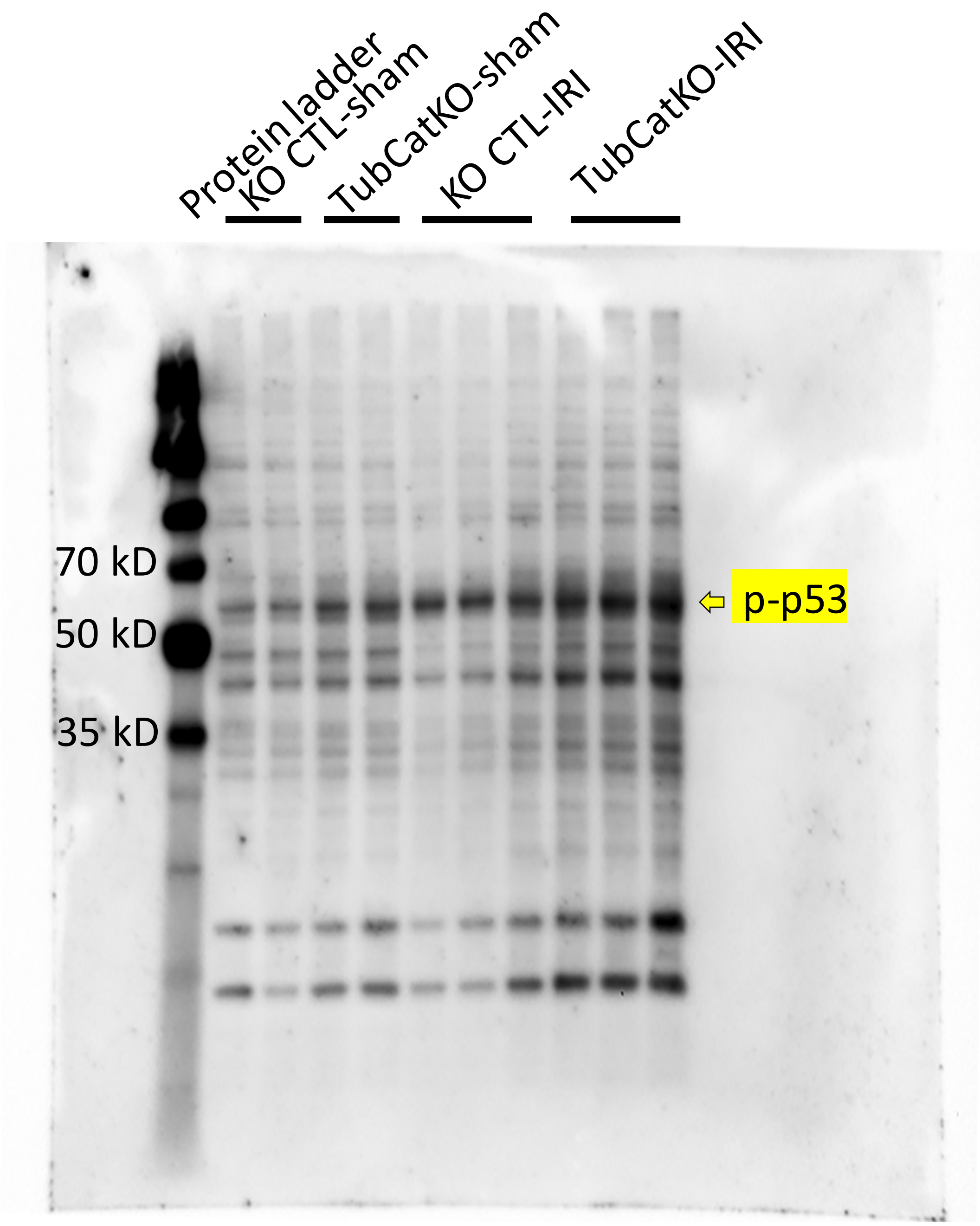

Review files– western blot

16. Fig 3F, β-ACTIN for p-p53

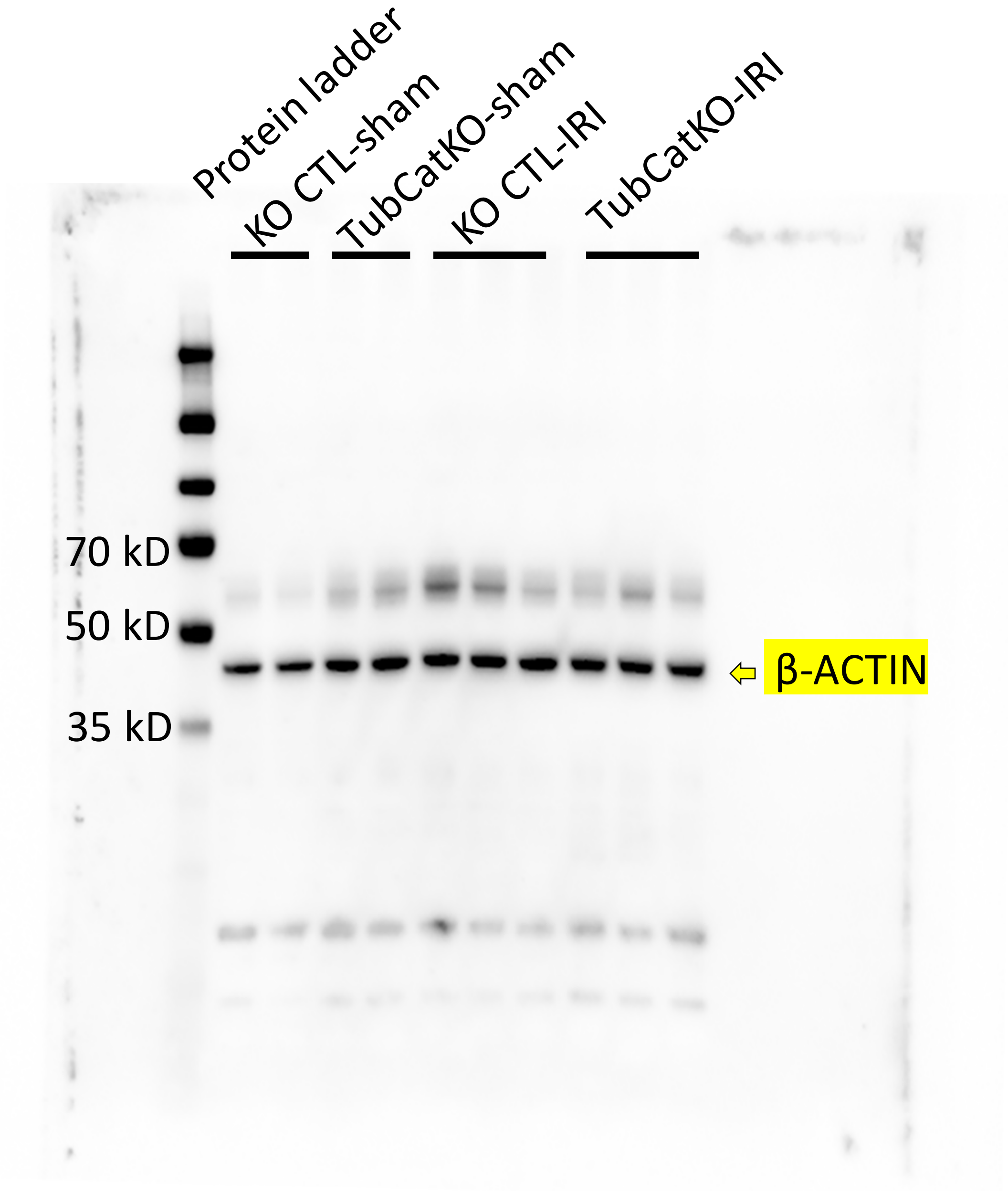

Review files– western blot

17. Fig 4C, p-MLKL

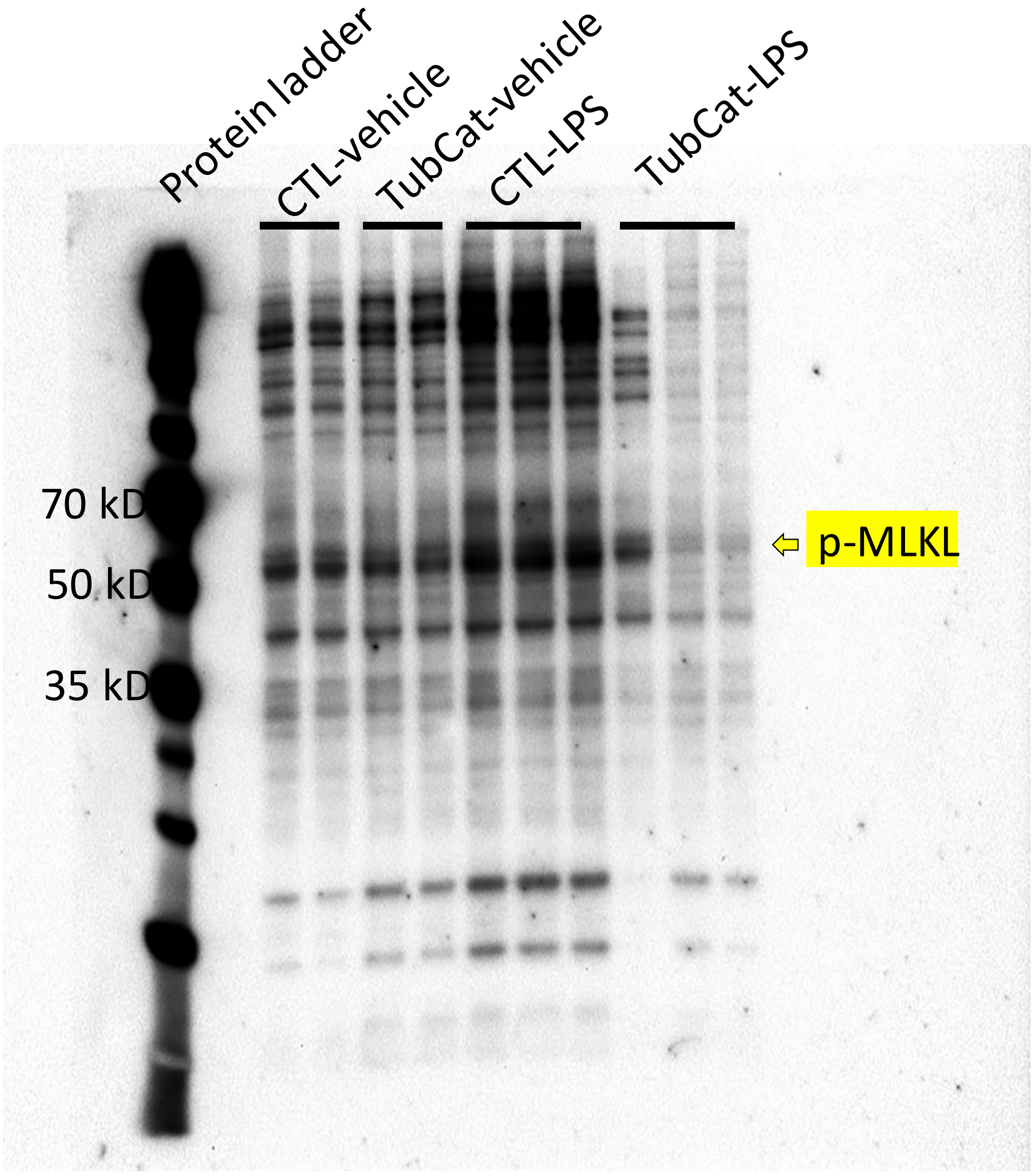

Review files– western blot

18. Fig 4C, β-ACTIN for p-MLKL

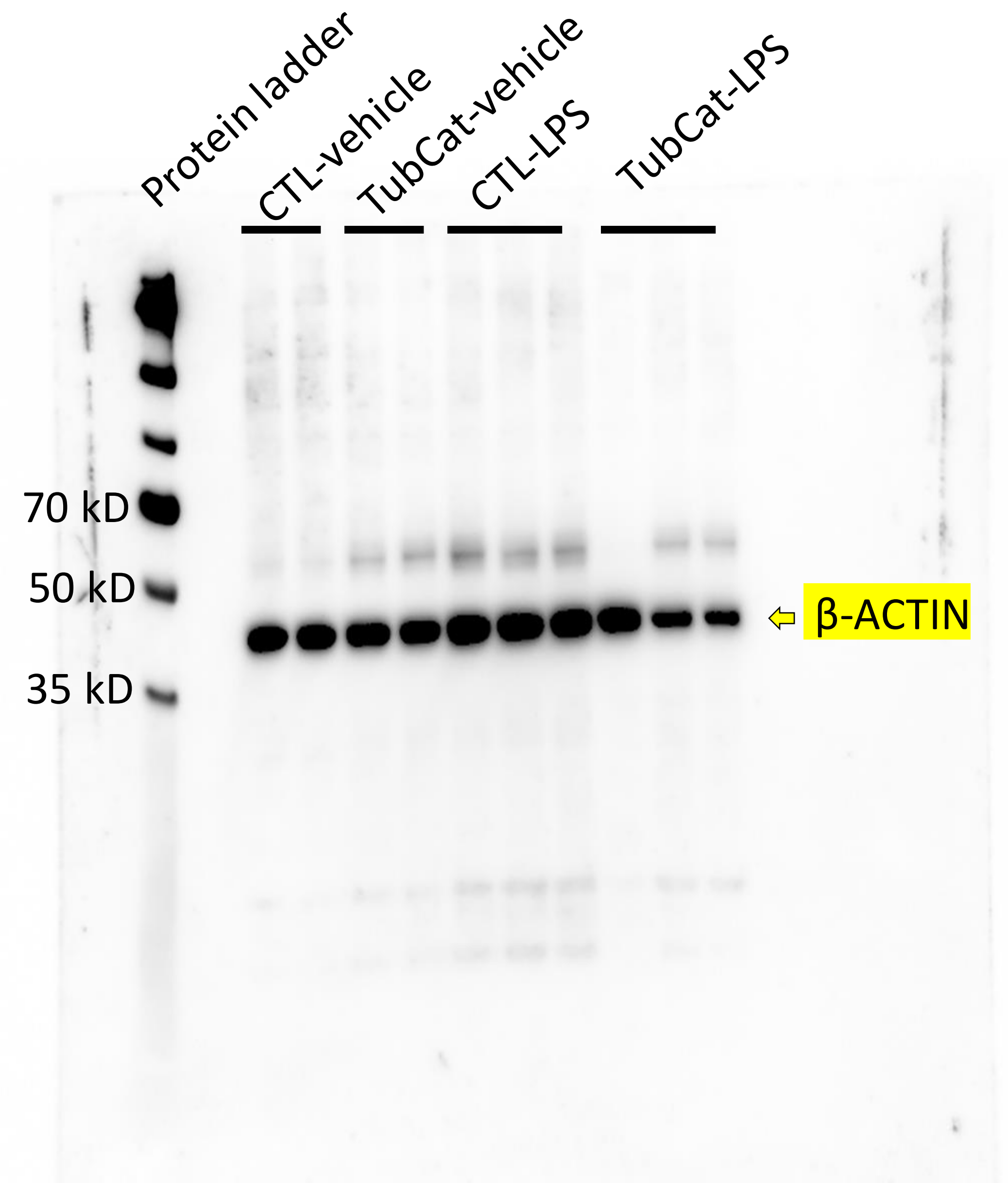

Review files– western blot

19. Fig 4C, p-RIP3

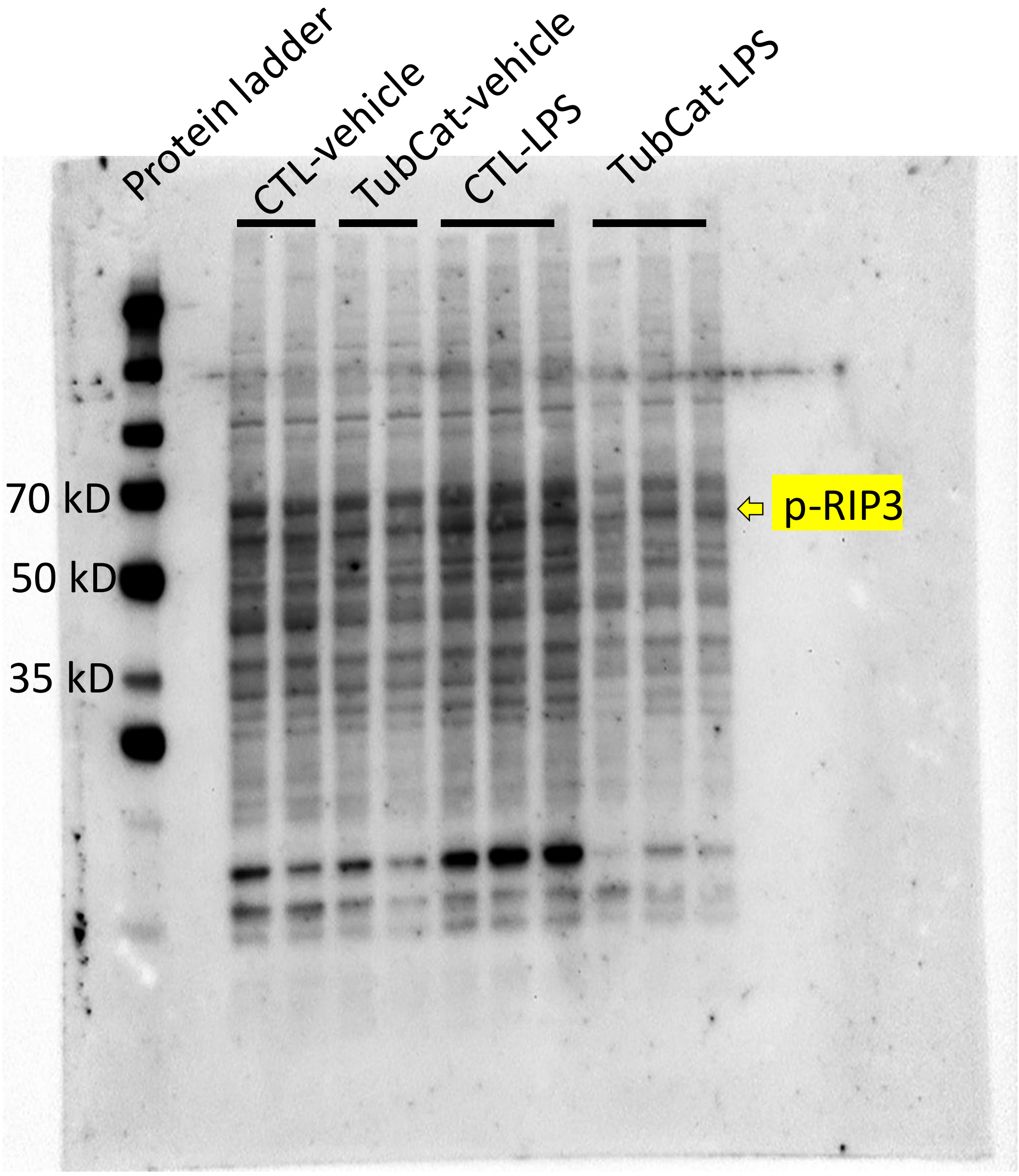

Review files– western blot

20. Fig 4C, β-ACTIN for p-RIP3

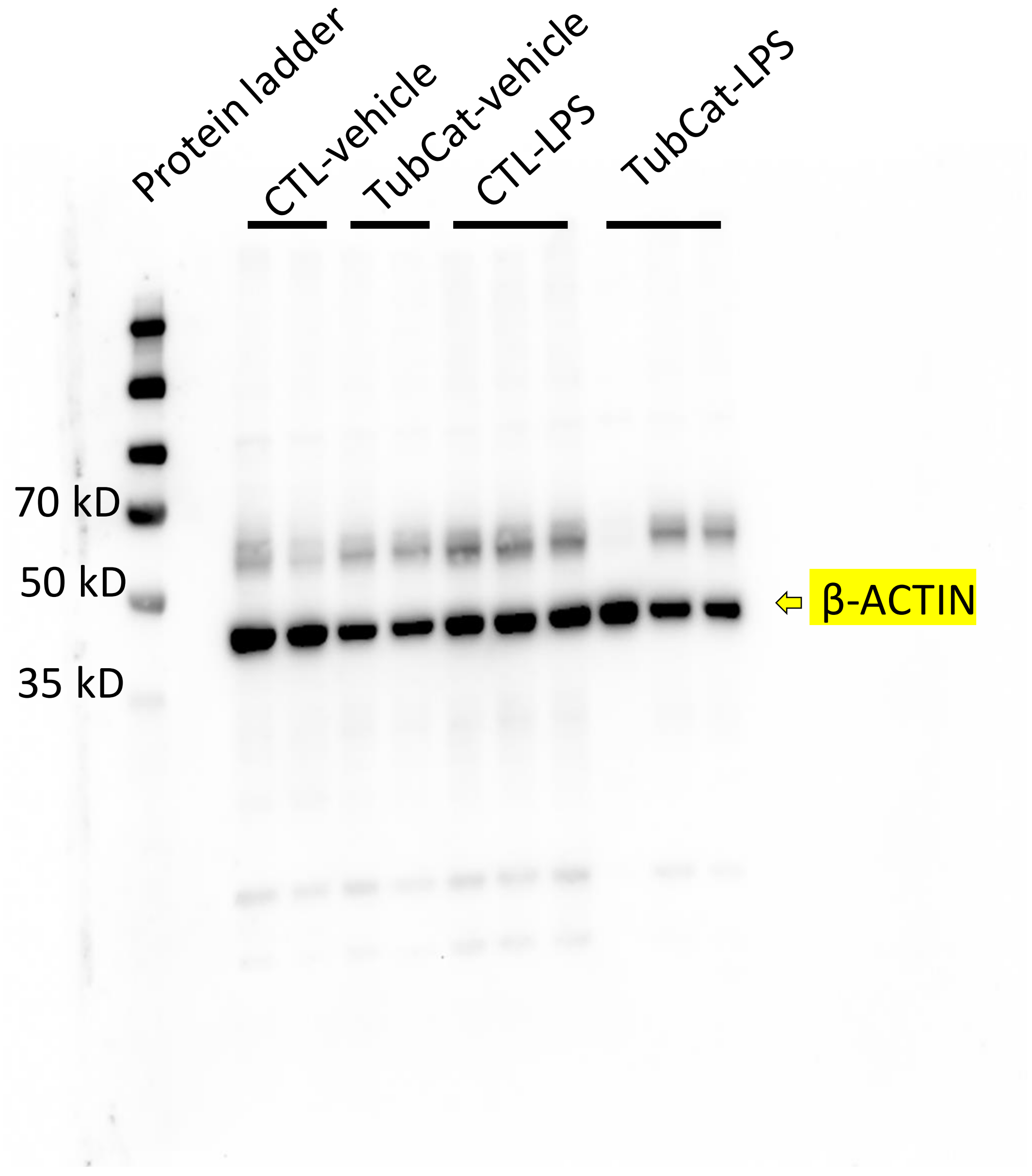

Review files– western blot

21. Fig 4D, p-MLKL

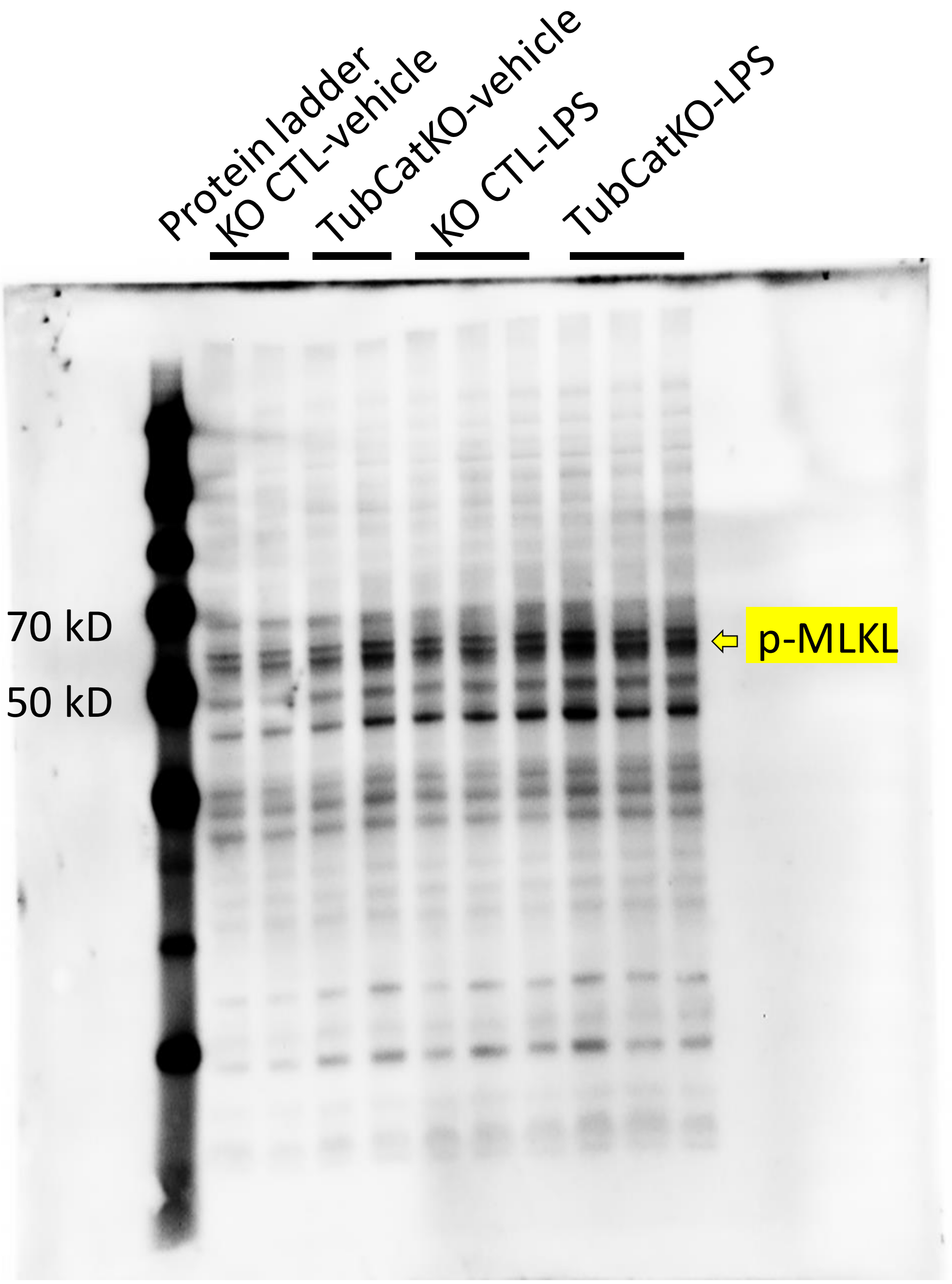

Review files– western blot

22. Fig 4D, β-ACTIN for p-MLKL

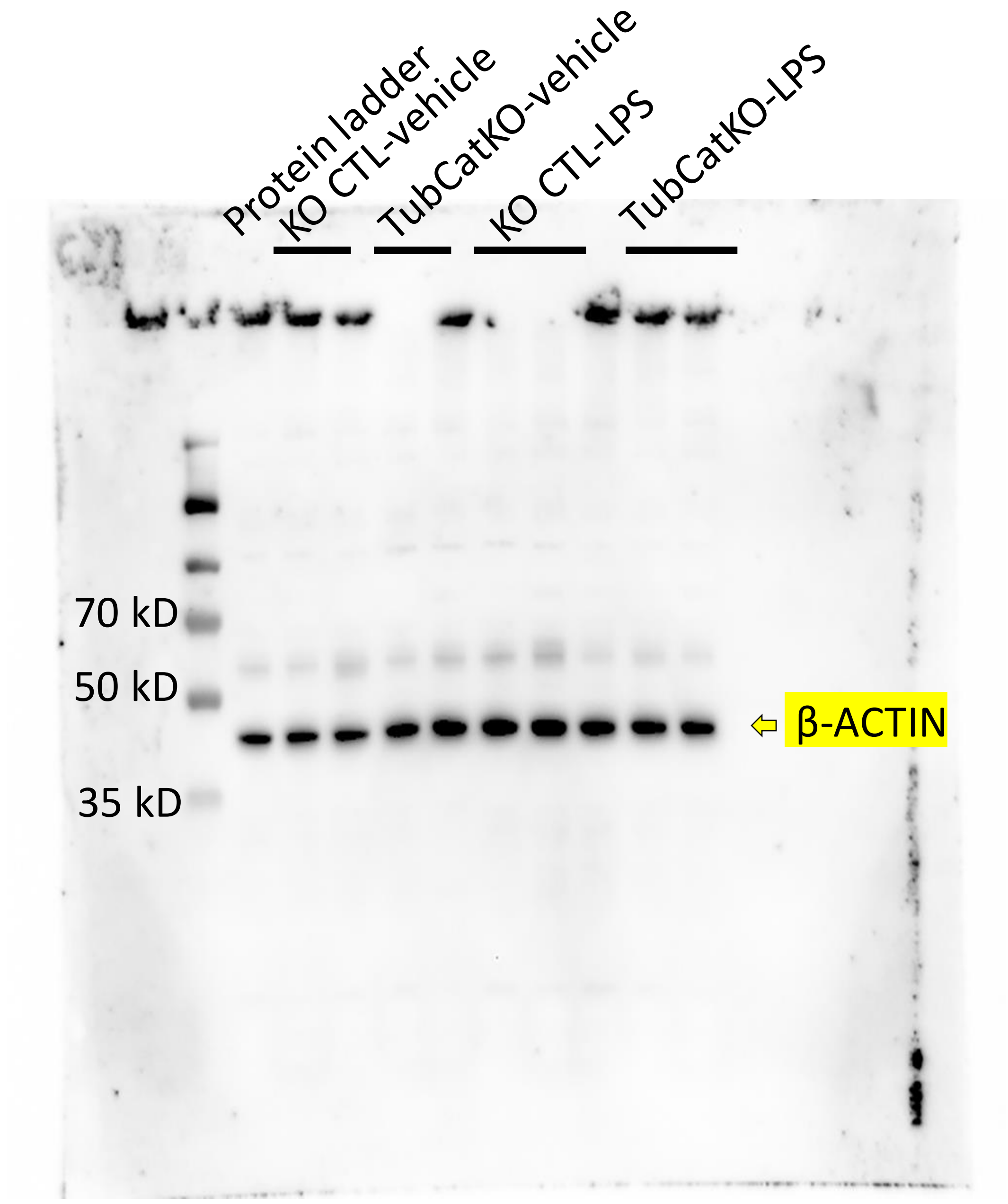

Review files– western blot

23. Fig 4D, p-RIP3

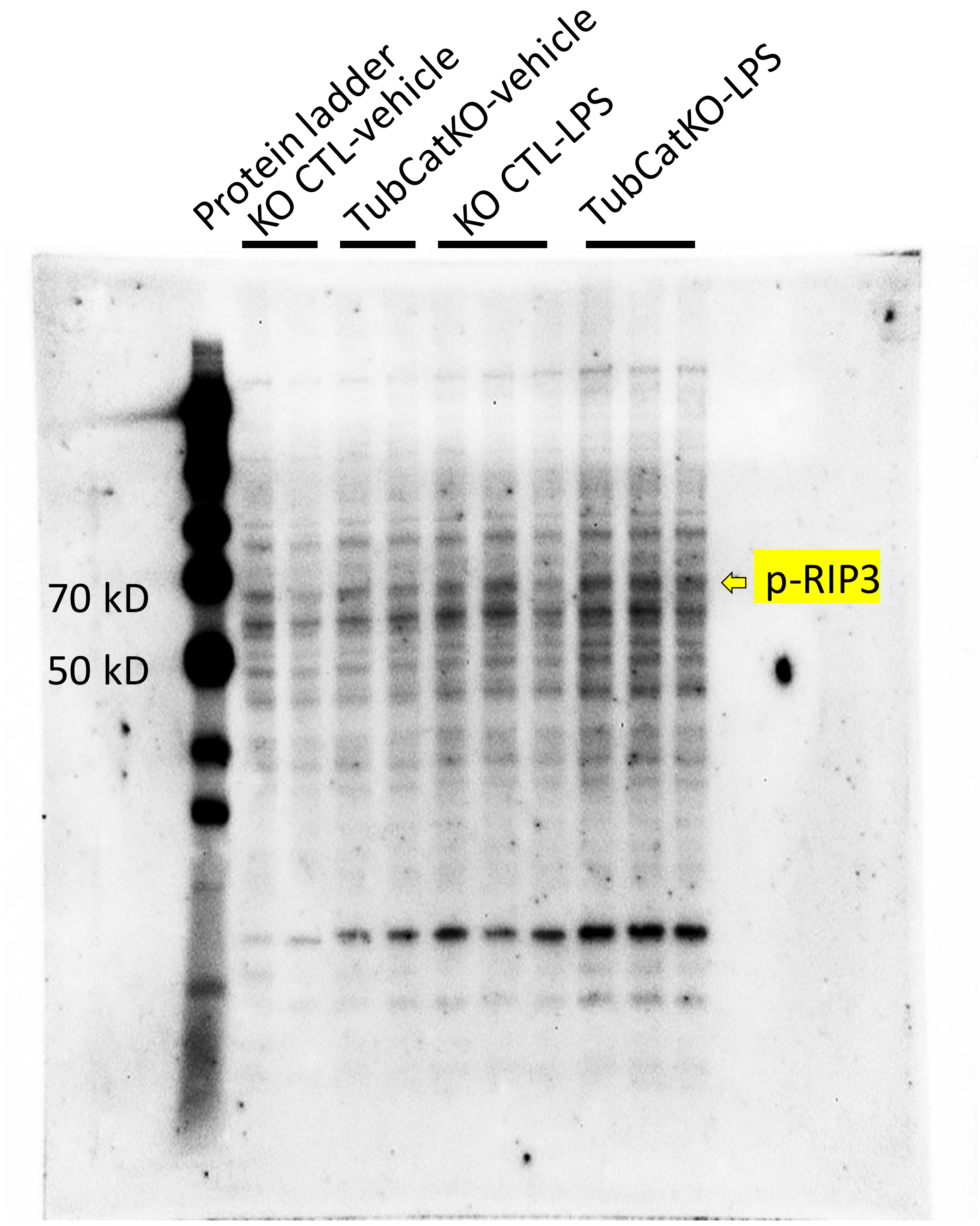

Review files– western blot

24. Fig 4D, β-ACTIN for p-RIP3

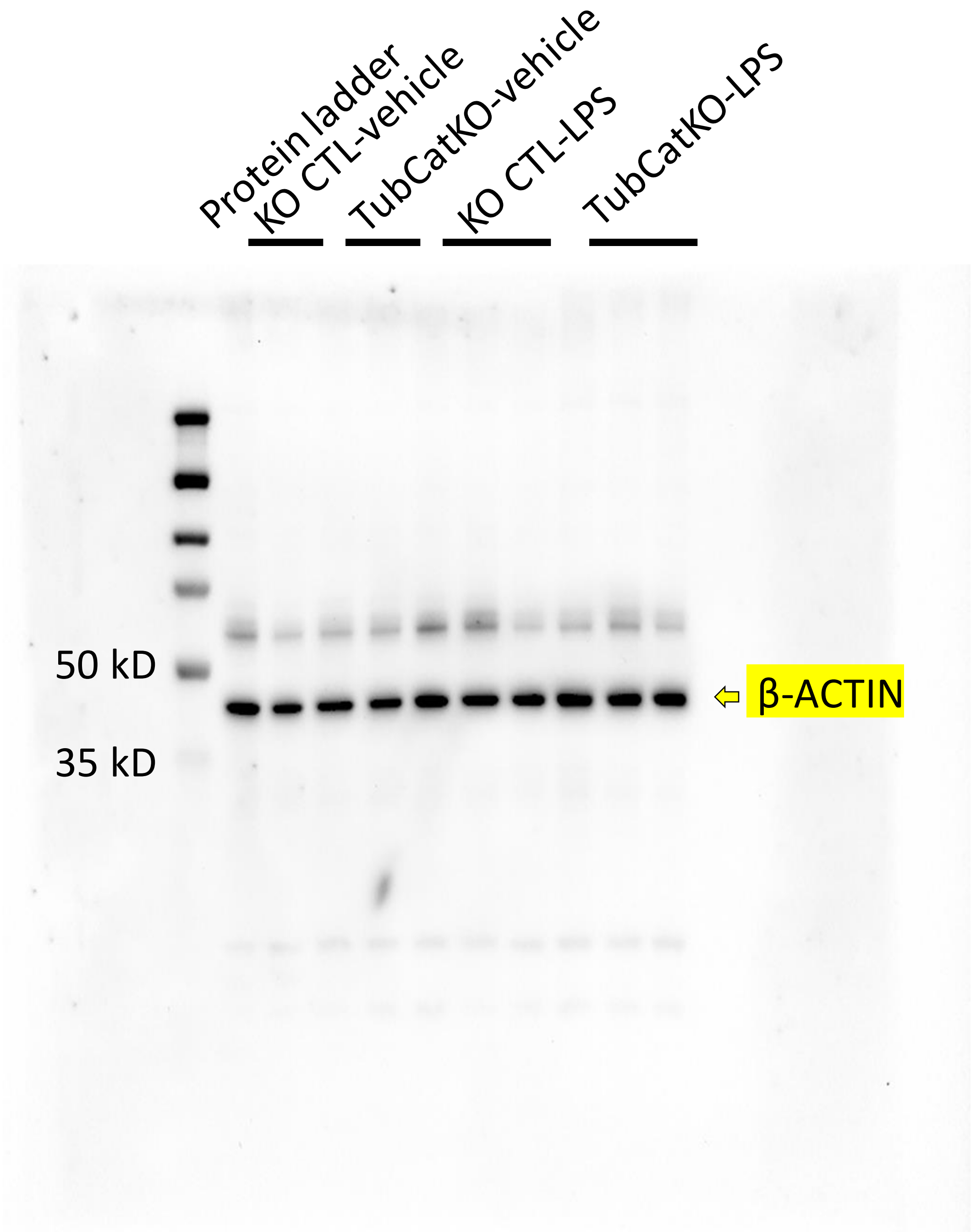

Review files– western blot

25. Fig 4E, p-AKT

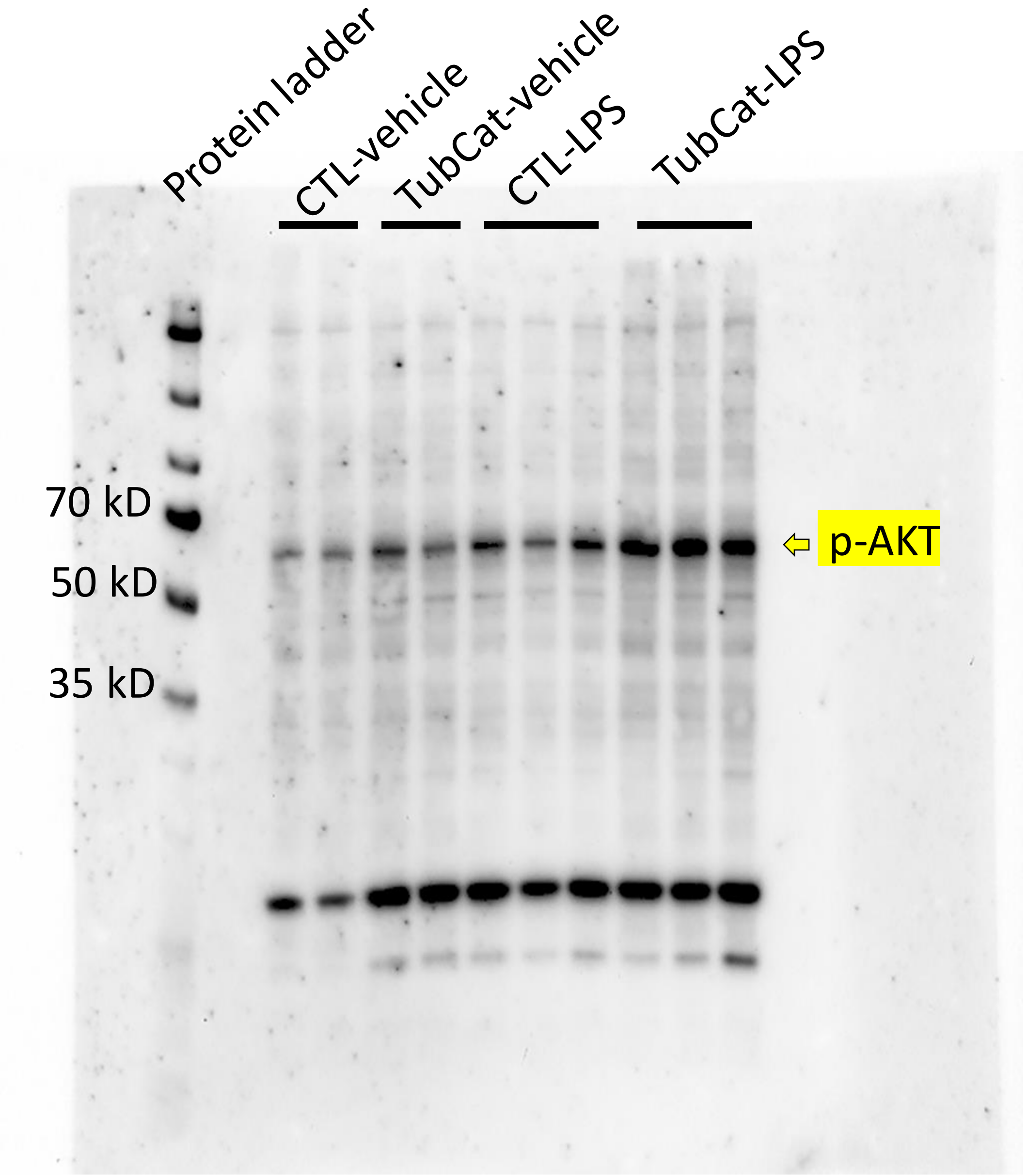

Review files– western blot

26. Fig 4E, AKT

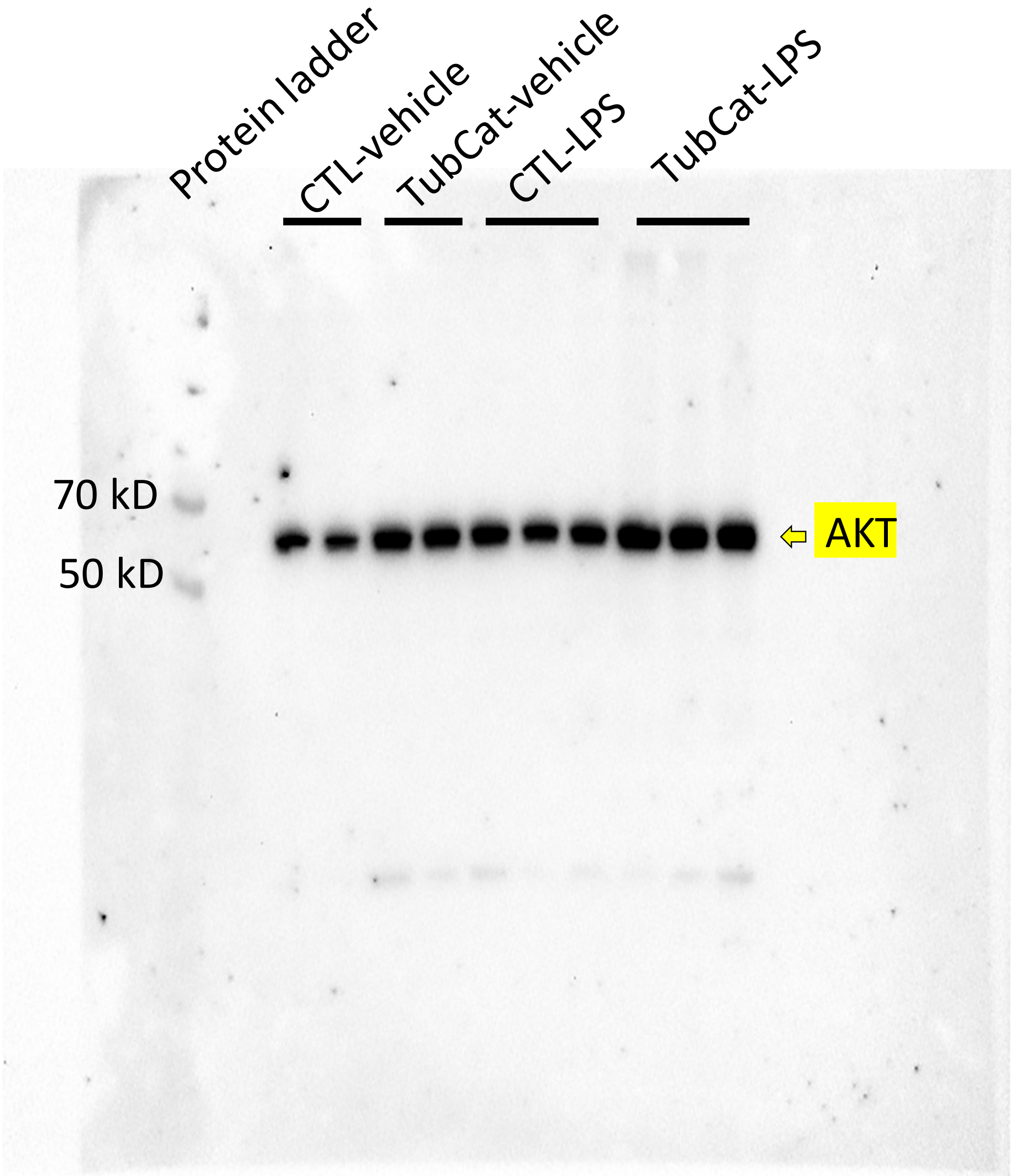

Review files– western blot

27. Fig 4E, p-p53

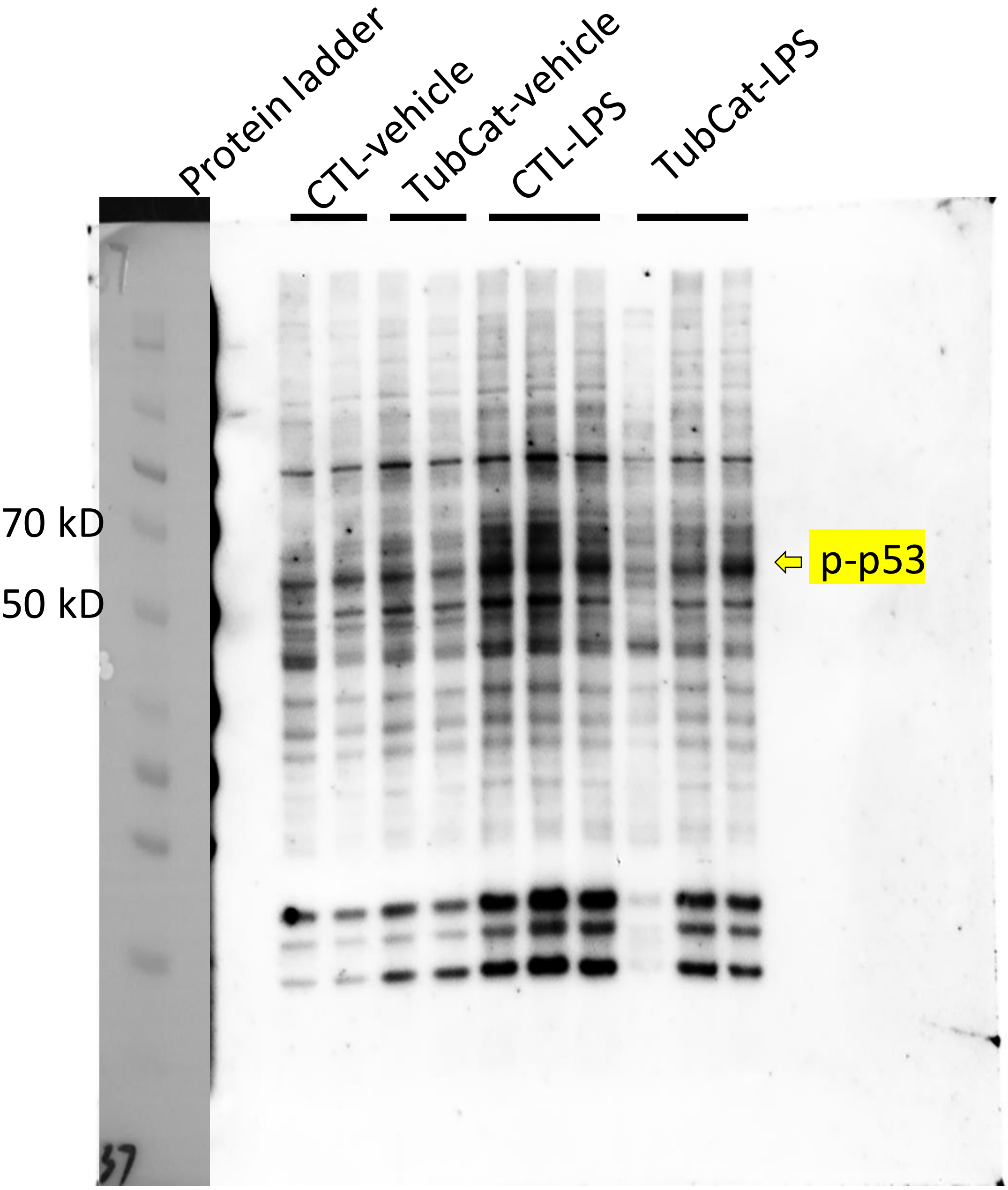

Review files– western blot

28. Fig 4E, β-ACTIN for p-p53

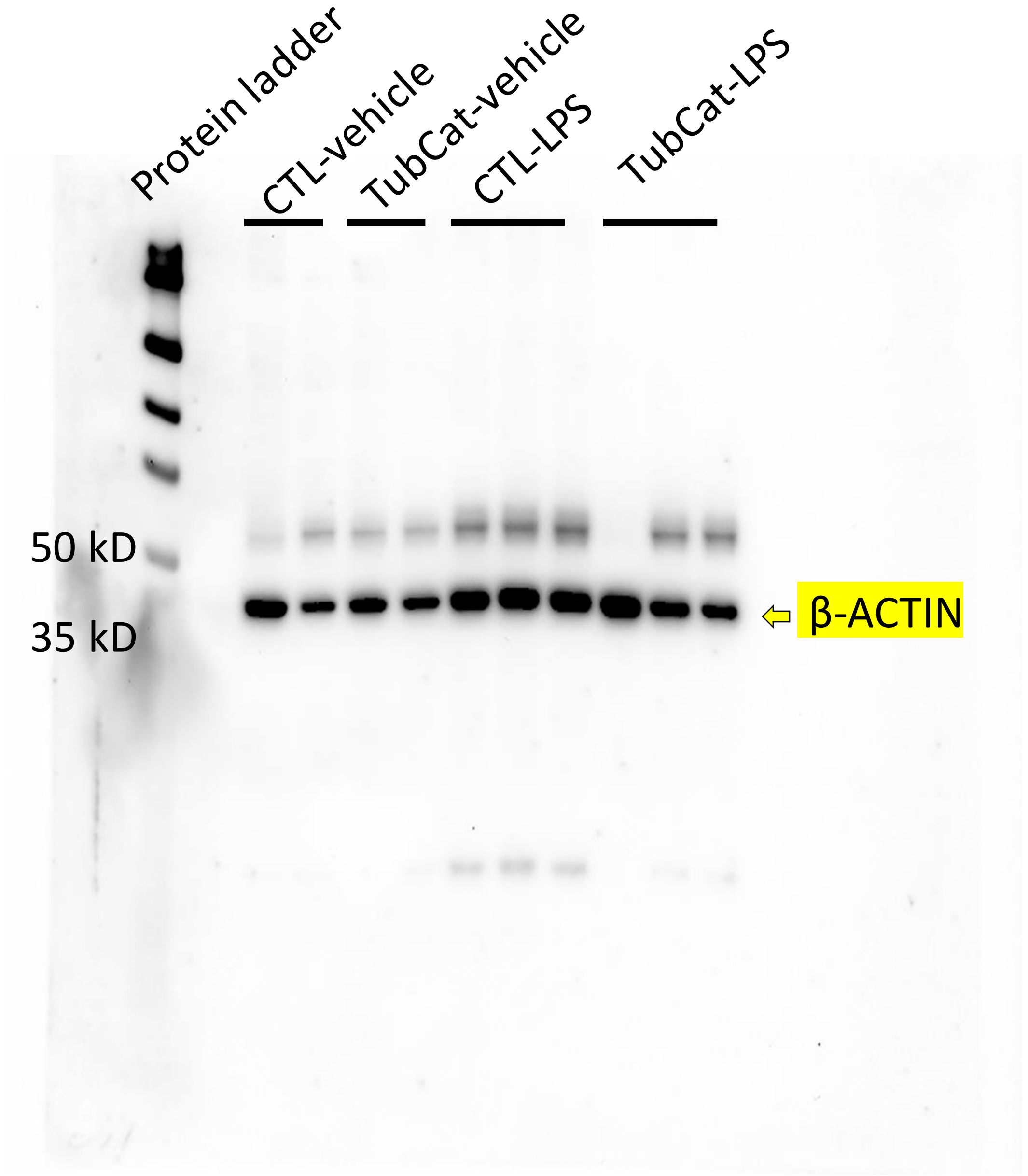

Review files– western blot

29. Fig 4F, p-AKT

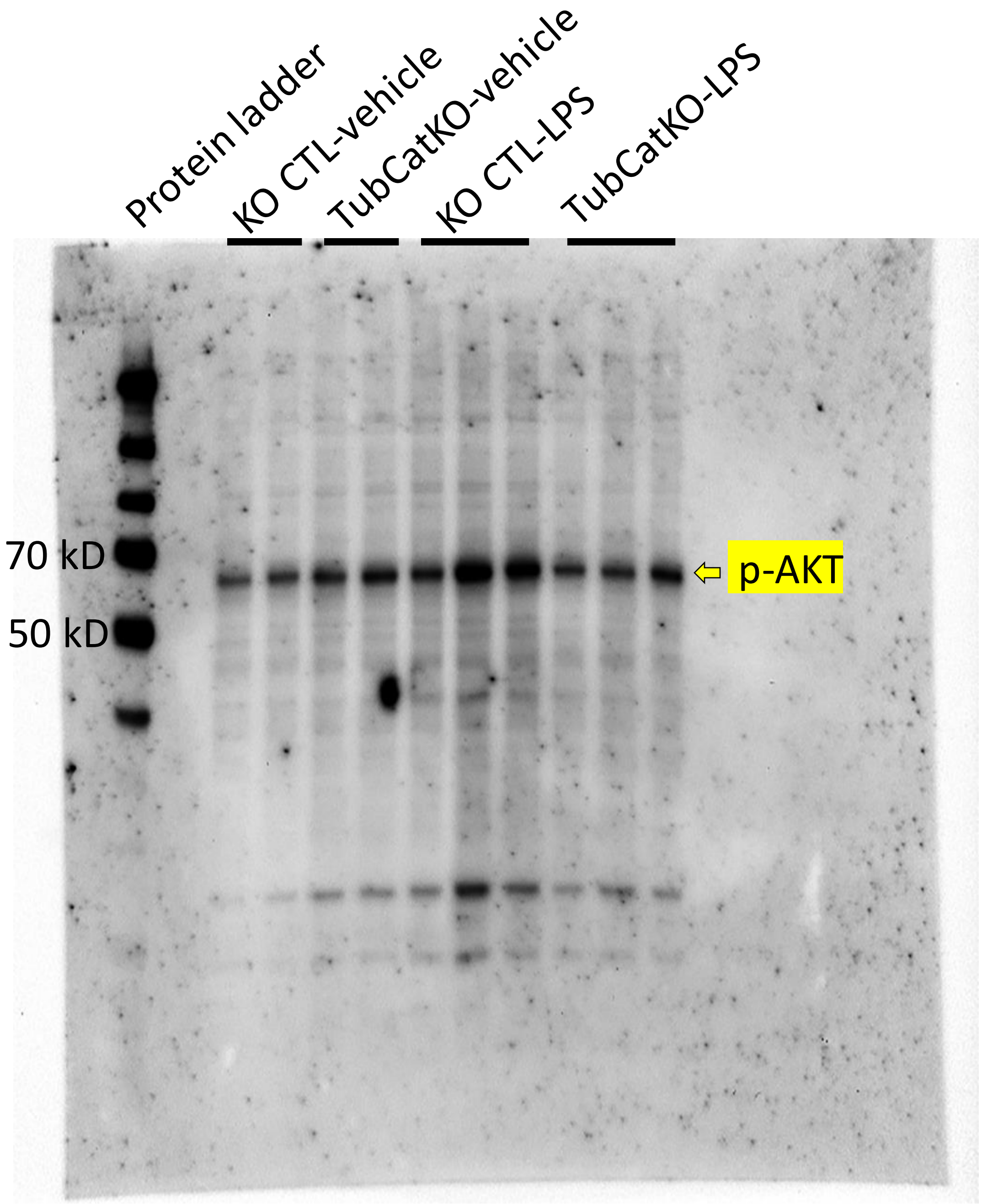

Review files– western blot

30. Fig 4F, AKT

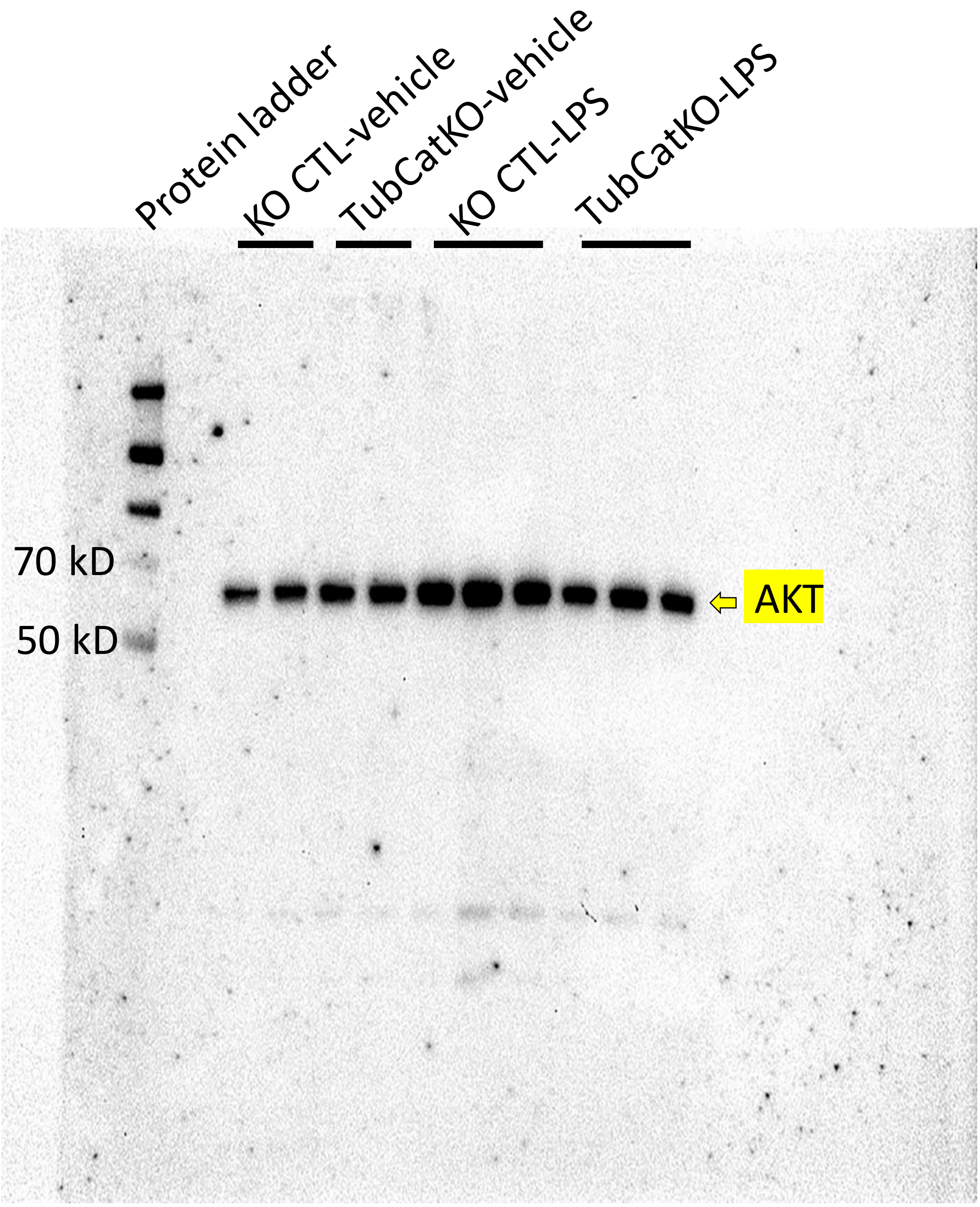

Review files– western blot

31. Fig 4F, p-p53

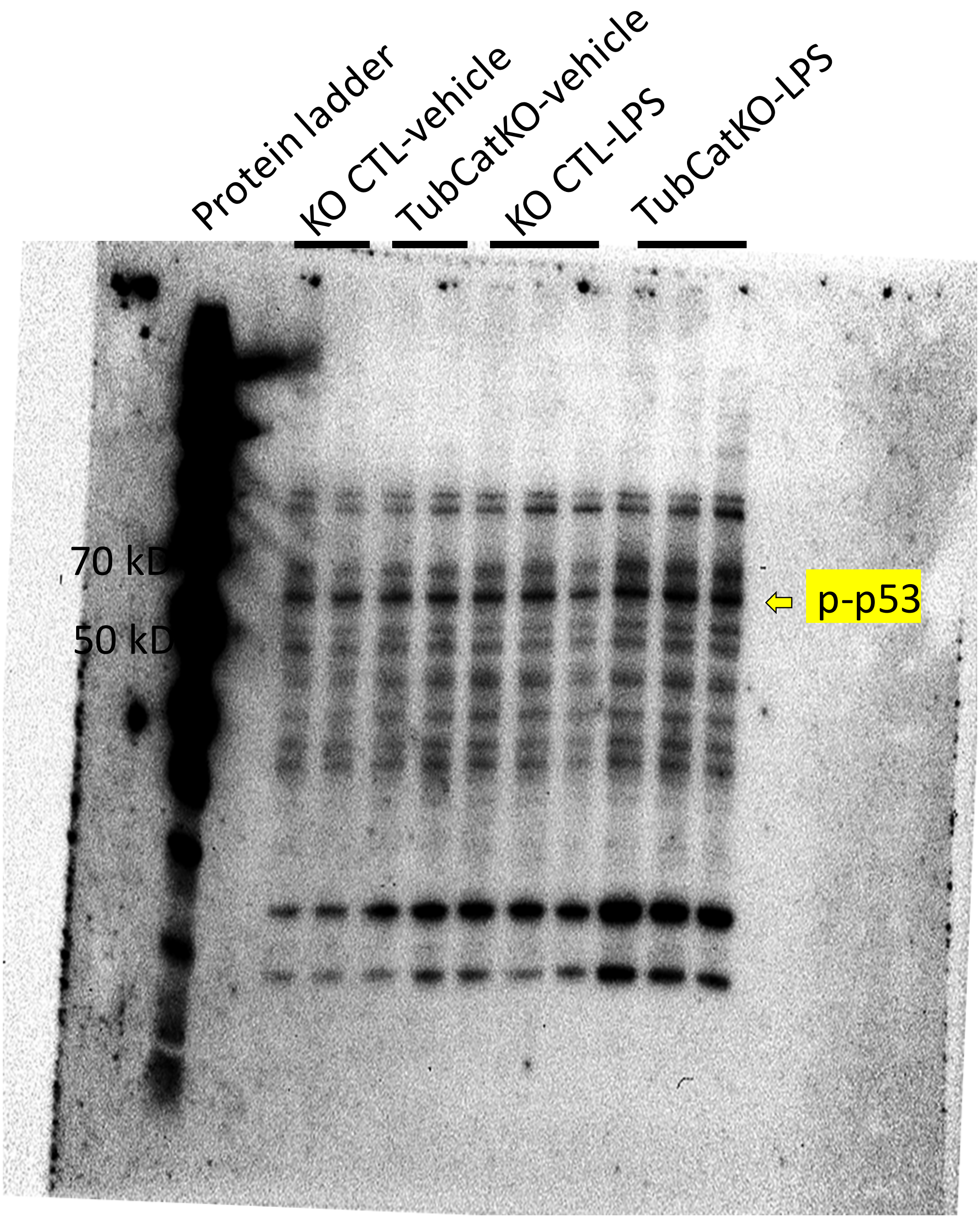

Review files– western blot

32. Fig 4F, β-ACTIN for p-p53

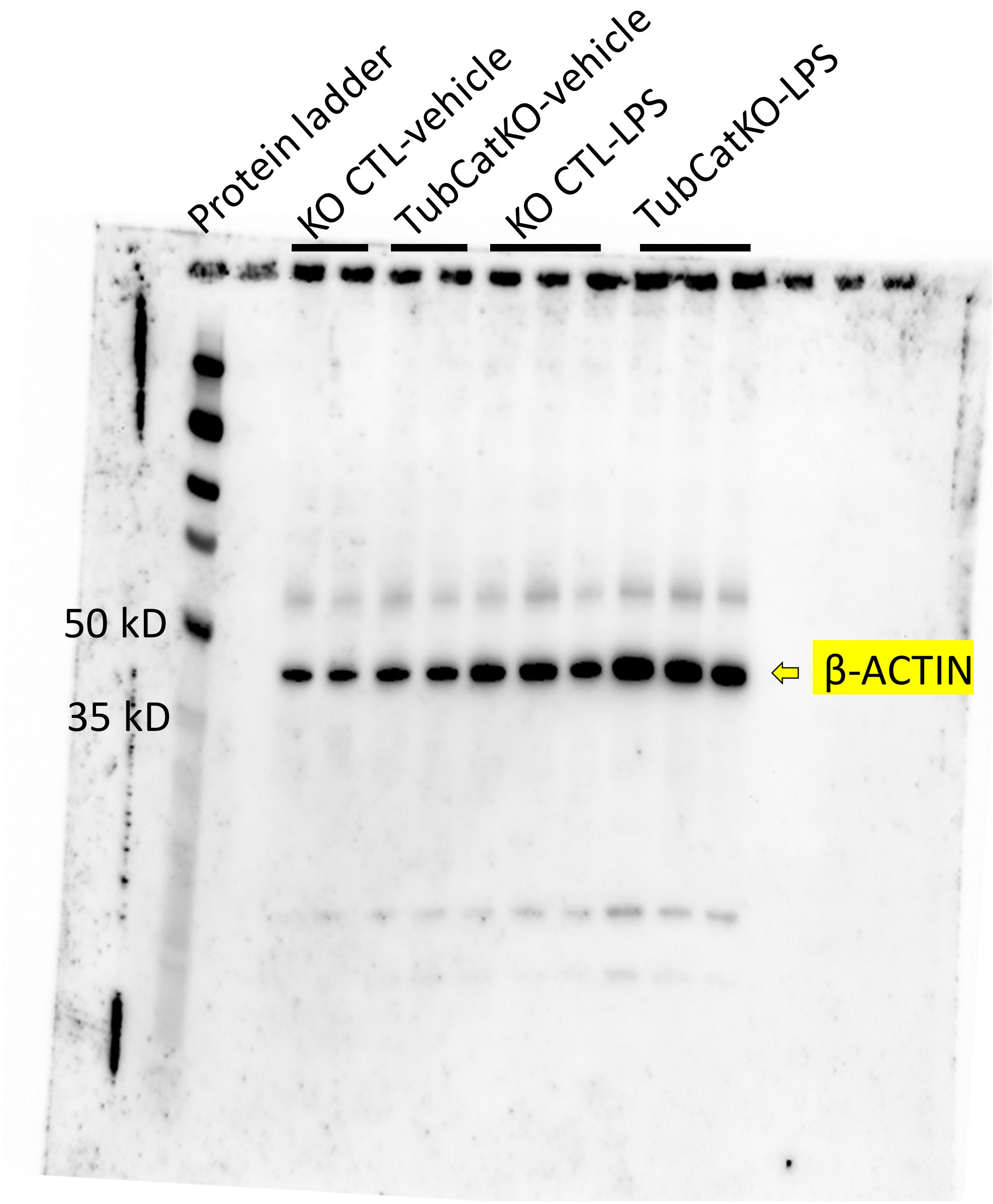

Review files– western blot

33. Fig 5C, PGC-1α

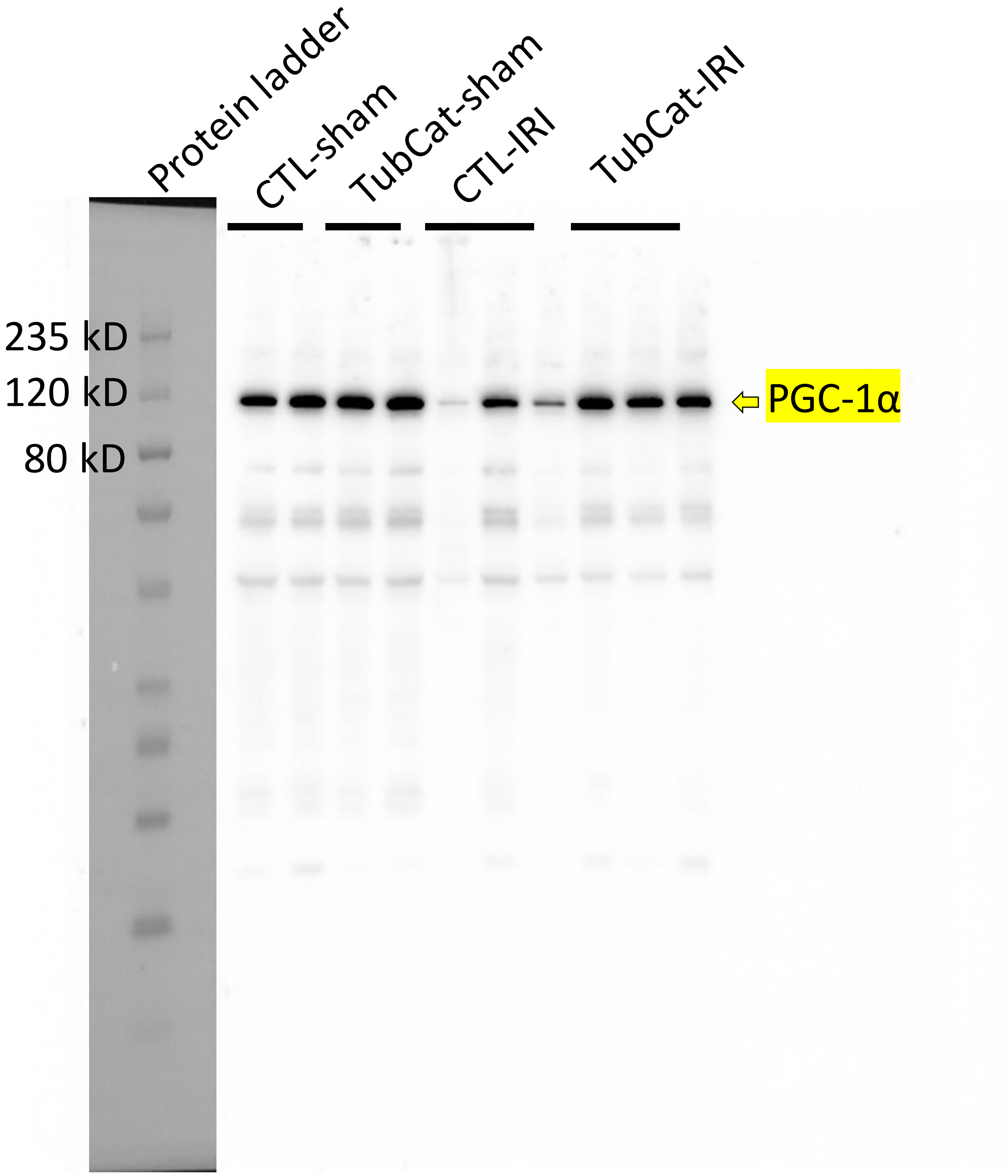

Review files– western blot

34. Fig 5C, β-ACTIN for PGC-1α, the representative β-ACTIN band chosen showed in Fig 5C

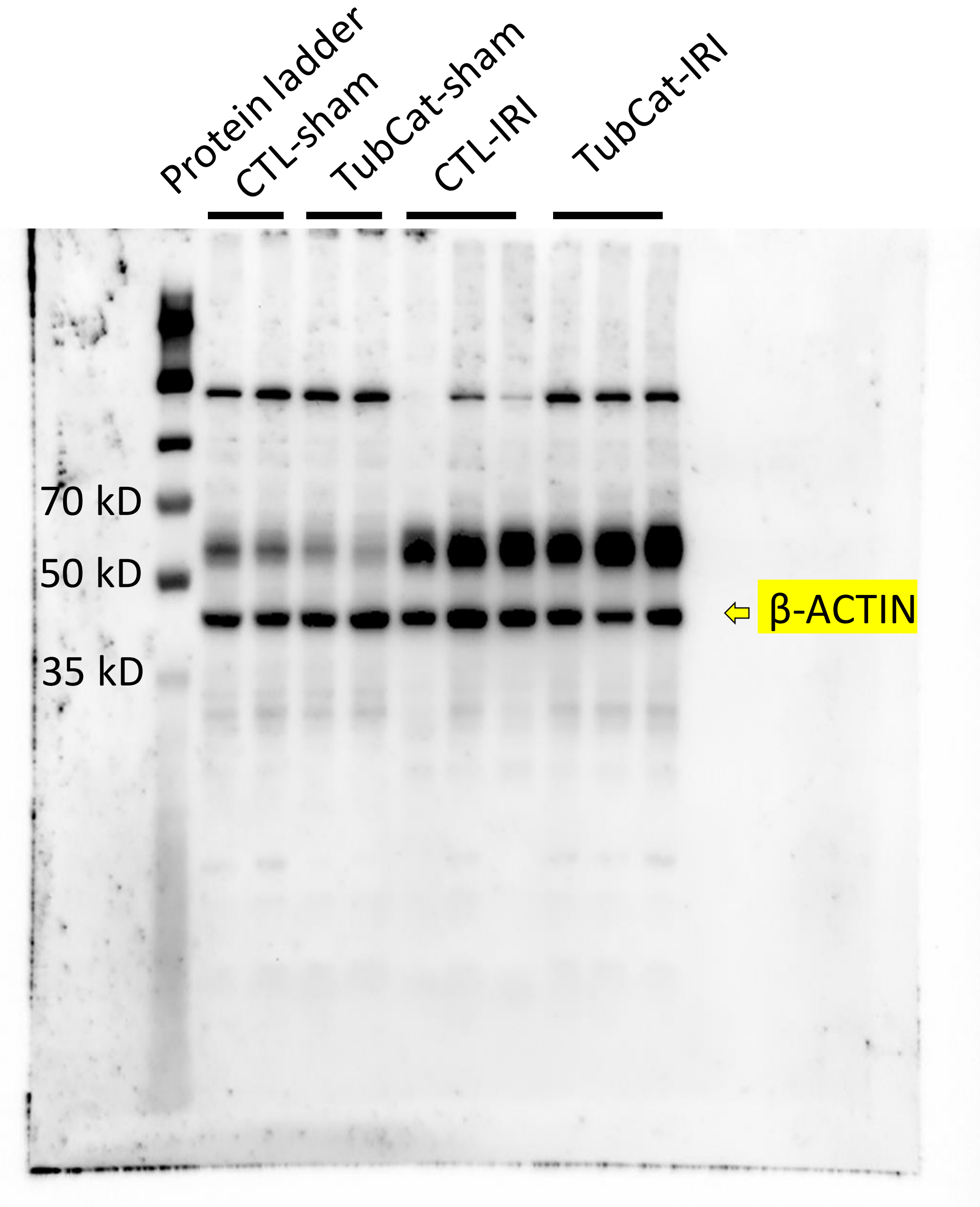

Review files– western blot

35. Fig 5C, NRF1

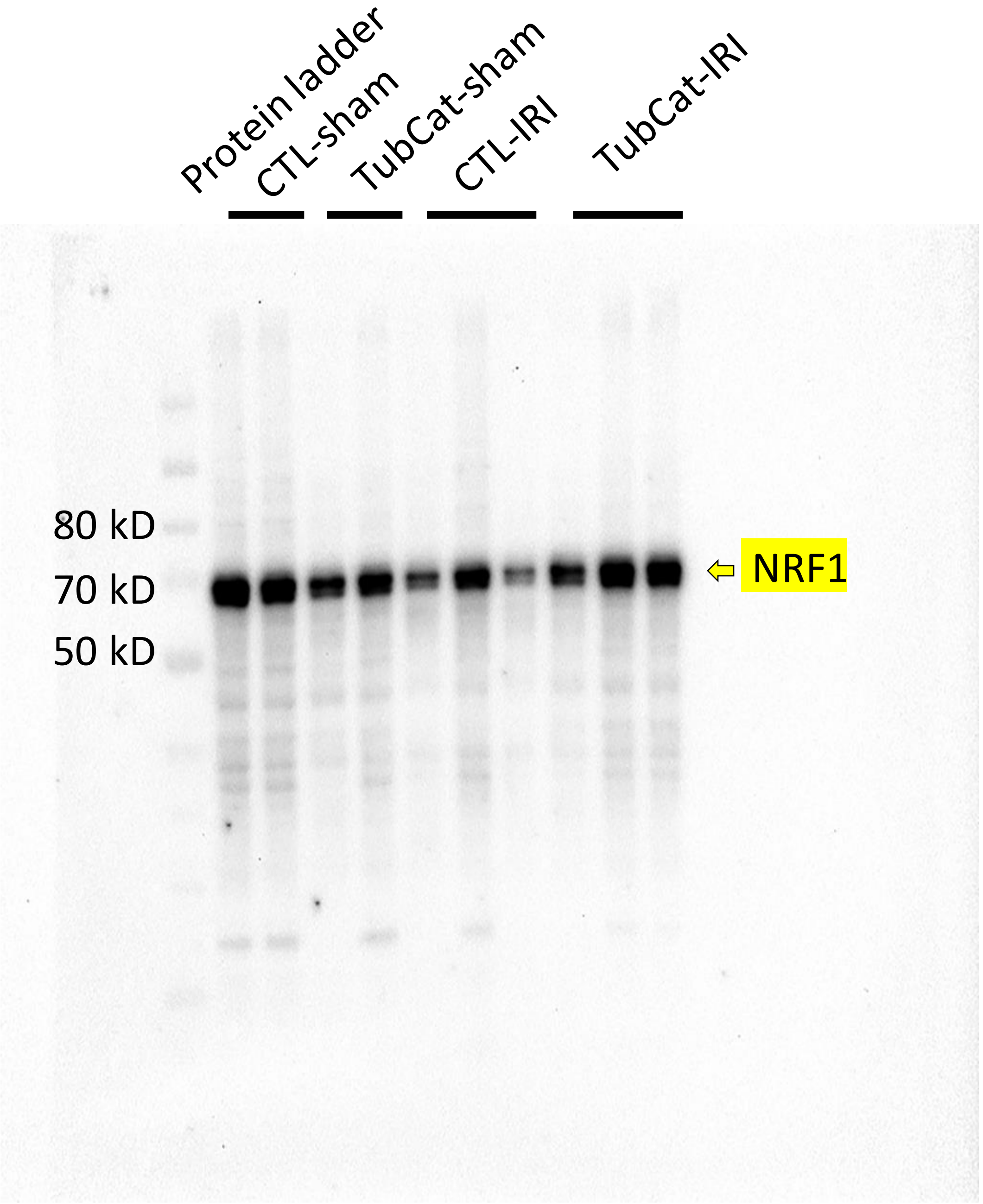

Review files– western blot

36. Fig 5C, β-ACTIN for NRF1

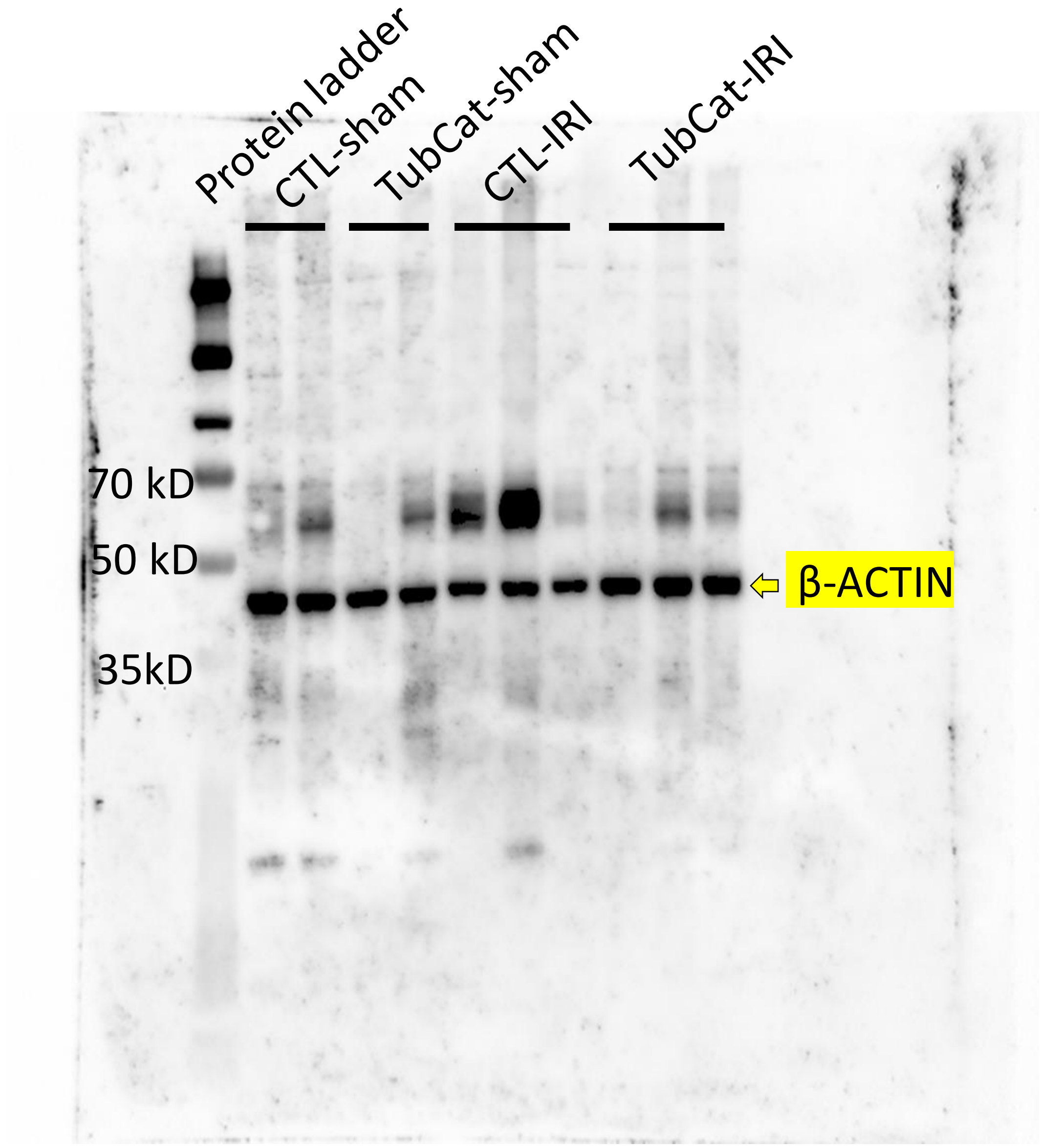

Review files– western blot

37. Fig 5C, TIM23

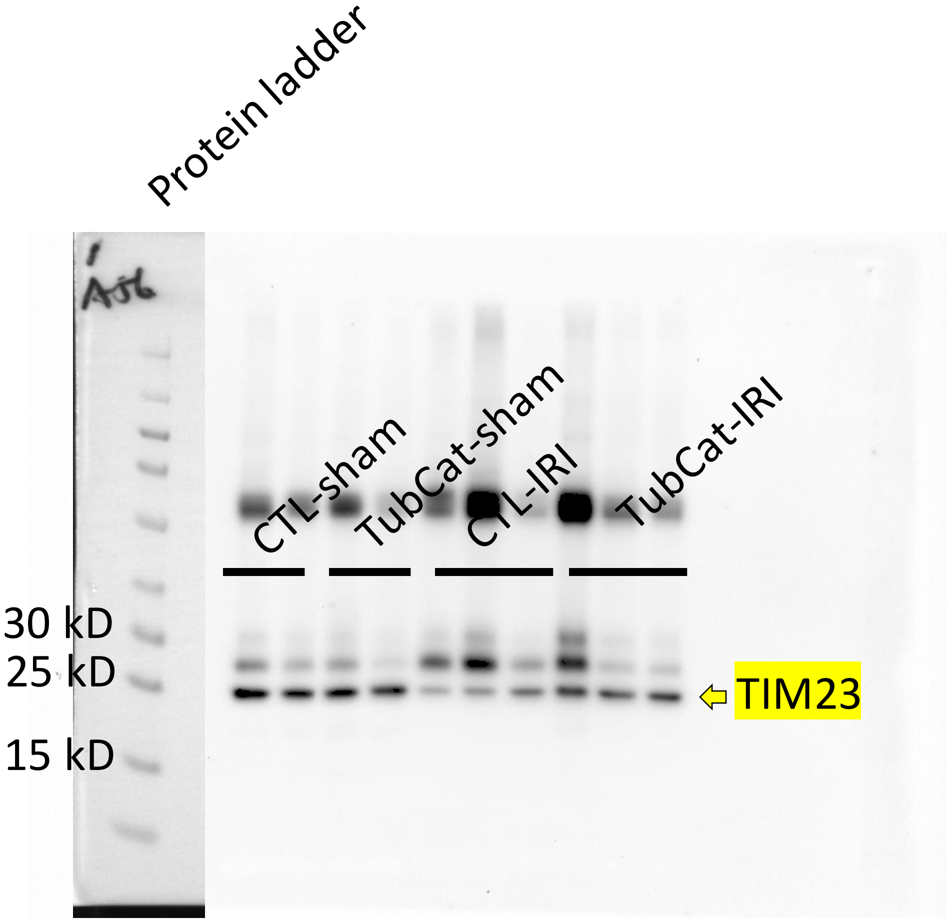

Review files– western blot

38. Fig 5C, β-ACTIN for TIM23

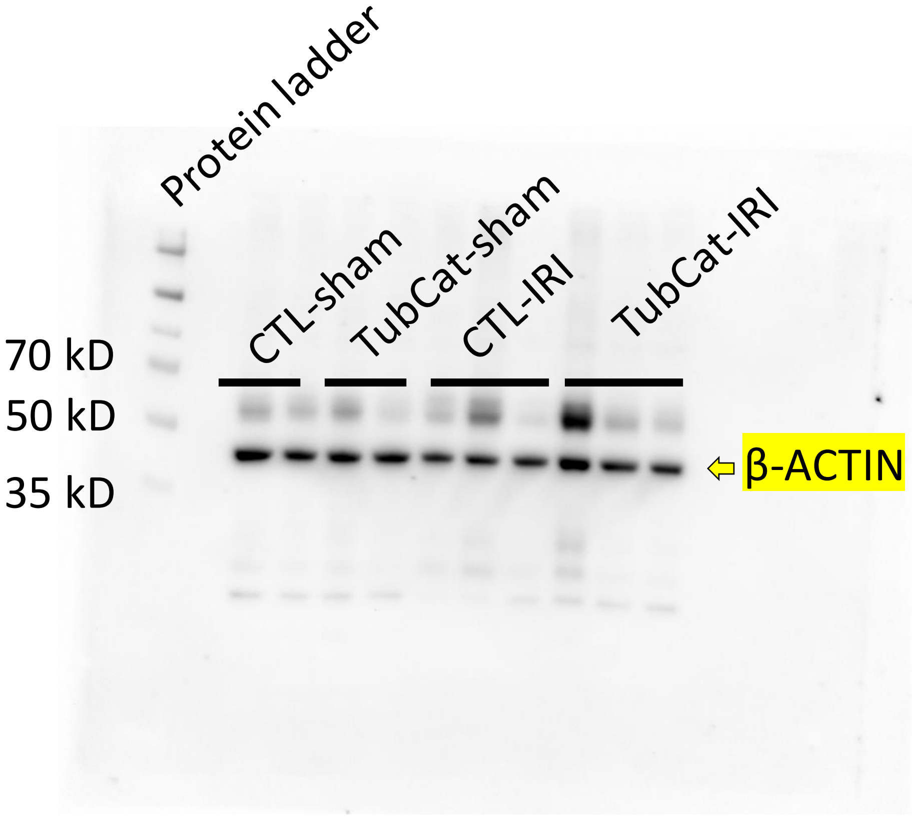

Review files– western blot

39. Fig 5D, PGC-1α

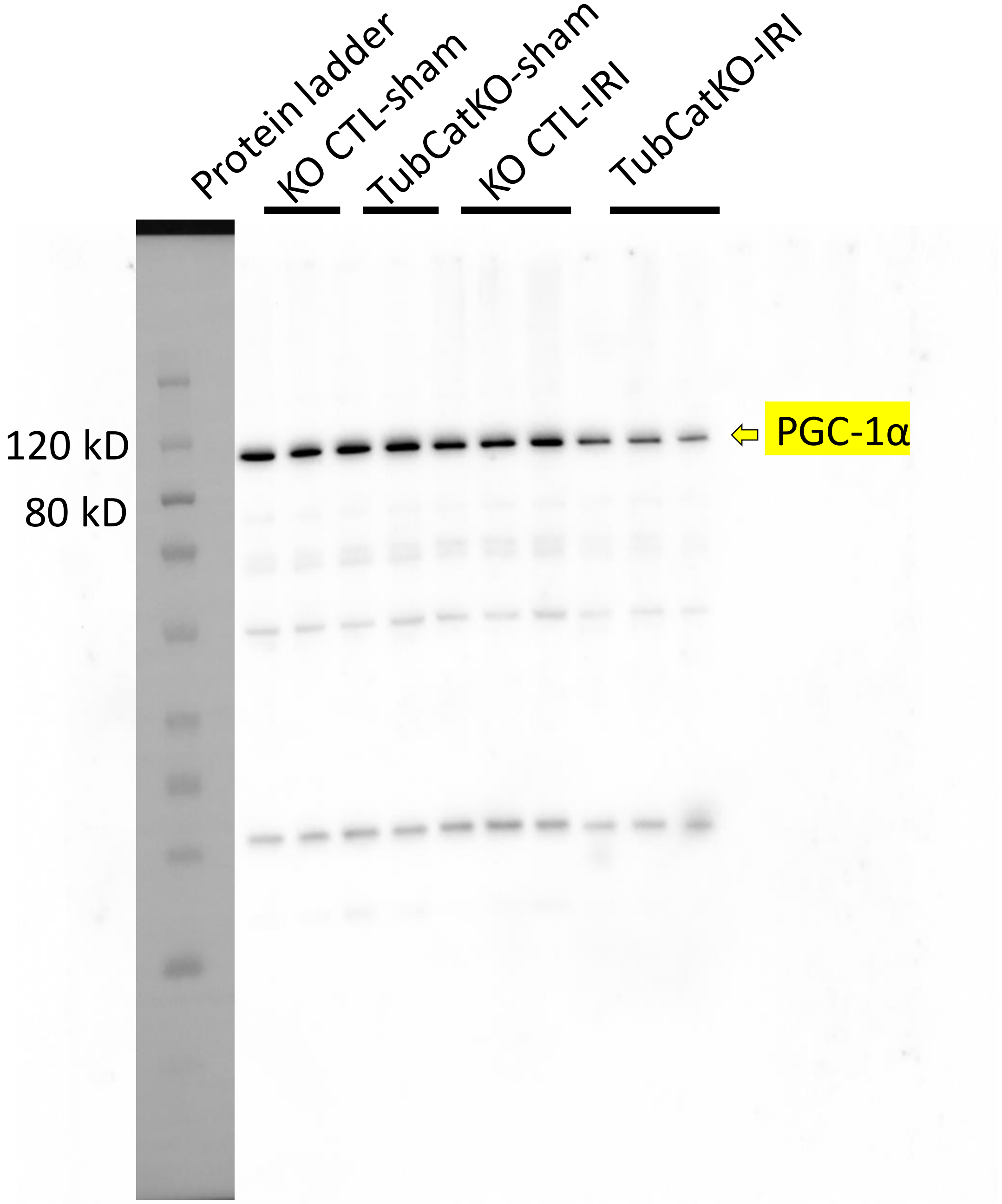

Review files– western blot

40. Fig 5D, β-ACTIN for PGC-1α

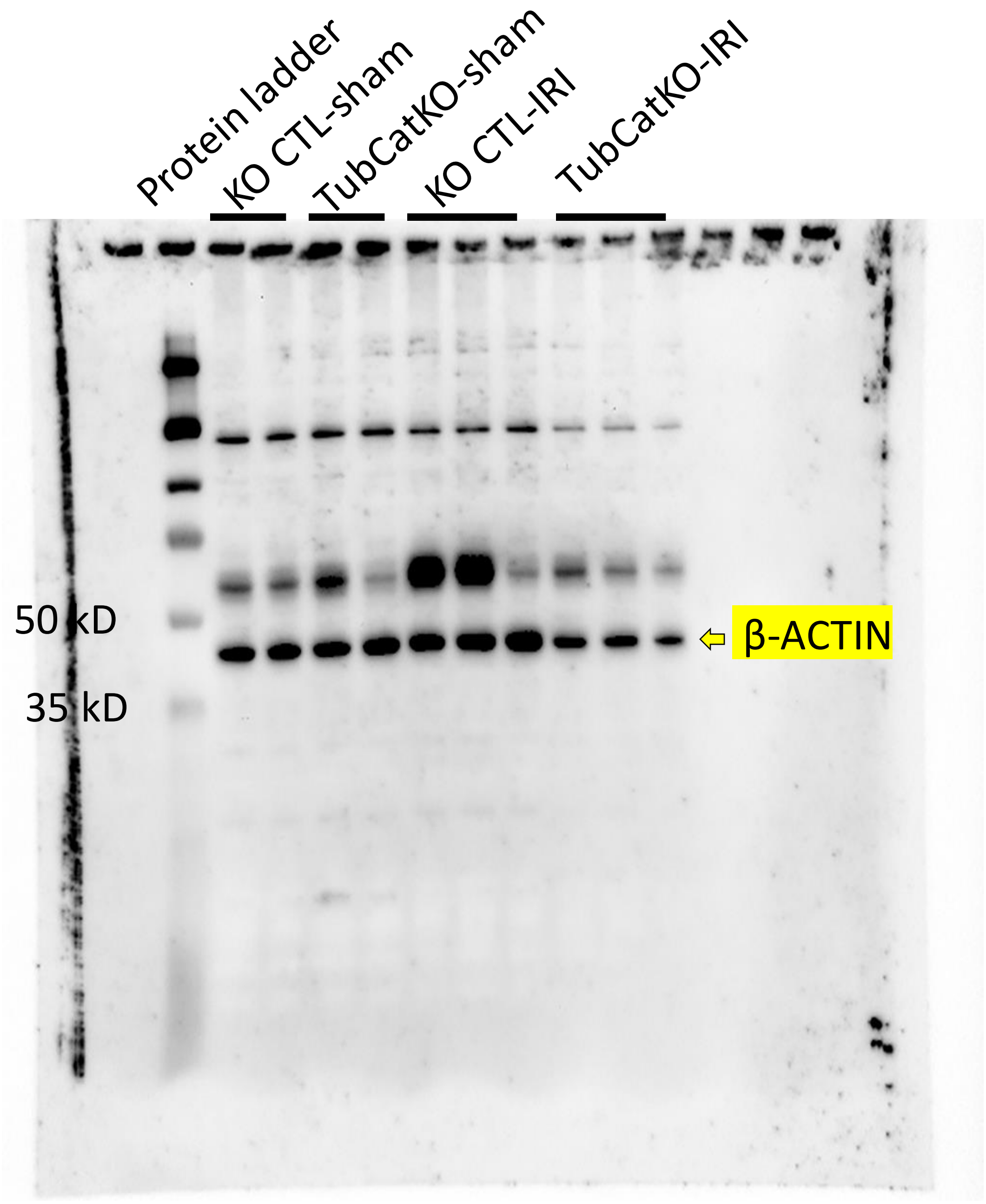

Review files– western blot

41. Fig 5D, NRF1

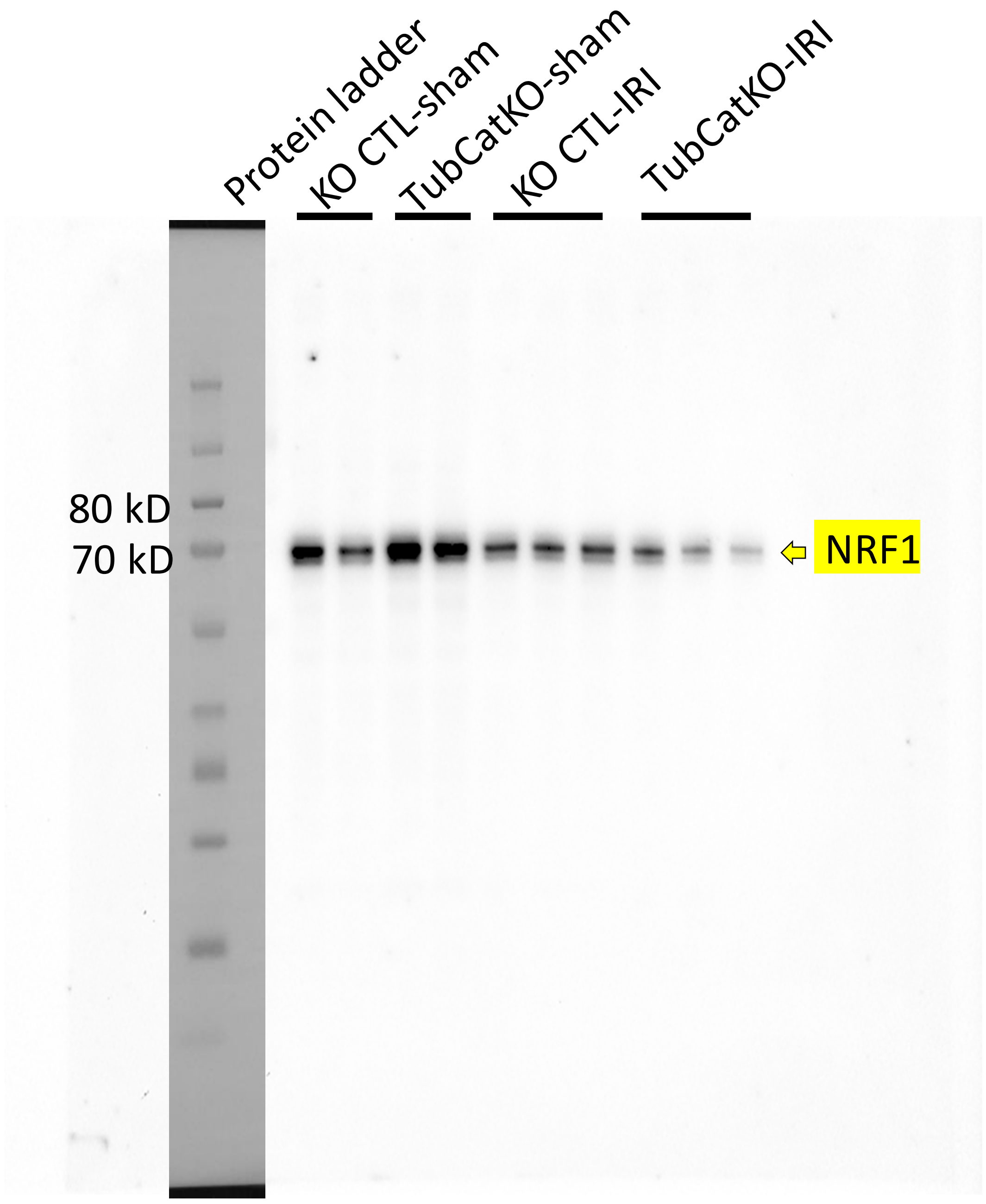

Review files– western blot

42. Fig 5D, β-ACTIN for NRF1, the representative β-ACTIN band chosen showed in Fig 5D

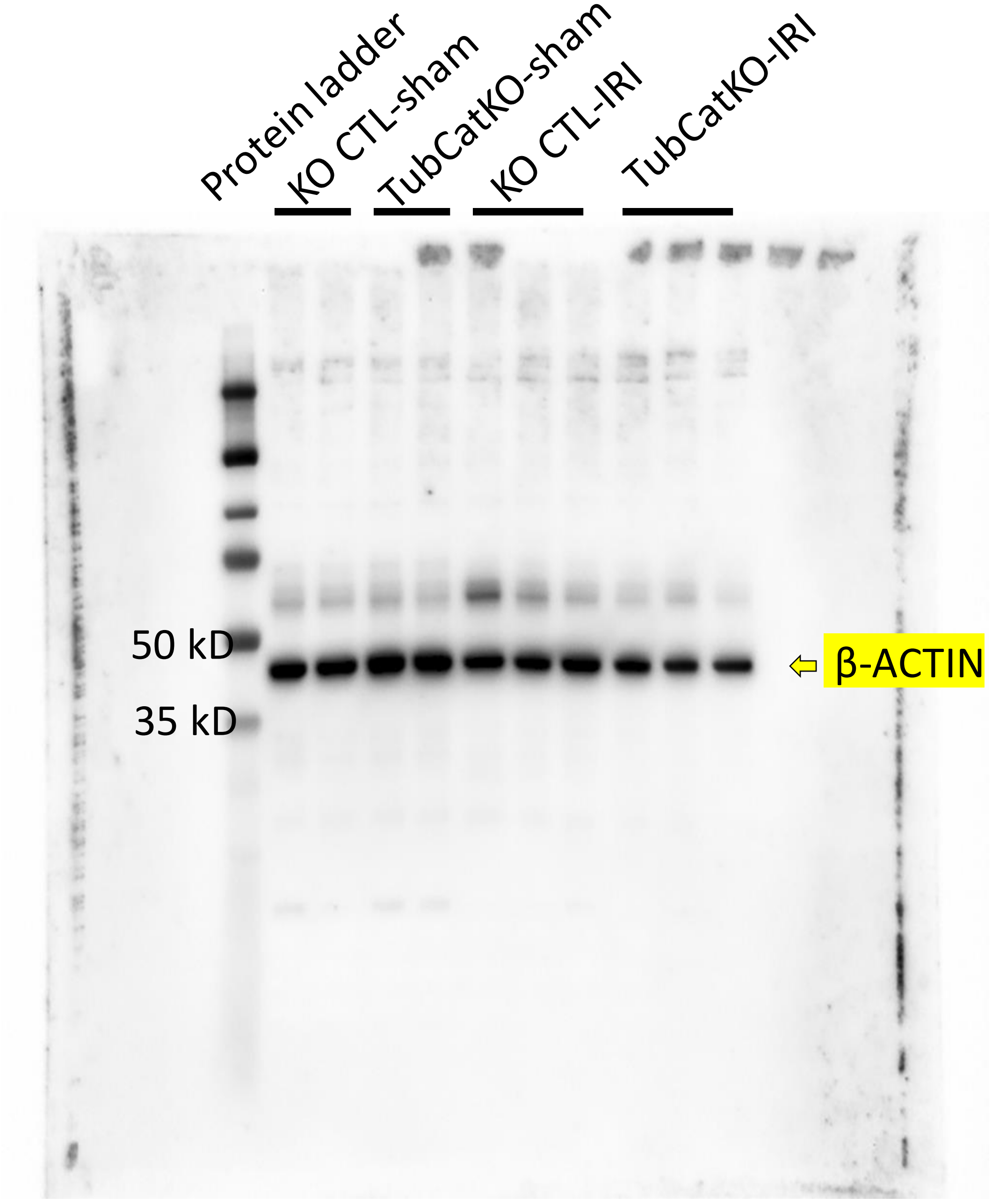

Review files– western blot

43. Fig 5D, TIM23

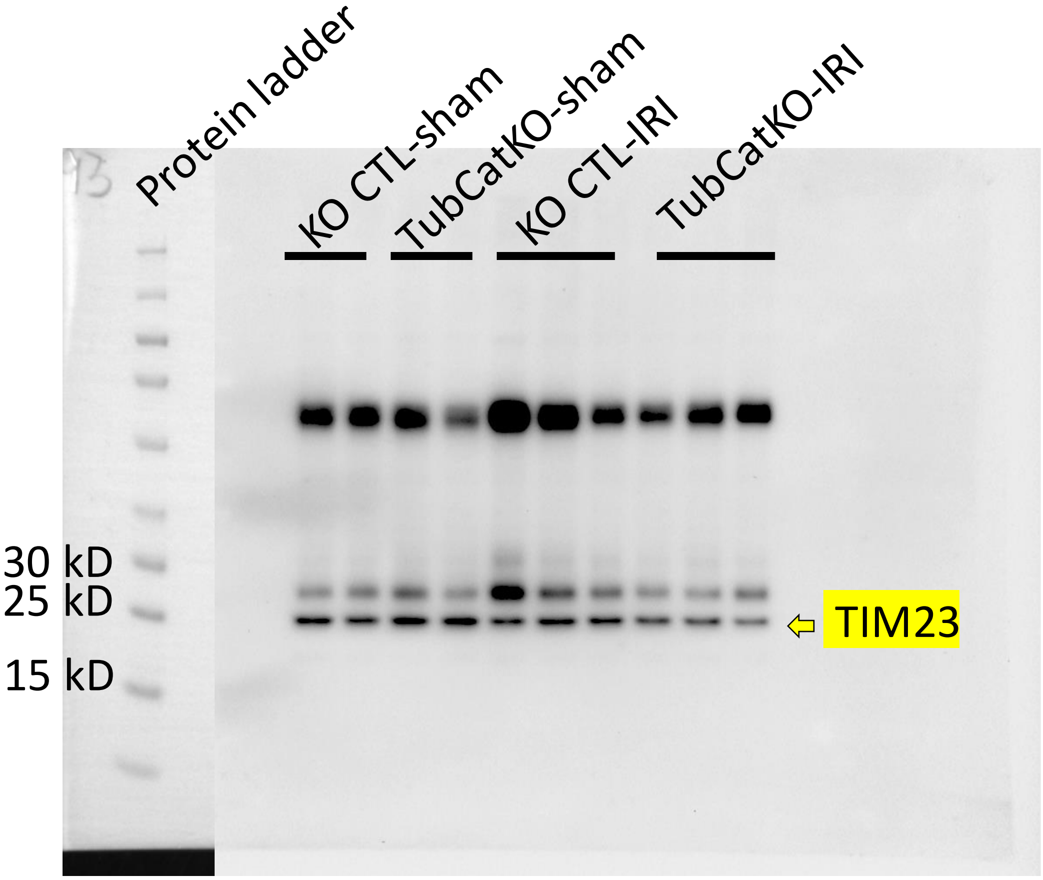

Review files– western blot

44. Fig 5D, β-ACTIN for TIM23

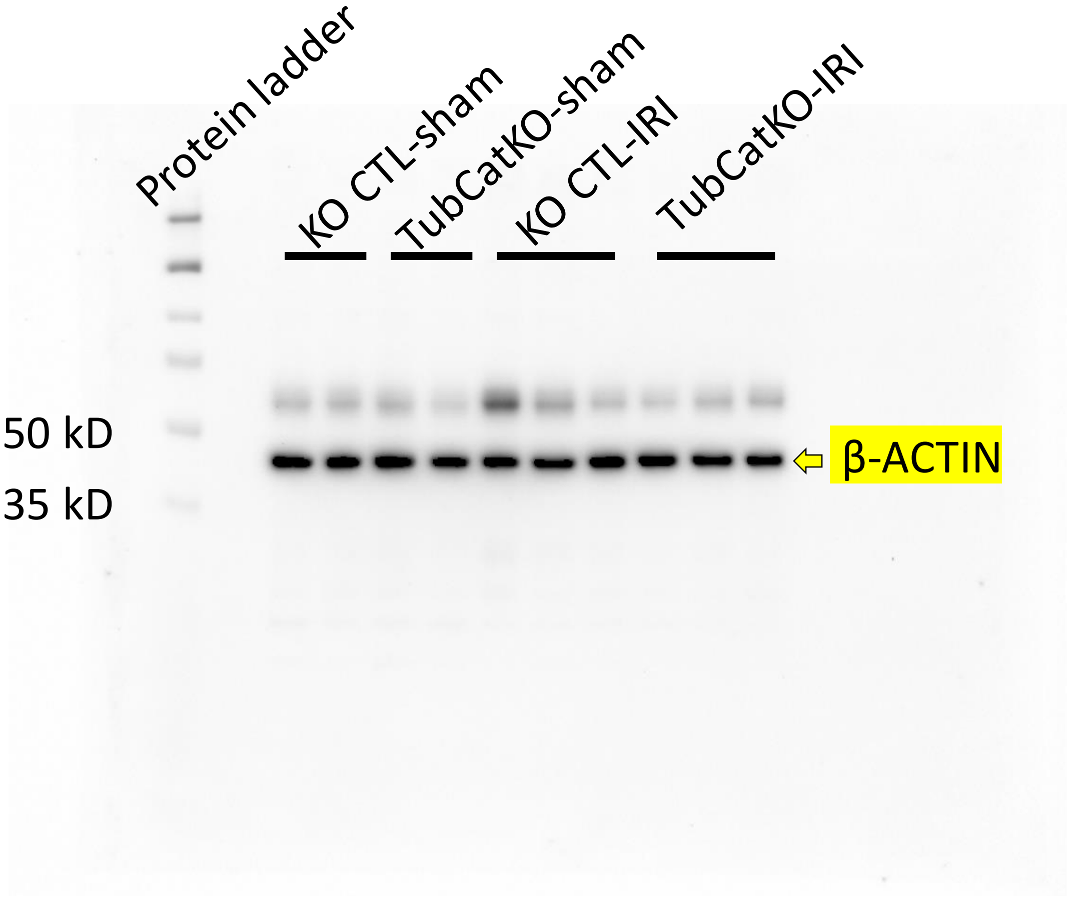

Review files– western blot

45. Fig 5G, FOXO3 and HDAC1

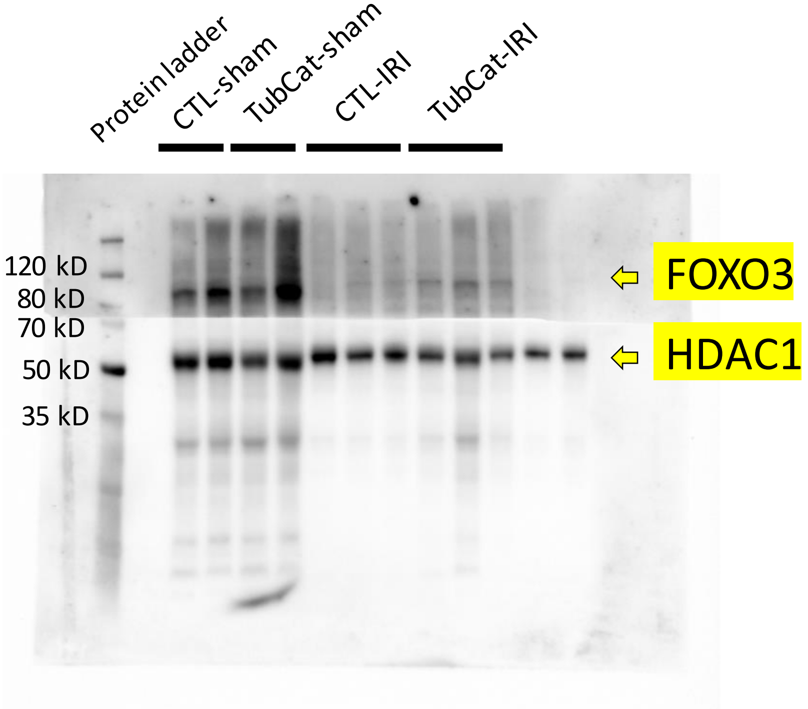

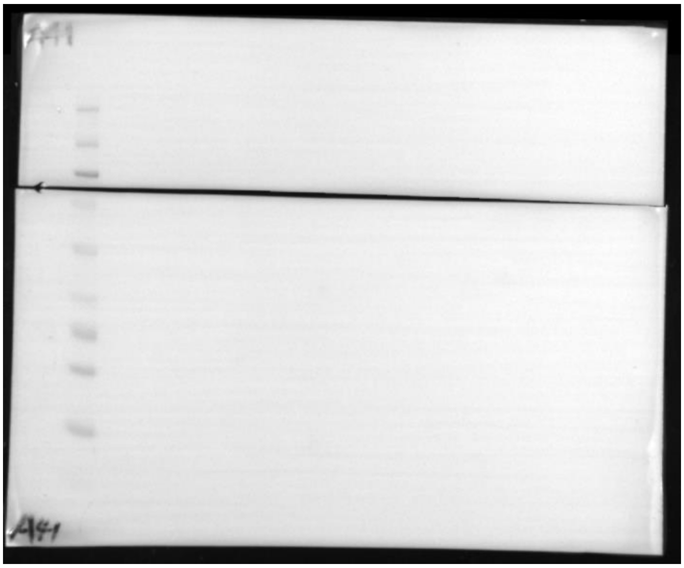

Review files– western blot

46. Fig 5H, FOXO3 and HDAC1

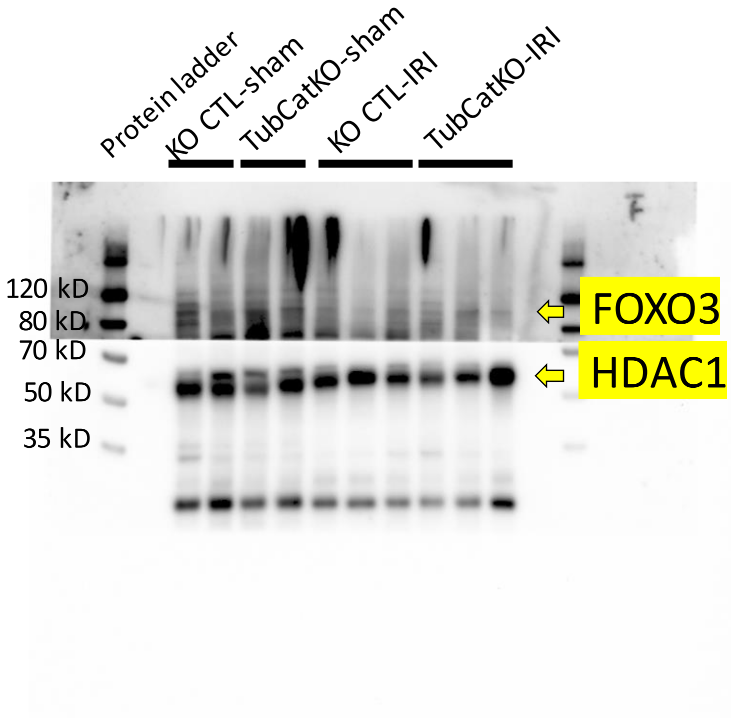

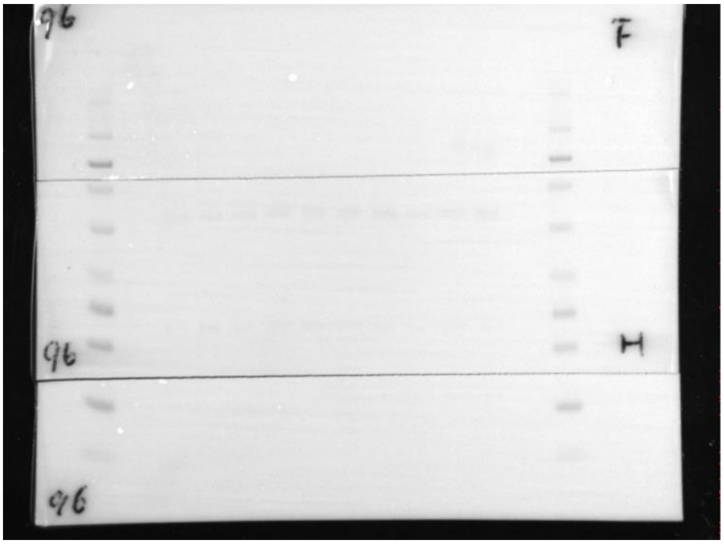

Review files– western blot

47. Fig 6C, PGC-1α

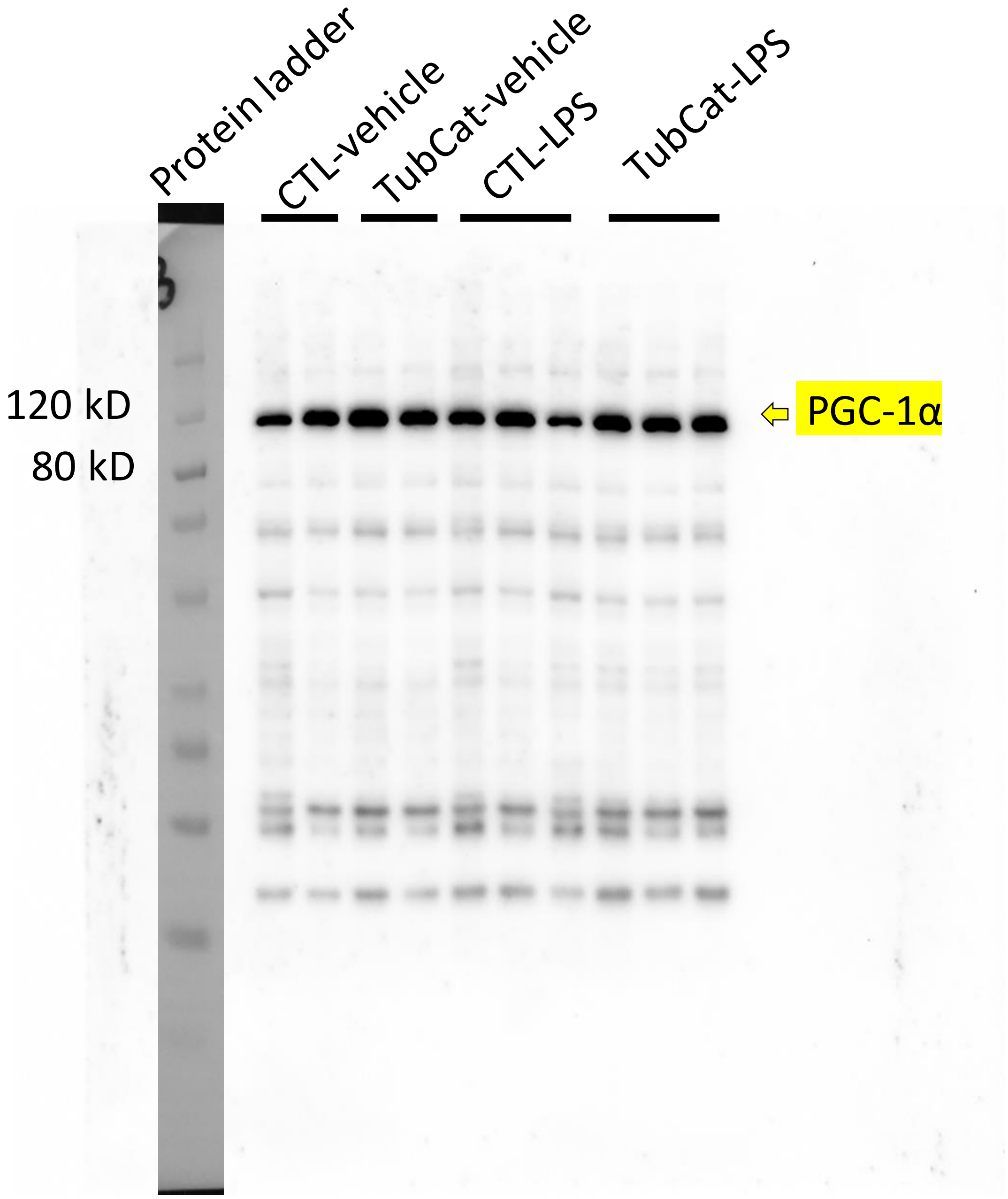

Review files– western blot

48. Fig 6C, β-ACTIN for PGC-1α

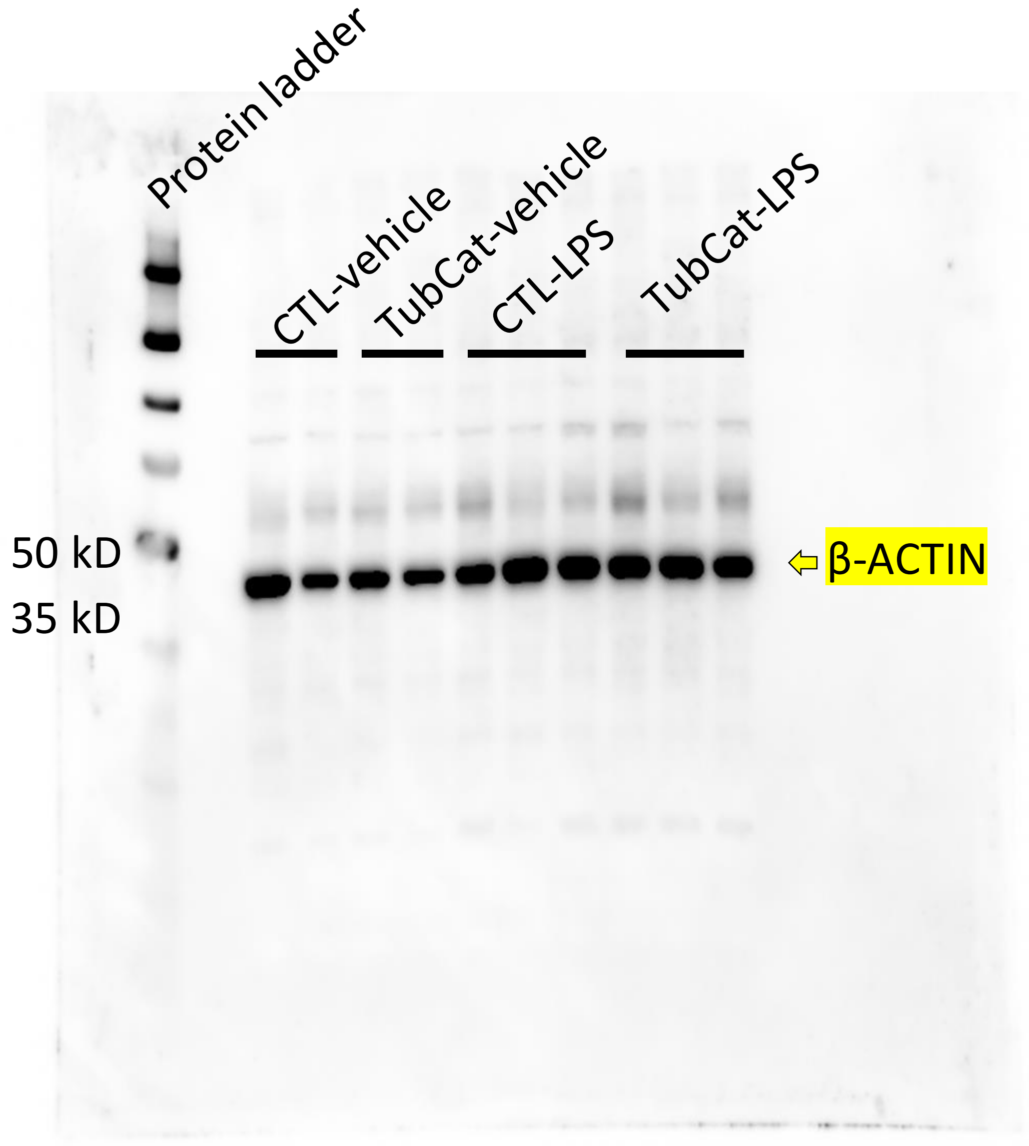

Review files– western blot

49. Fig 6C, NRF1

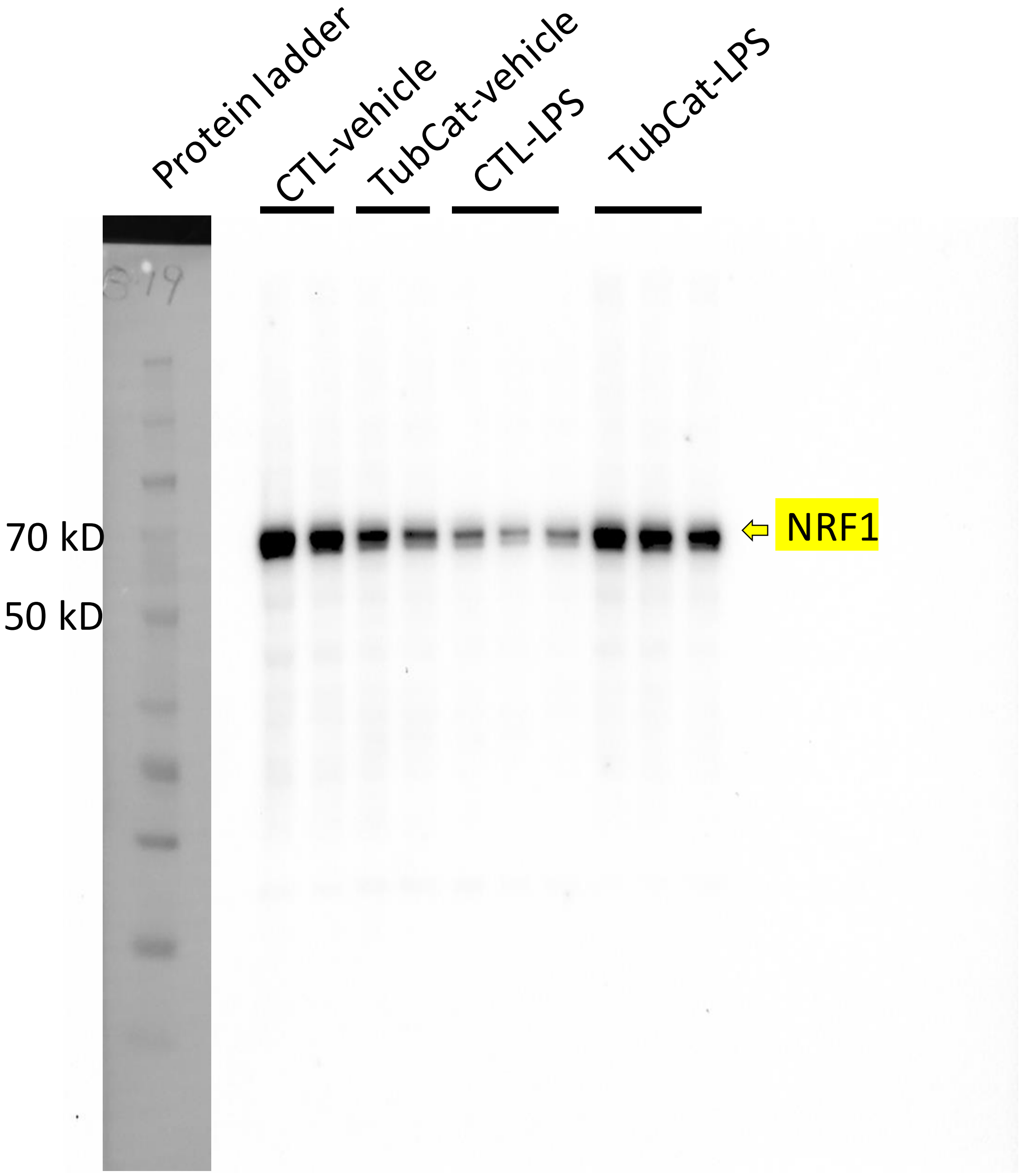

Review files– western blot

50. Fig 6C, β-ACTIN for NRF1, the representative band of β-ACTIN in Fig 6C

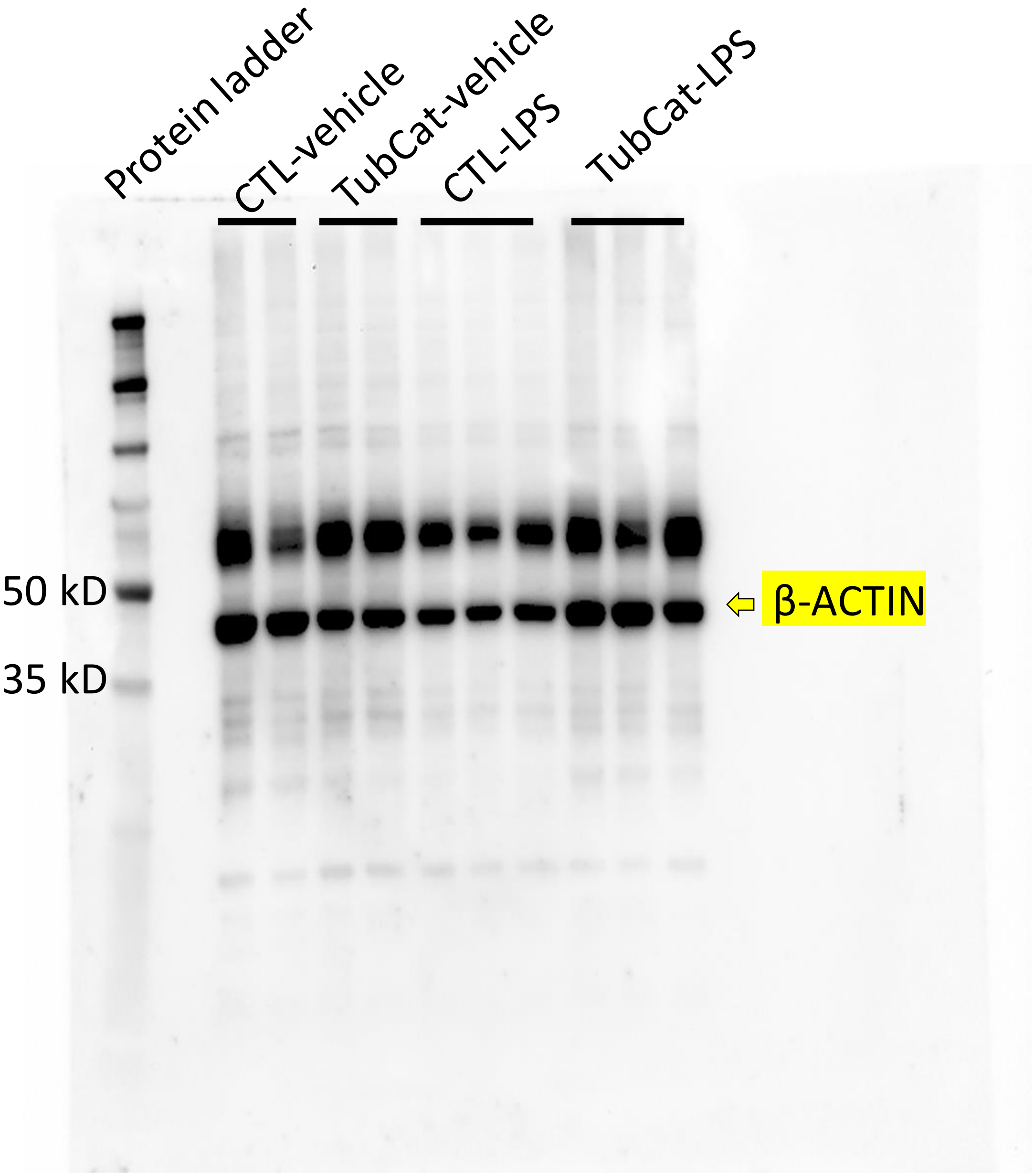

Review files– western blot

51. Fig 6C, TIM23

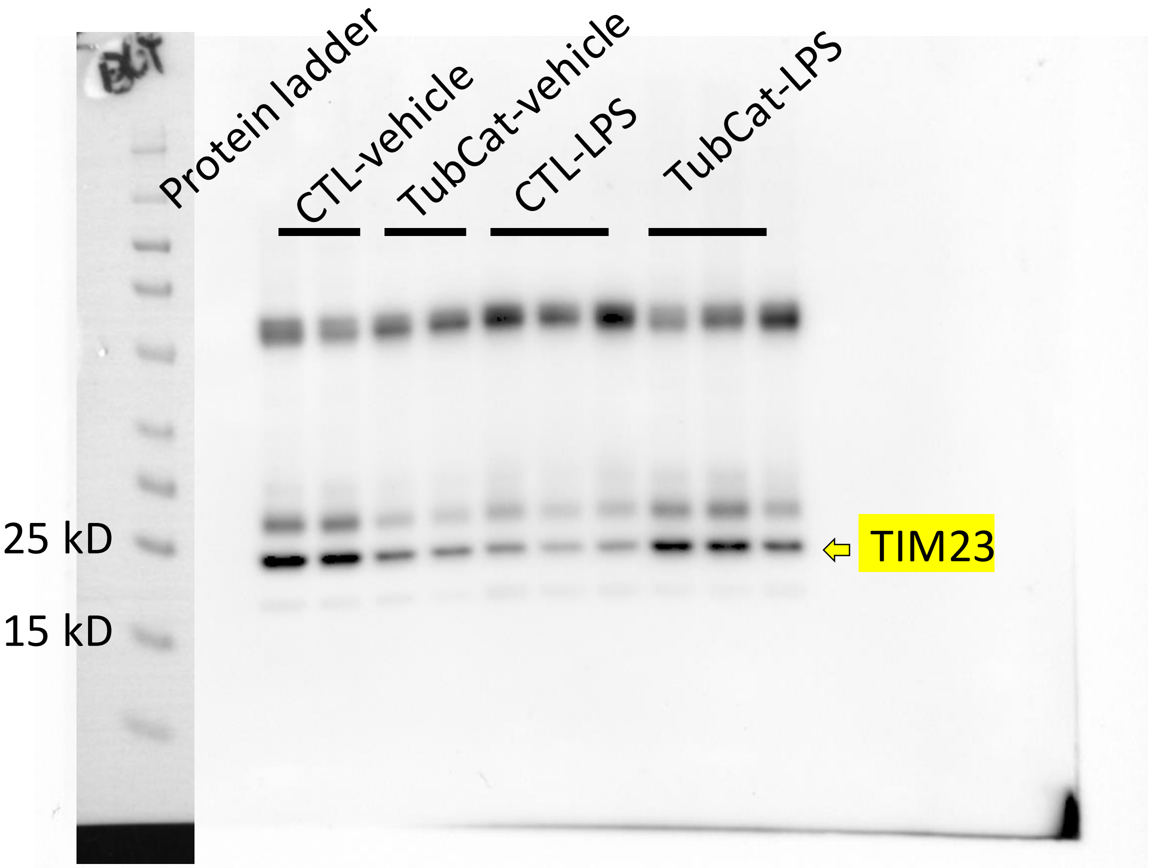

Review files– western blot

52. Fig 6C, β-ACTIN for TIM23

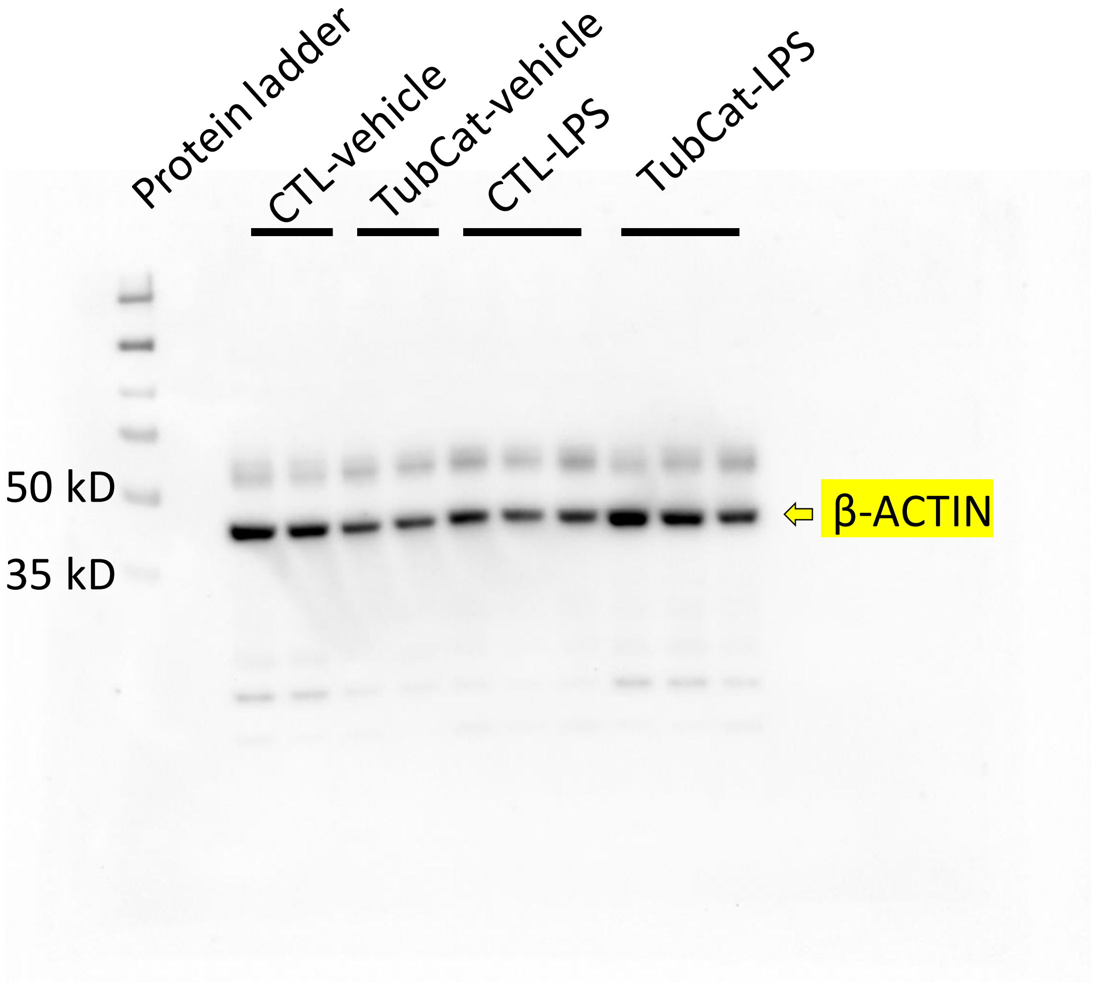

Review files– western blot

53. Fig 6D, PGC-1α

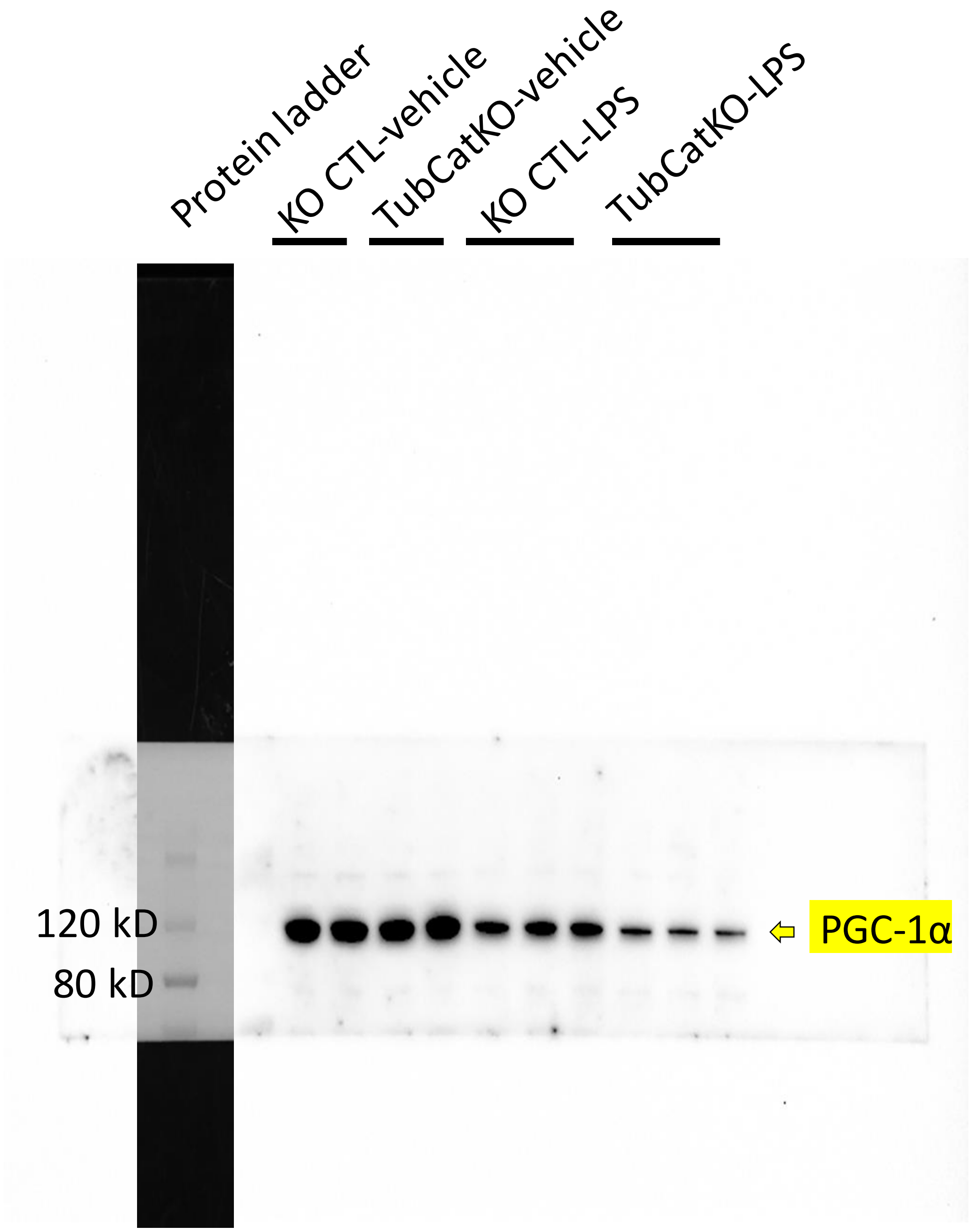

Review files– western blot

54. Fig 6D, β-ACTIN for PGC-1α

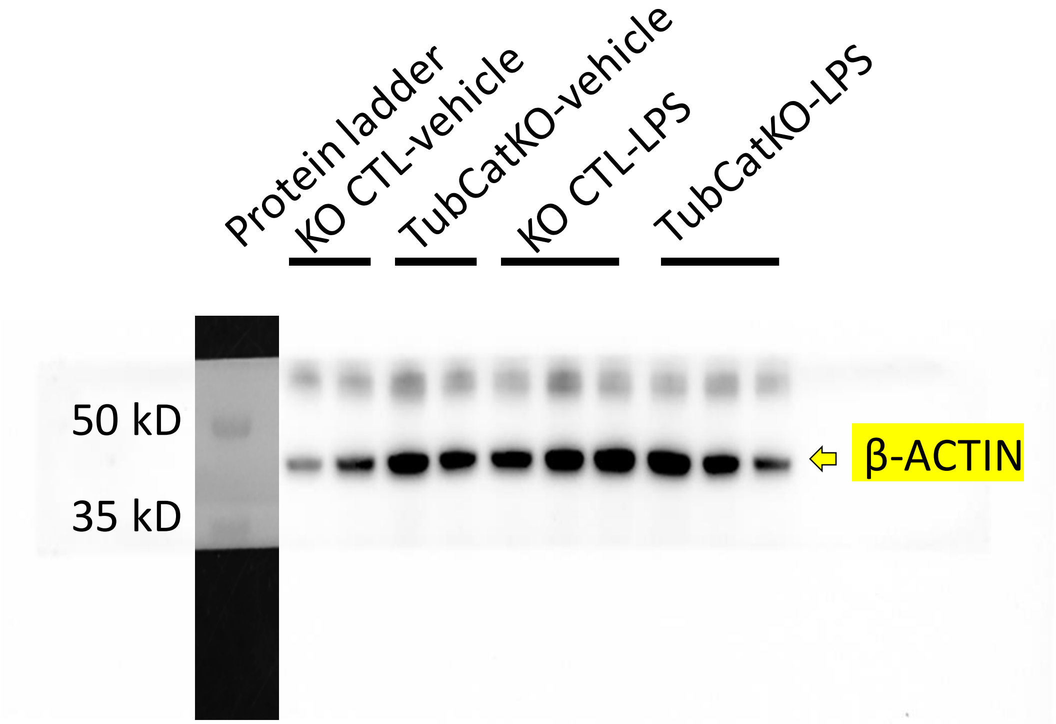

Protein ladder image of Fig 6D PGC-1α and its loading control:

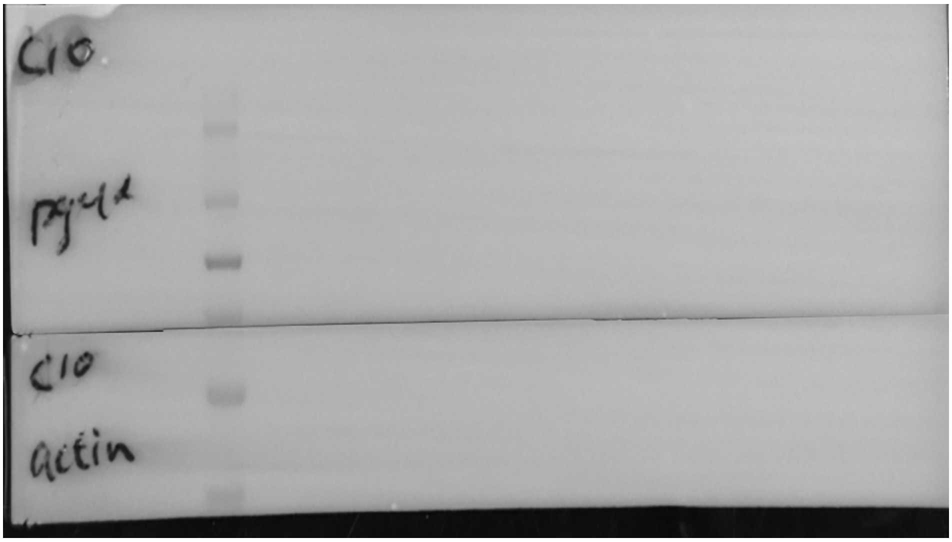

Review files– western blot

55. Fig 6D, NRF1

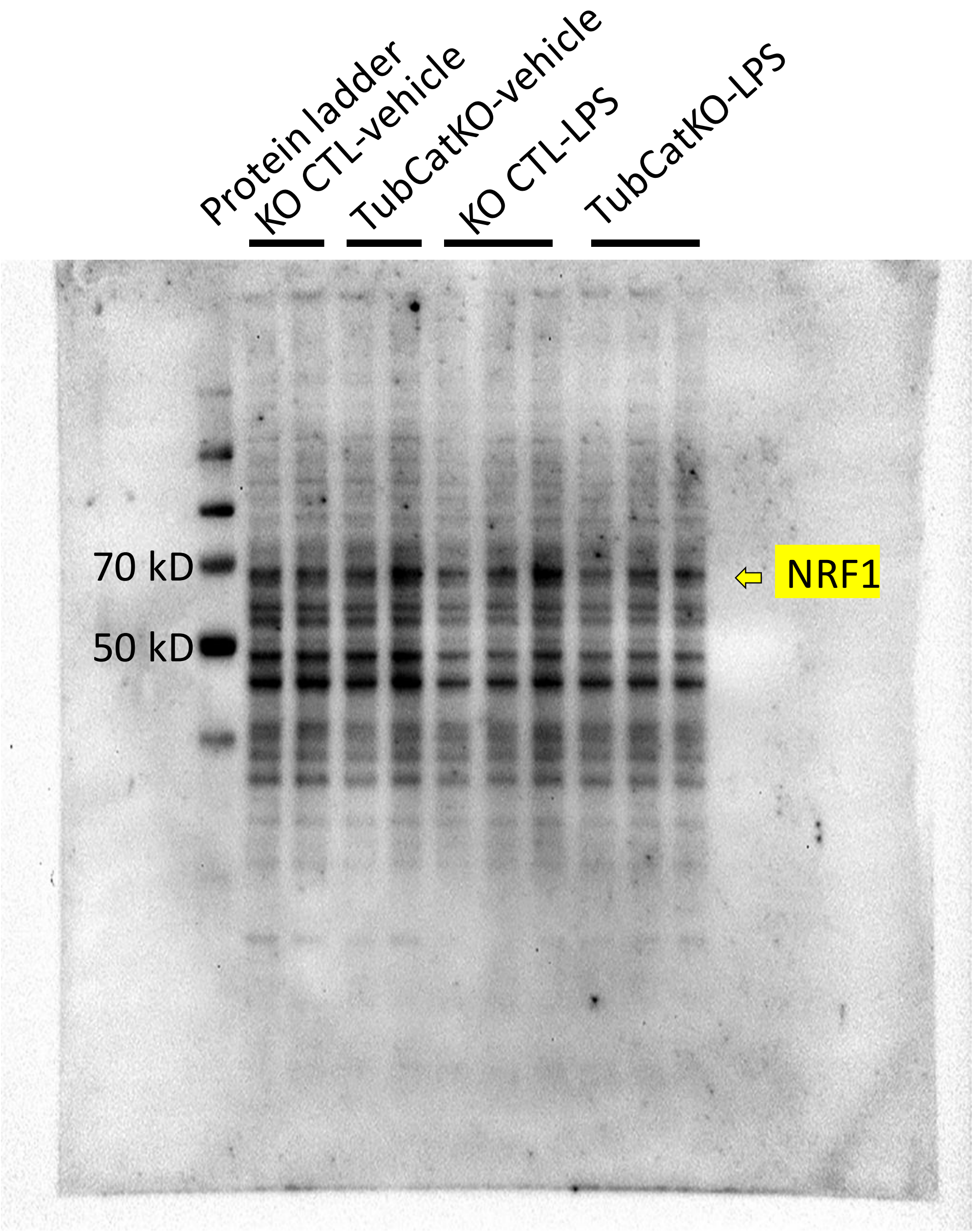

Review files– western blot

56. Fig 6D, β-ACTIN for NRF1

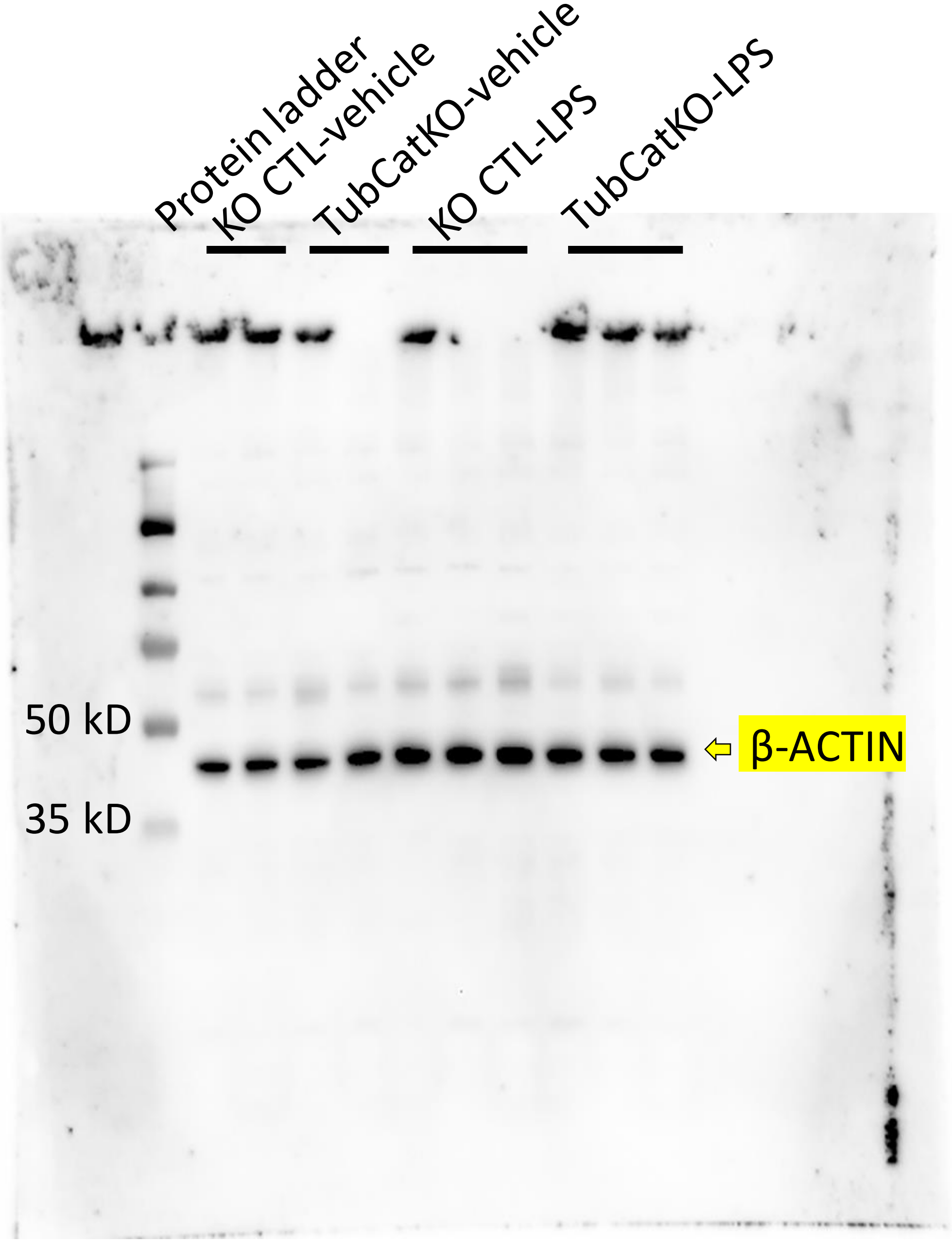

Review files– western blot

57. Fig 6D, TIM23

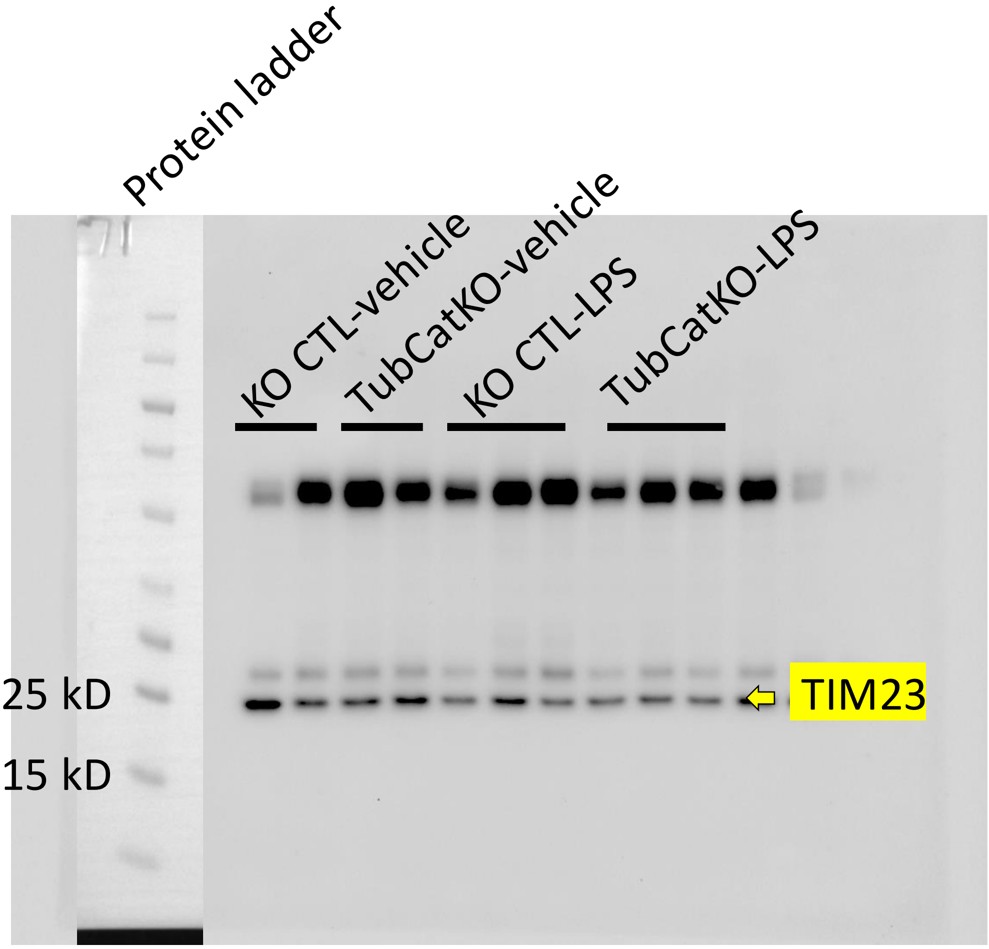

Review files– western blot

58. Fig 6D, β-ACTIN for TIM23

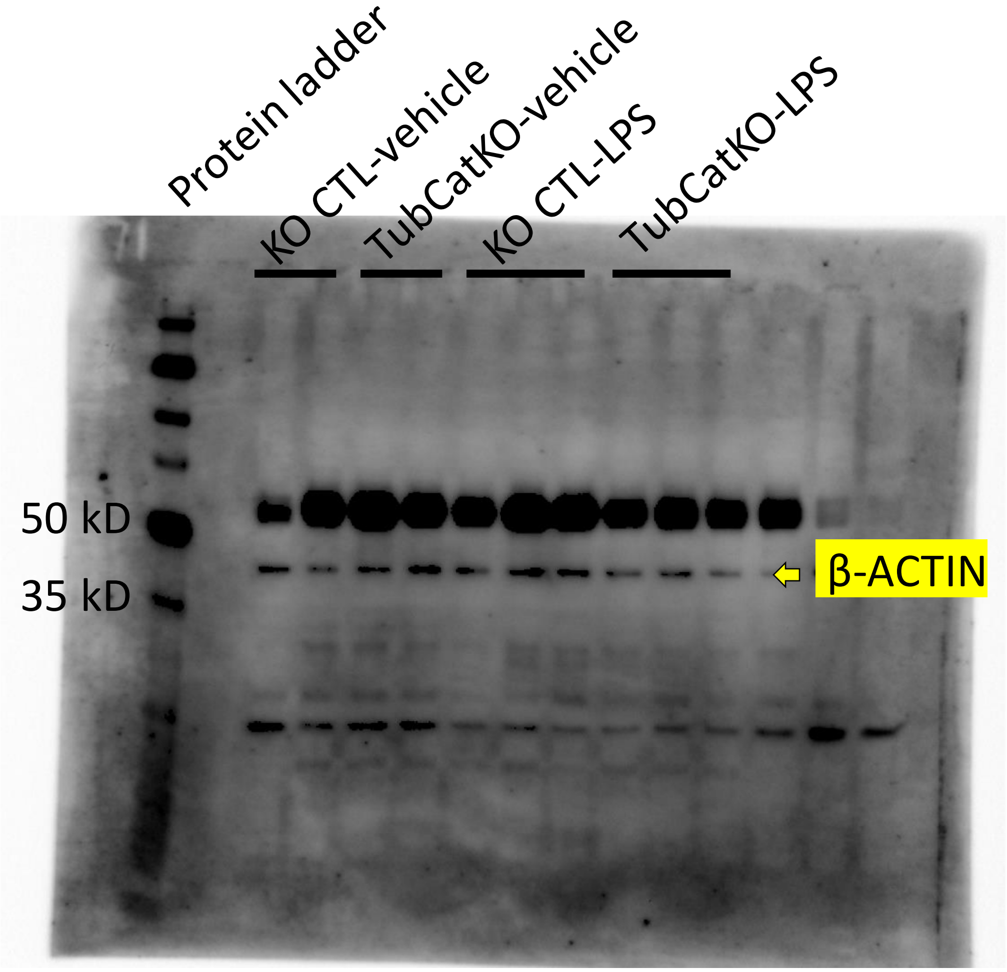

Review files– western blot

59. Fig 6G, FOXO3 and HDAC1

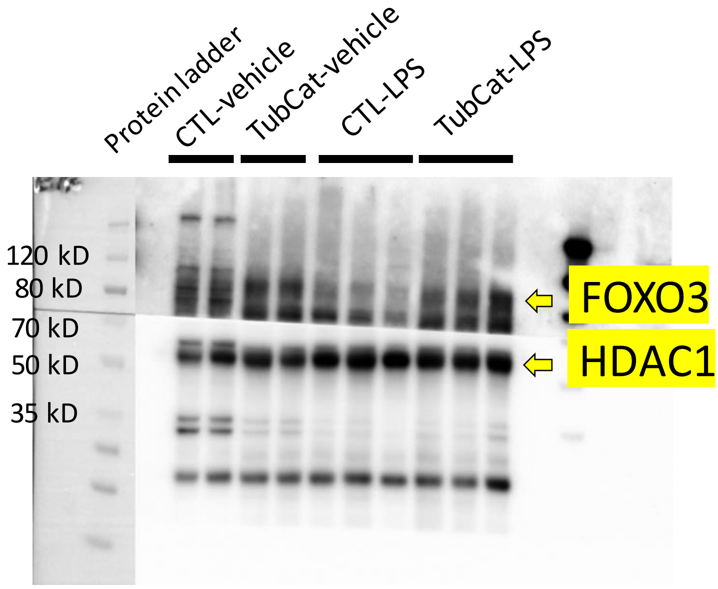

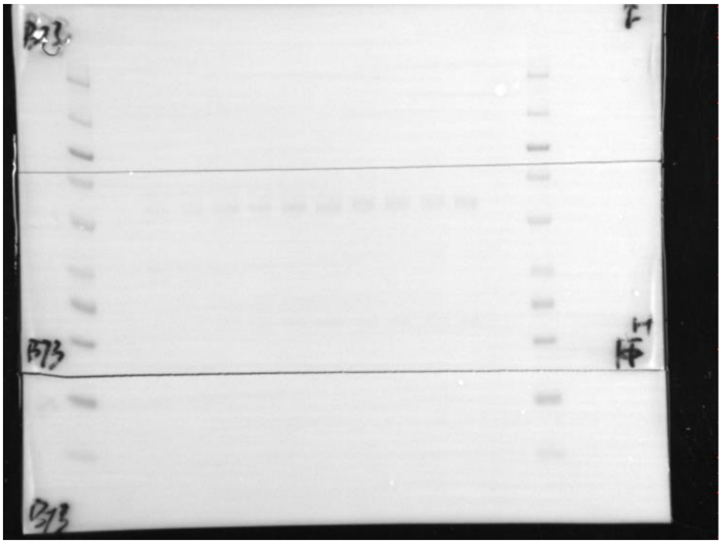

Review files– western blot

60. Fig 6H, FOXO3 and HDAC1

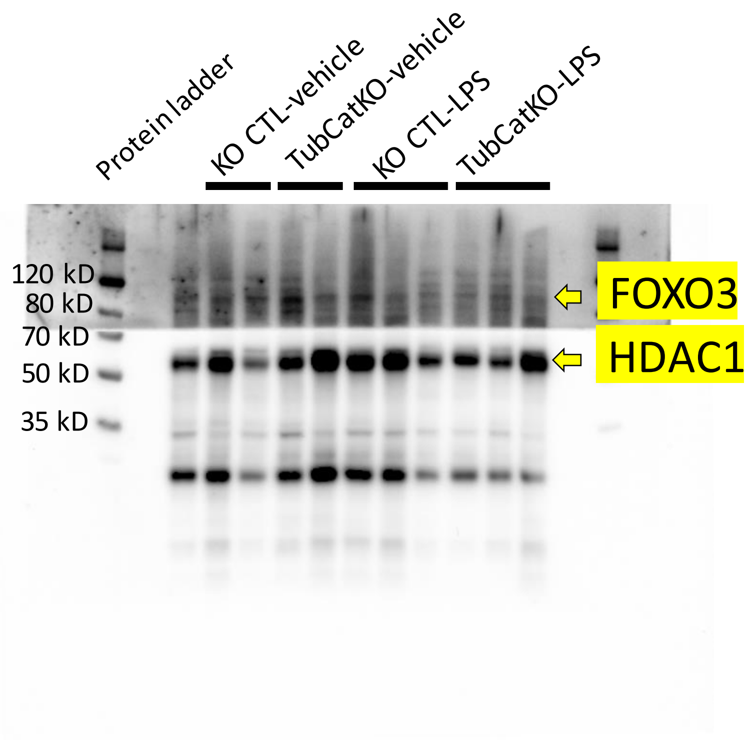

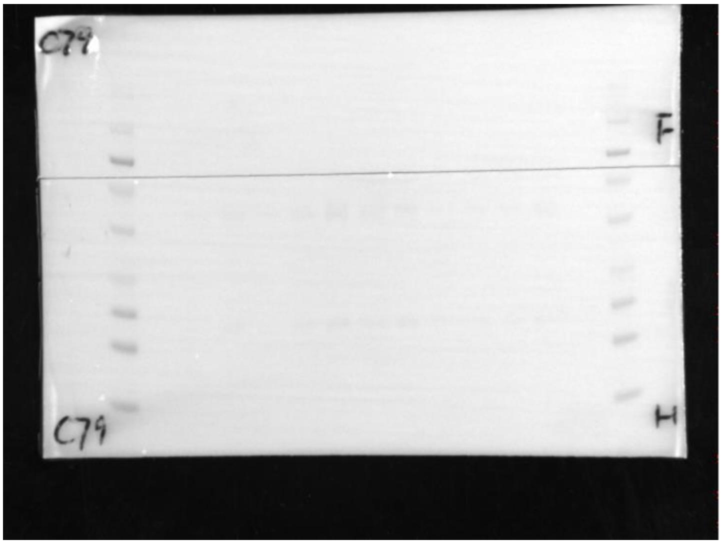

Review files– western blot

61. Fig 7A, OPA1

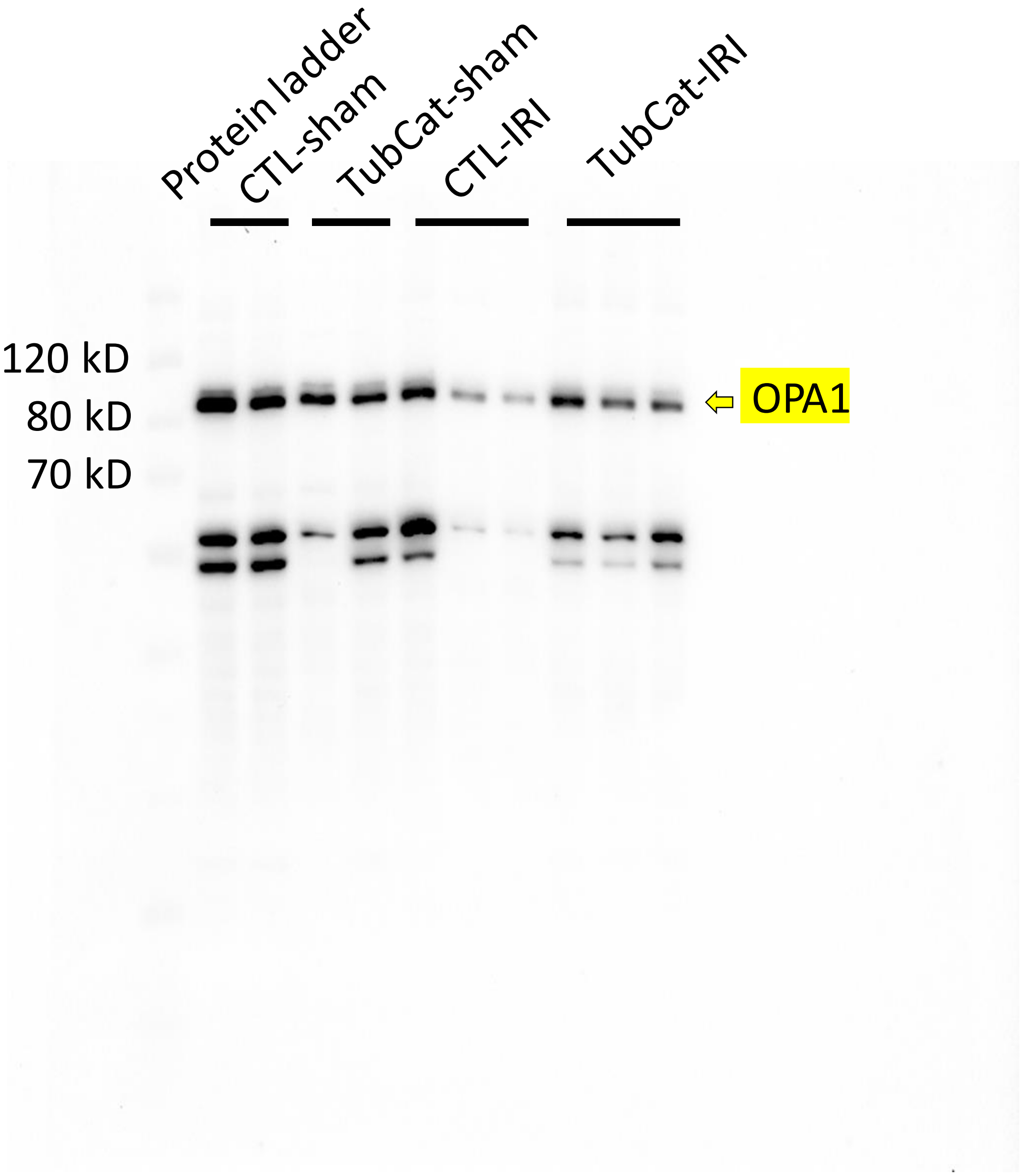

Review files– western blot

62. Fig 7A, β-ACTIN for OPA1, the representative band showed in Fig 7A

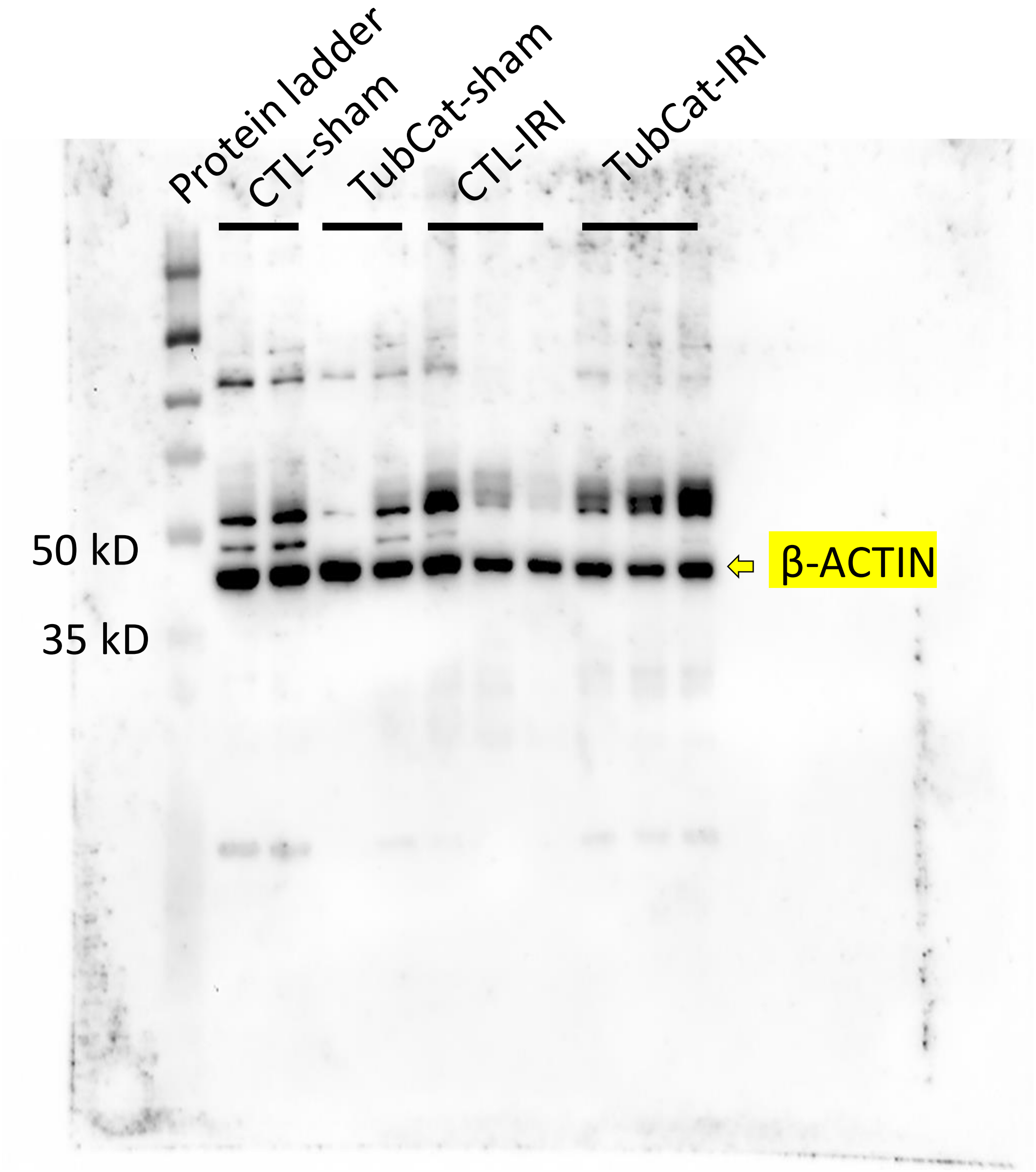

Review files– western blot

63. Fig 7A, MFN2

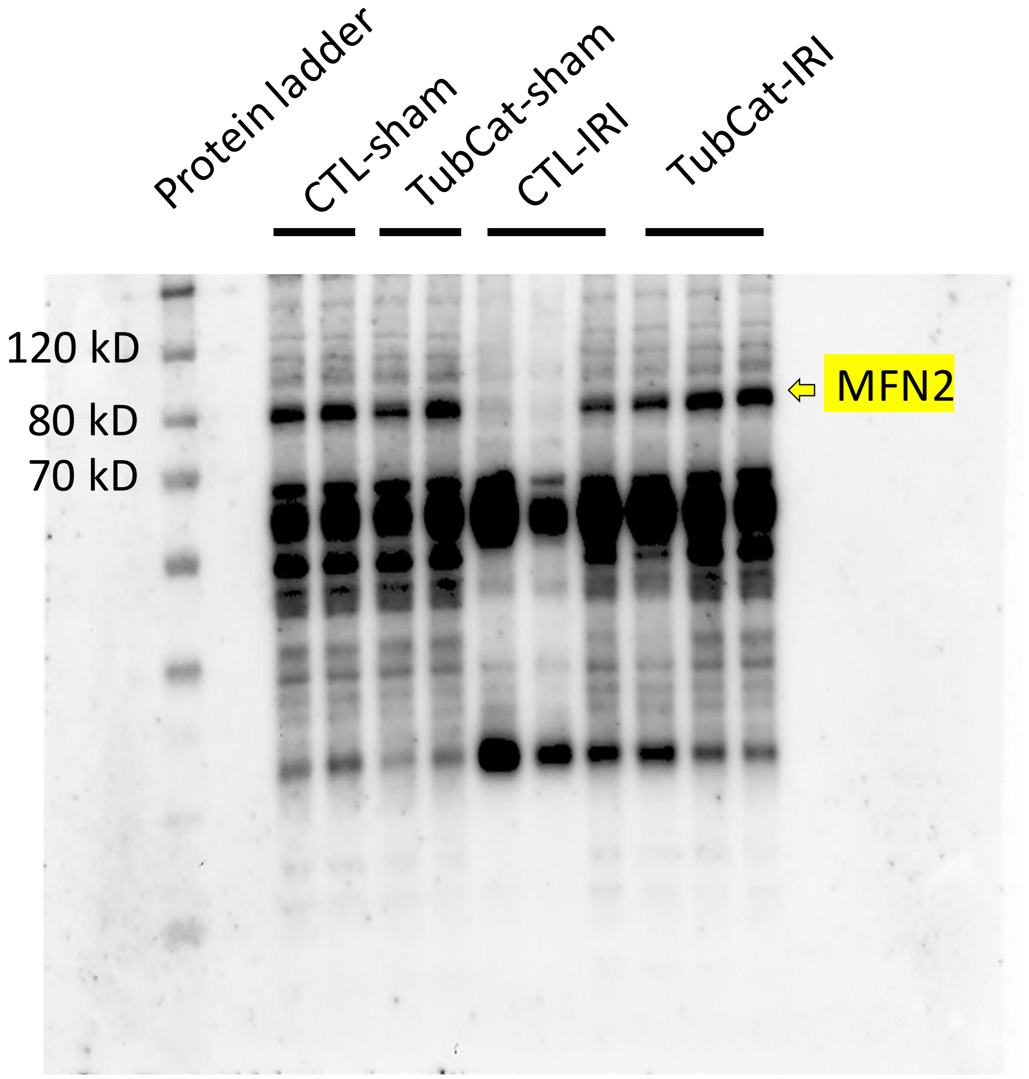

Review files– western blot

64. Fig 7A, β-ACTIN for MFN2

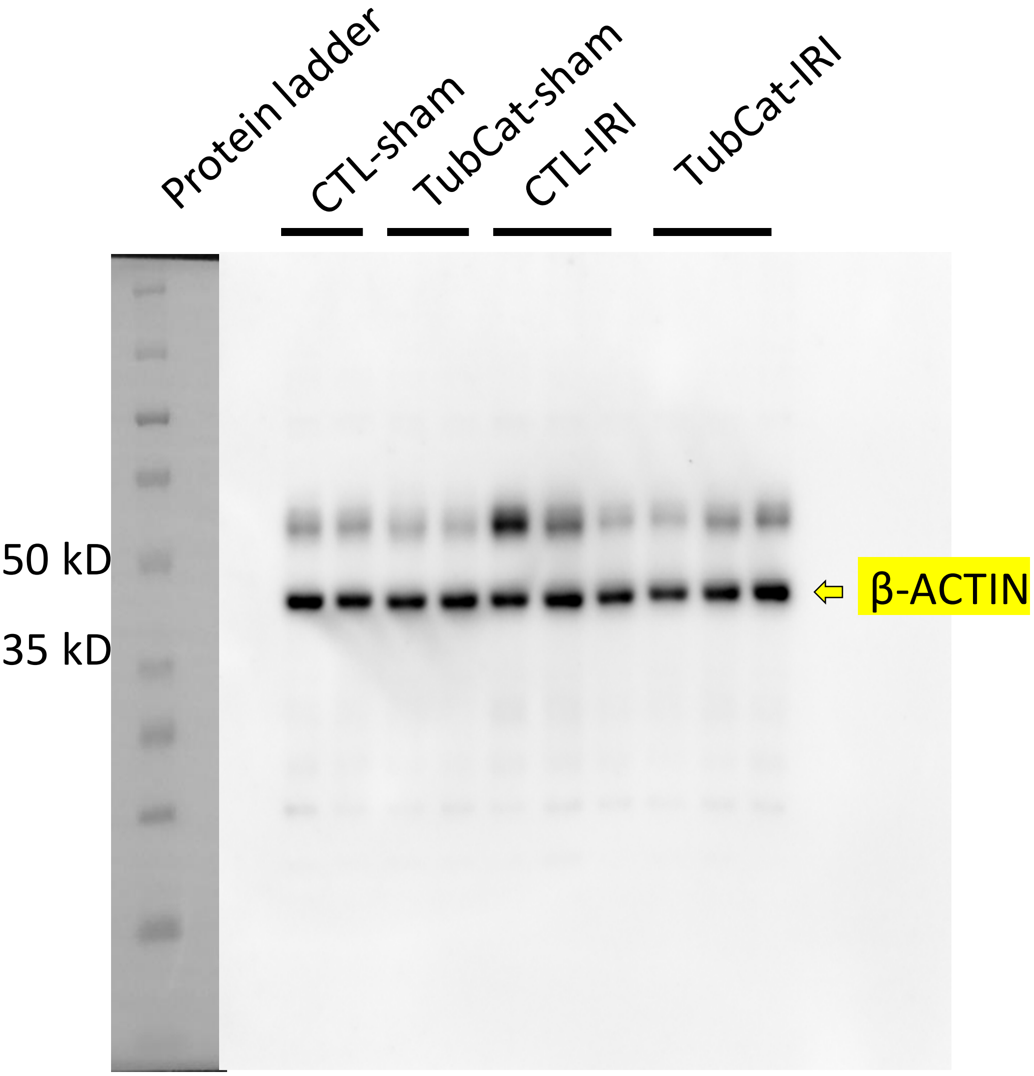

Review files– western blot

65. Fig 7A, DRP1

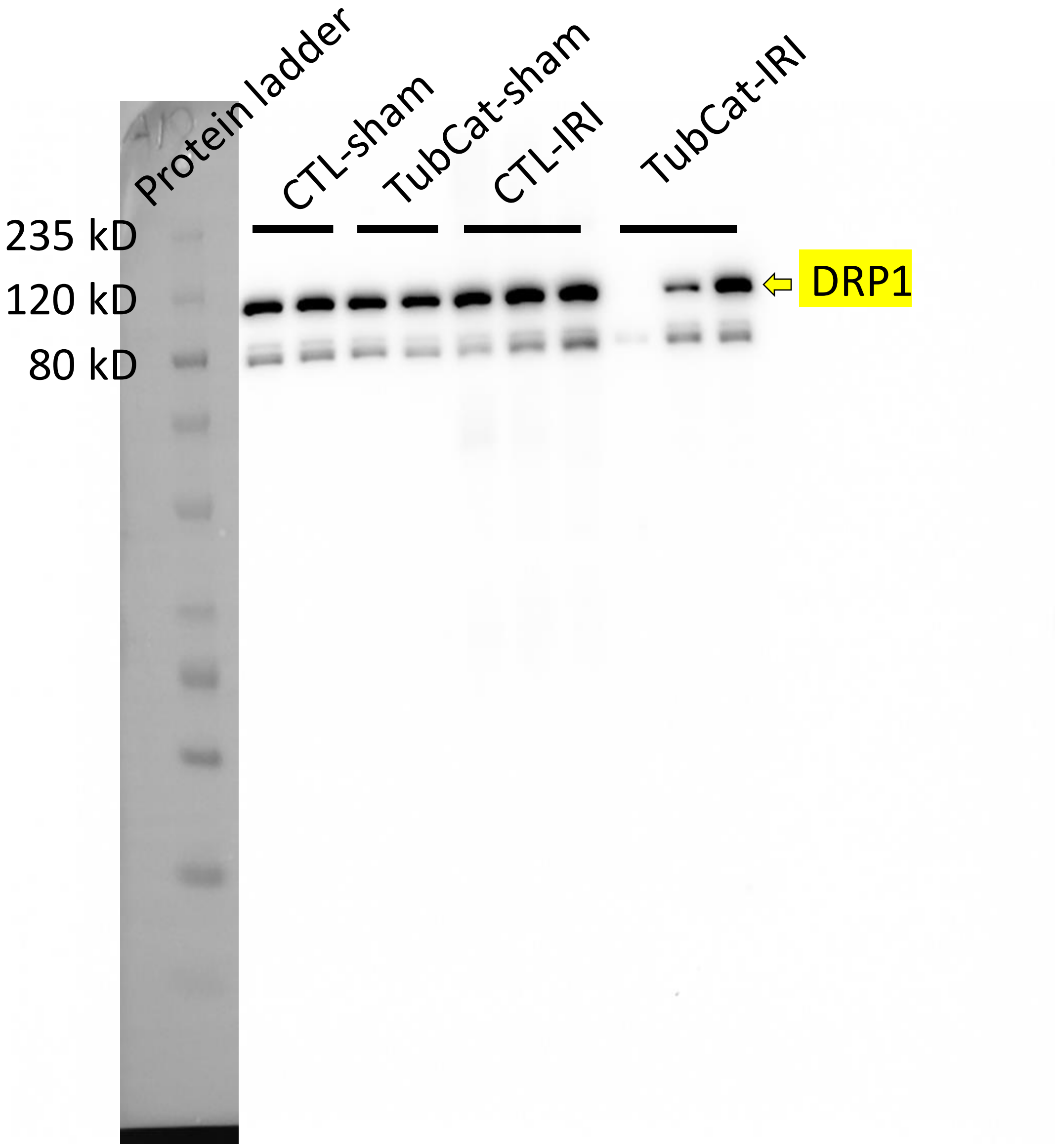

Review files– western blot

66. Fig 7A, β-ACTIN for DRP1

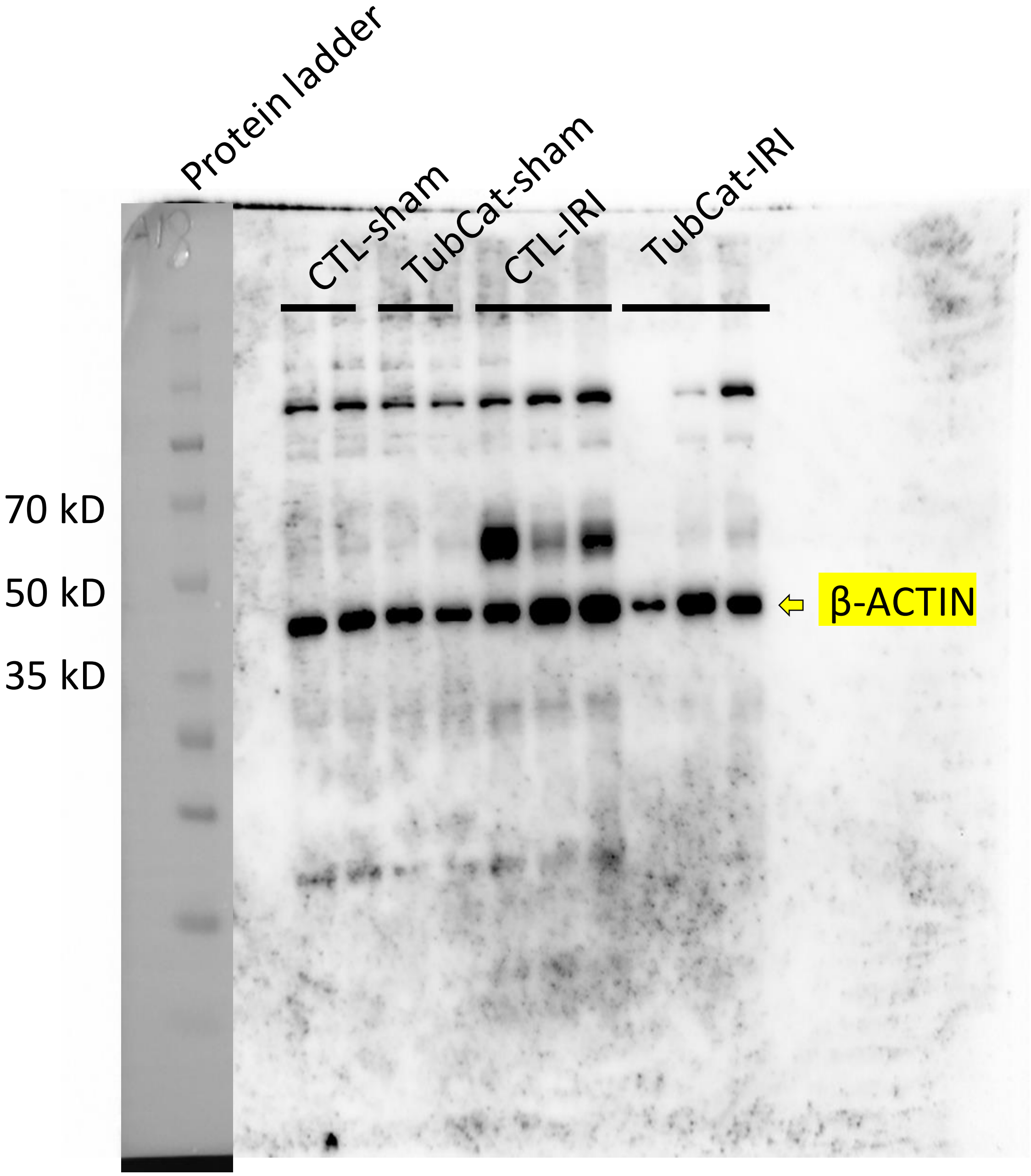

Review files– western blot

67. Fig 7B, OPA1

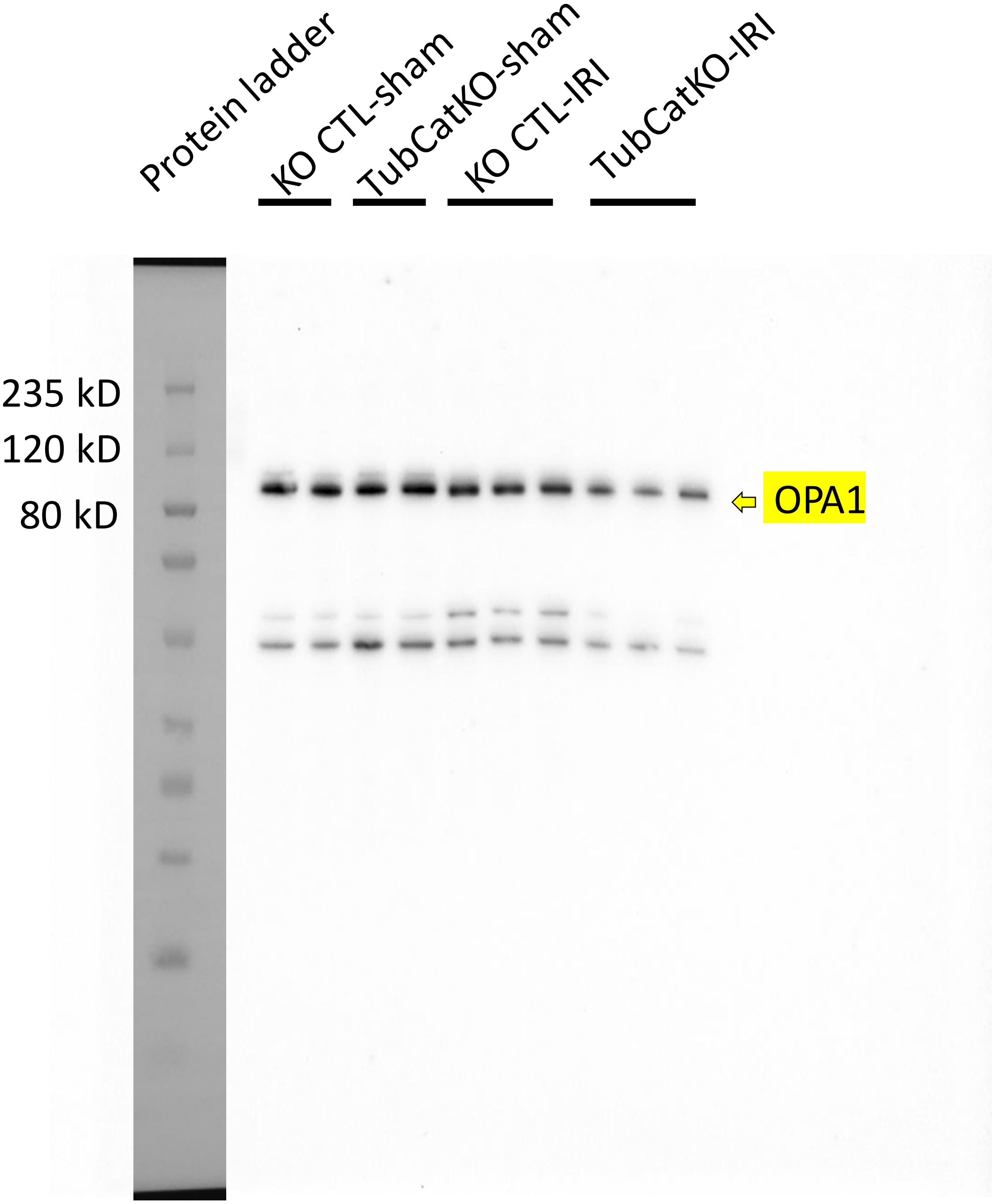

Review files– western blot

68. Fig 7A, β-ACTIN for OPA1

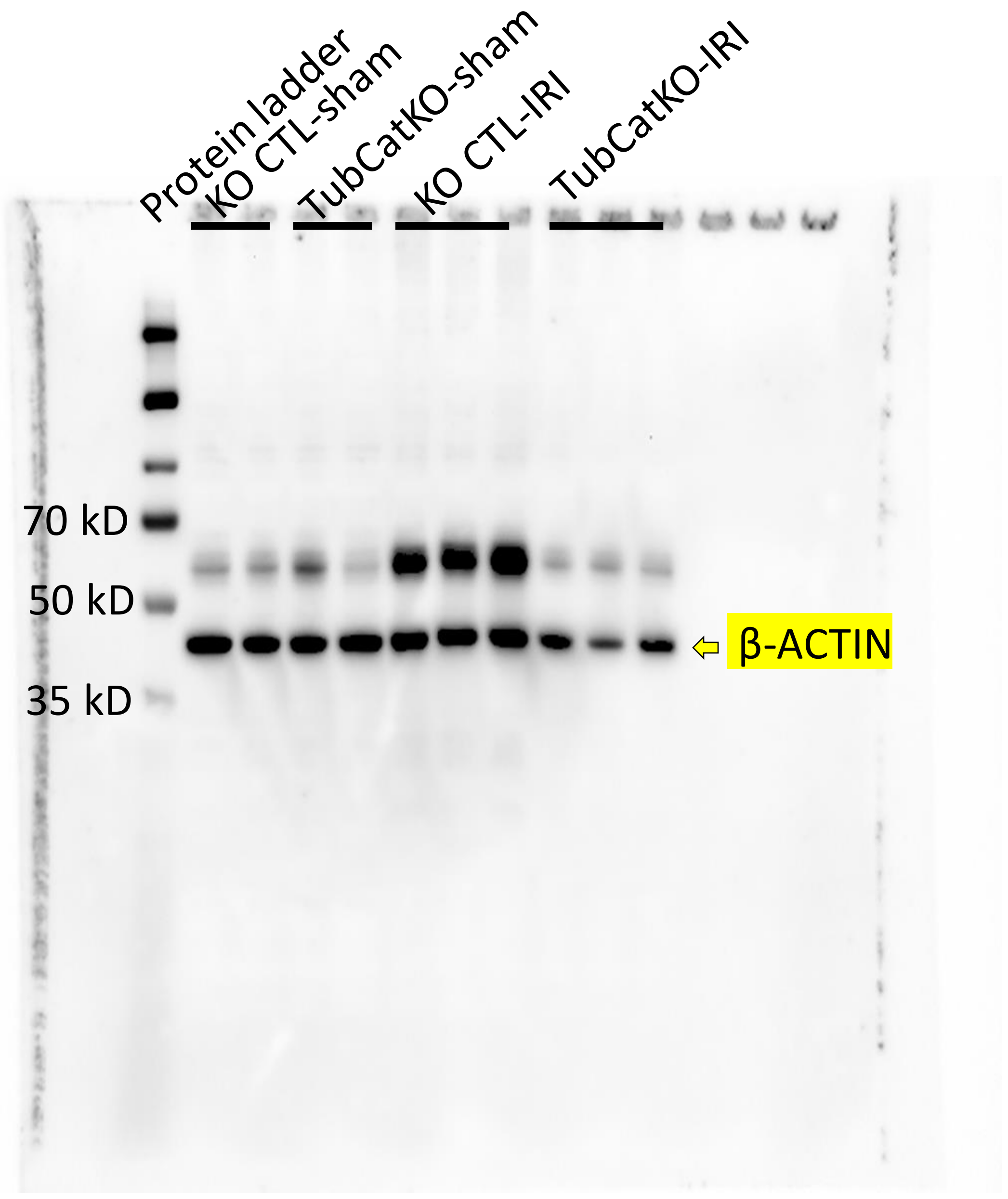

Review files– western blot

69. Fig 7B, MFN2

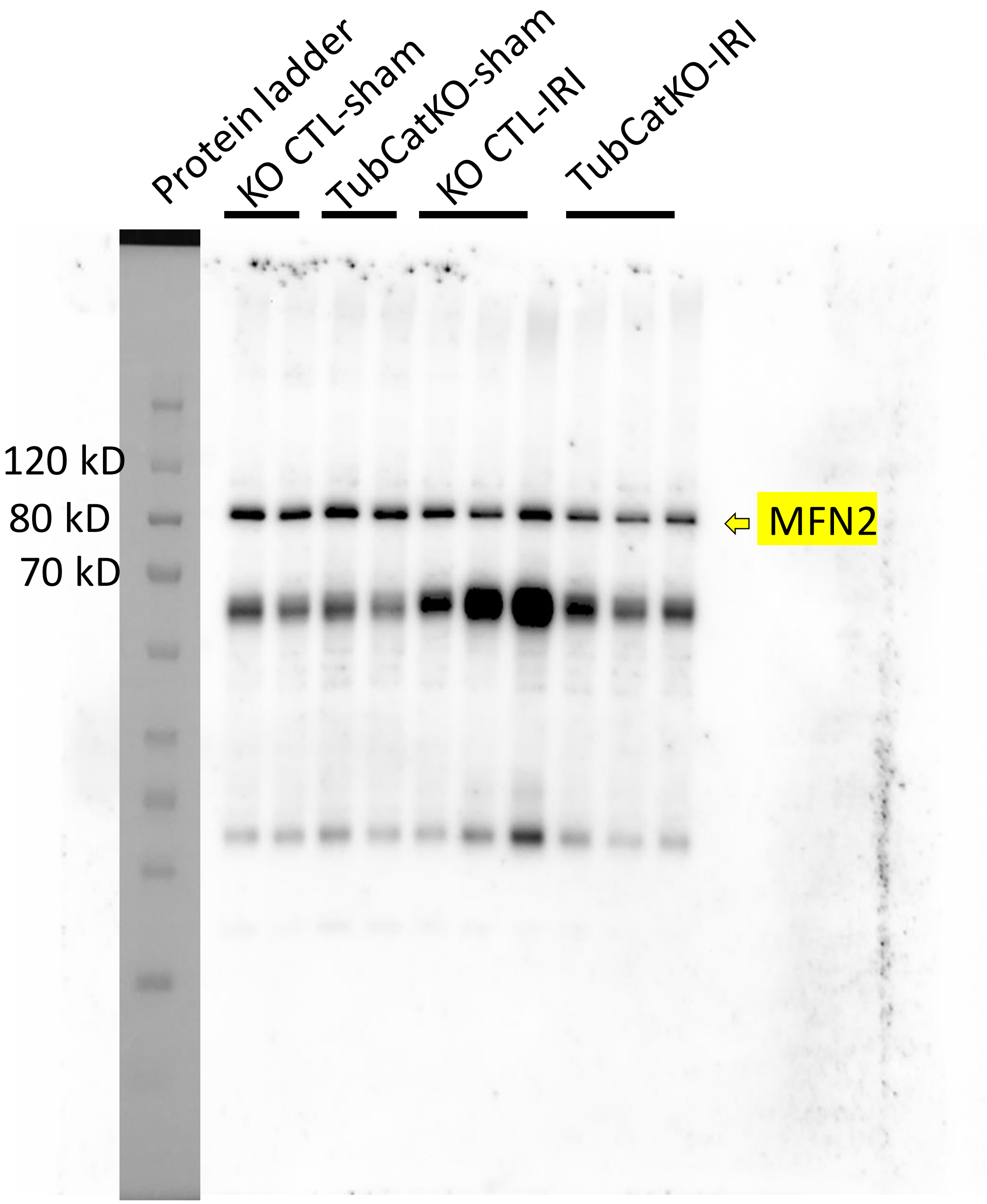

Review files– western blot

70. Fig 7A, β-ACTIN for MFN2

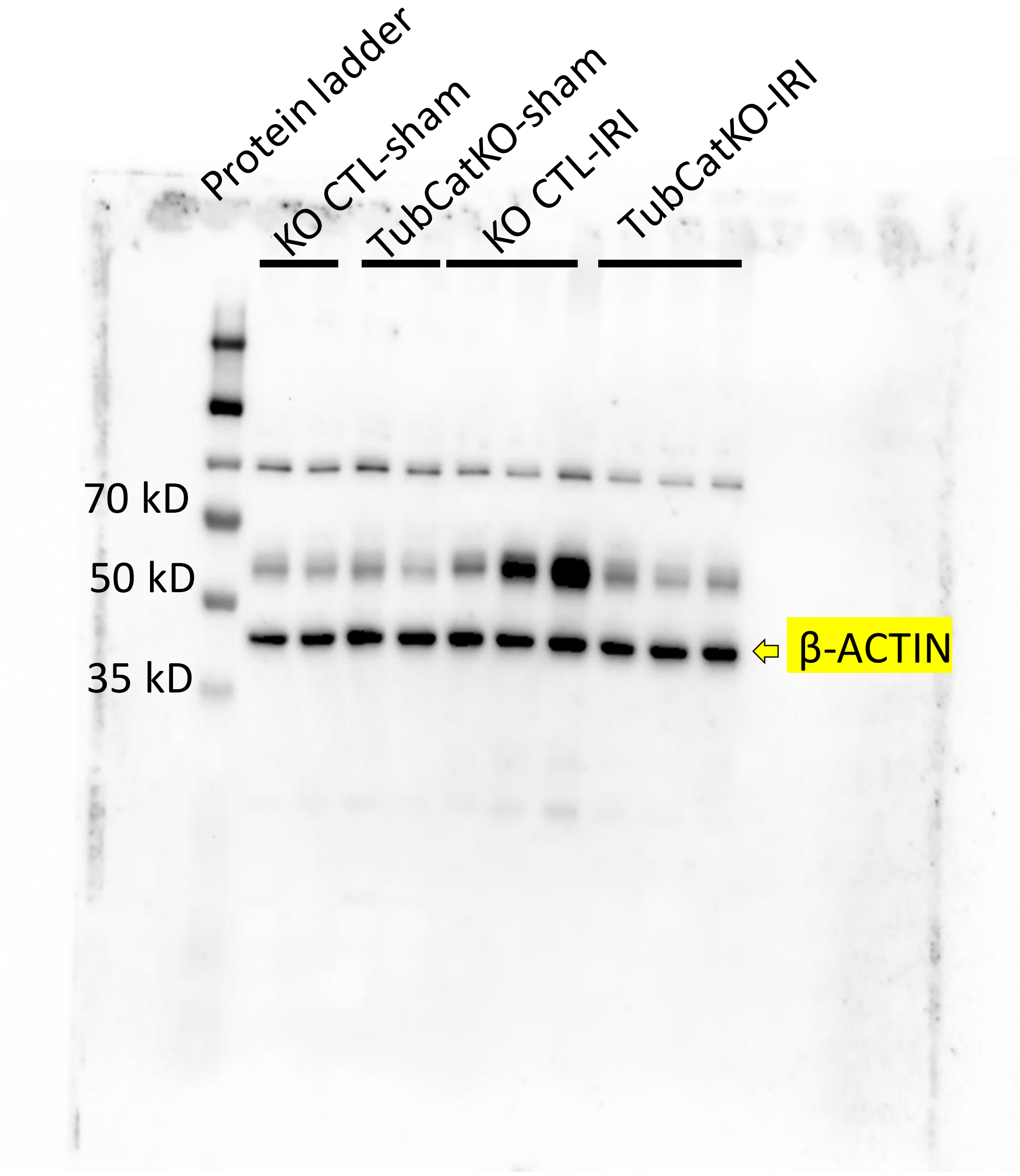

Review files– western blot

71. Fig 7B, DRP1

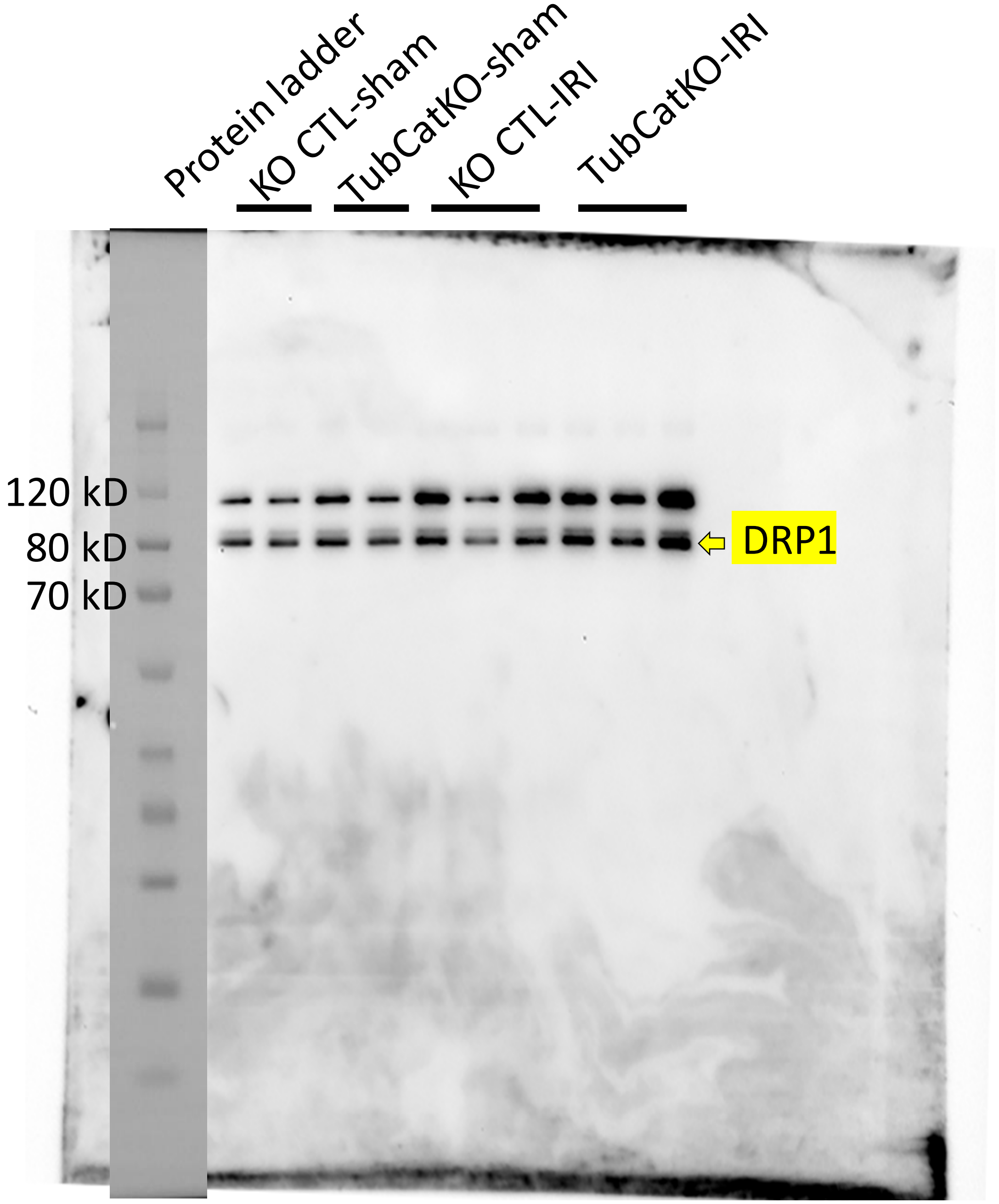

Review files– western blot

72. Fig 7B, β-ACTIN for DRP1, the representative β-ACTIN showed inFig 7B

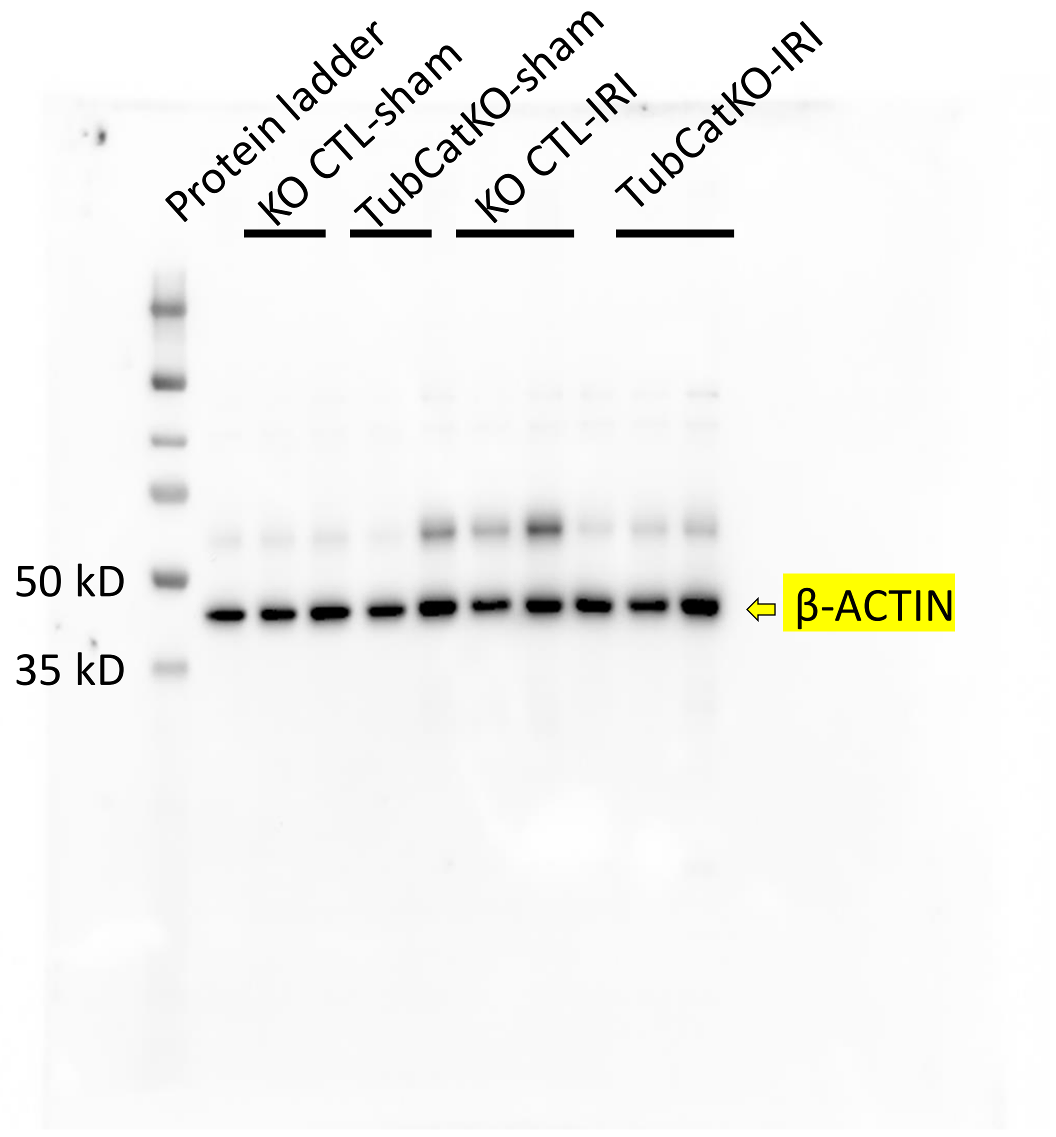

Review files– western blot

73. Fig 7C, OPA1

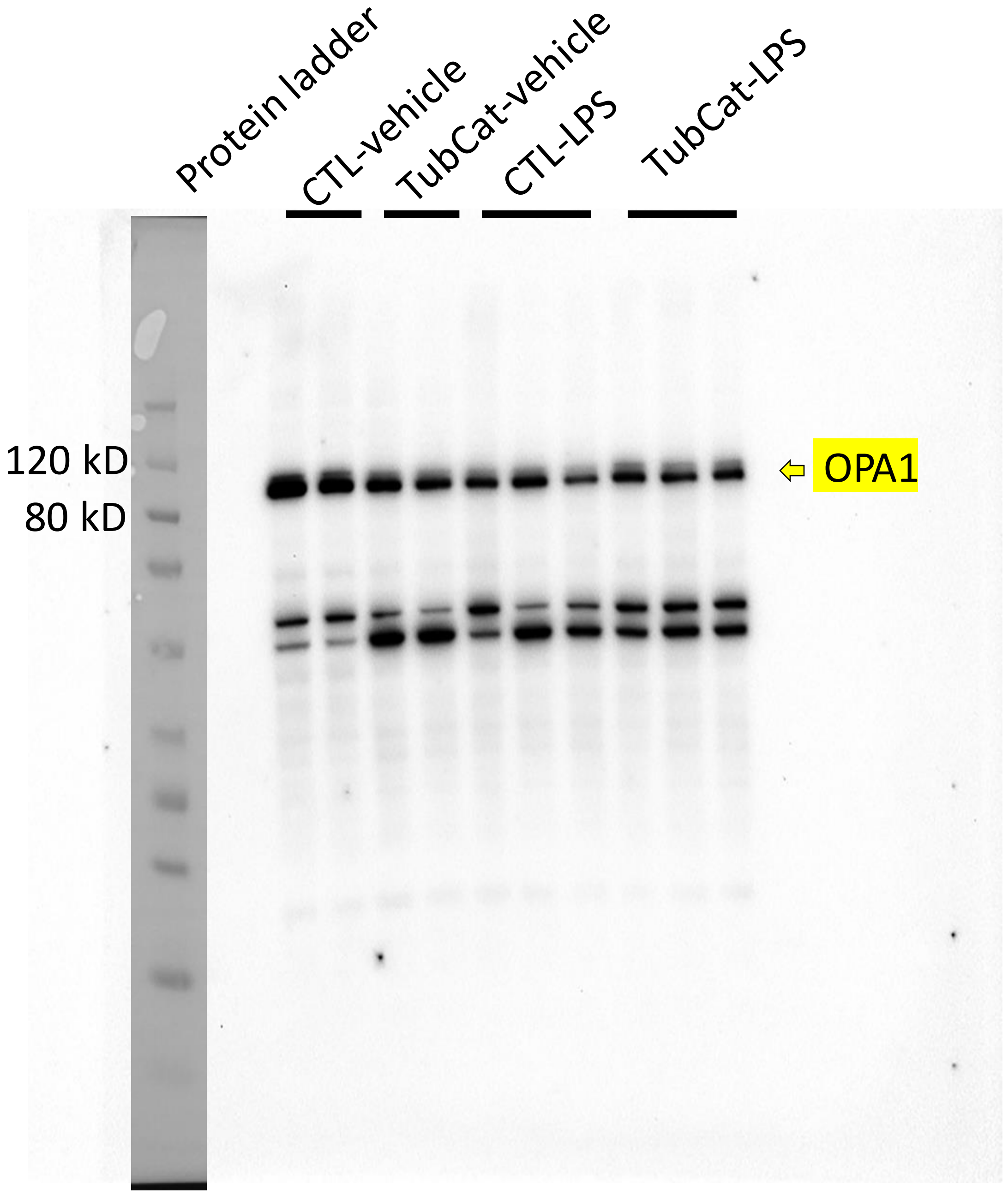

Review files– western blot

74. Fig 7C, β-ACTIN for OPA1, the representative β-ACTIN showed in Fig 7C

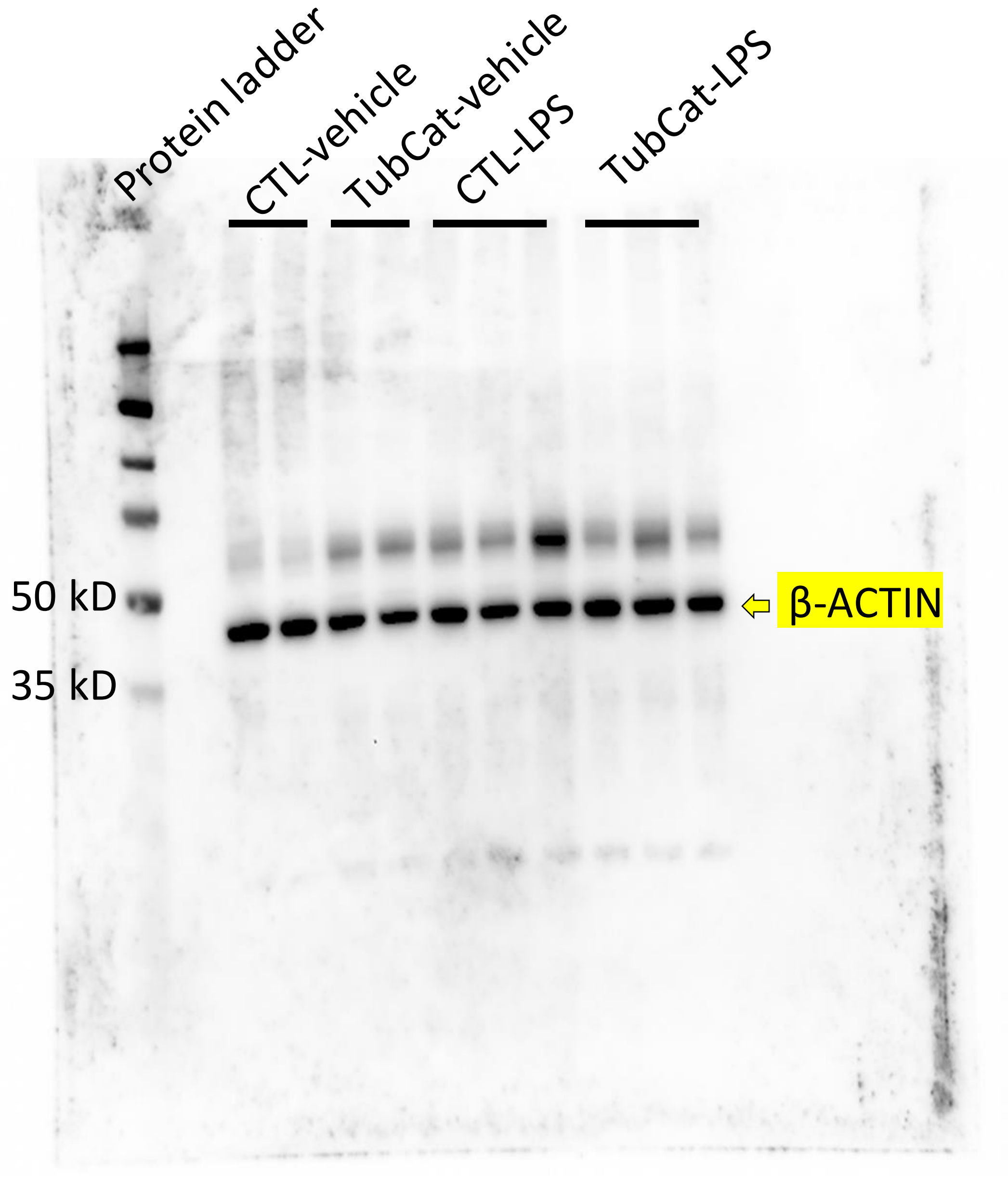

Review files– western blot

75. Fig 7C, MFN2

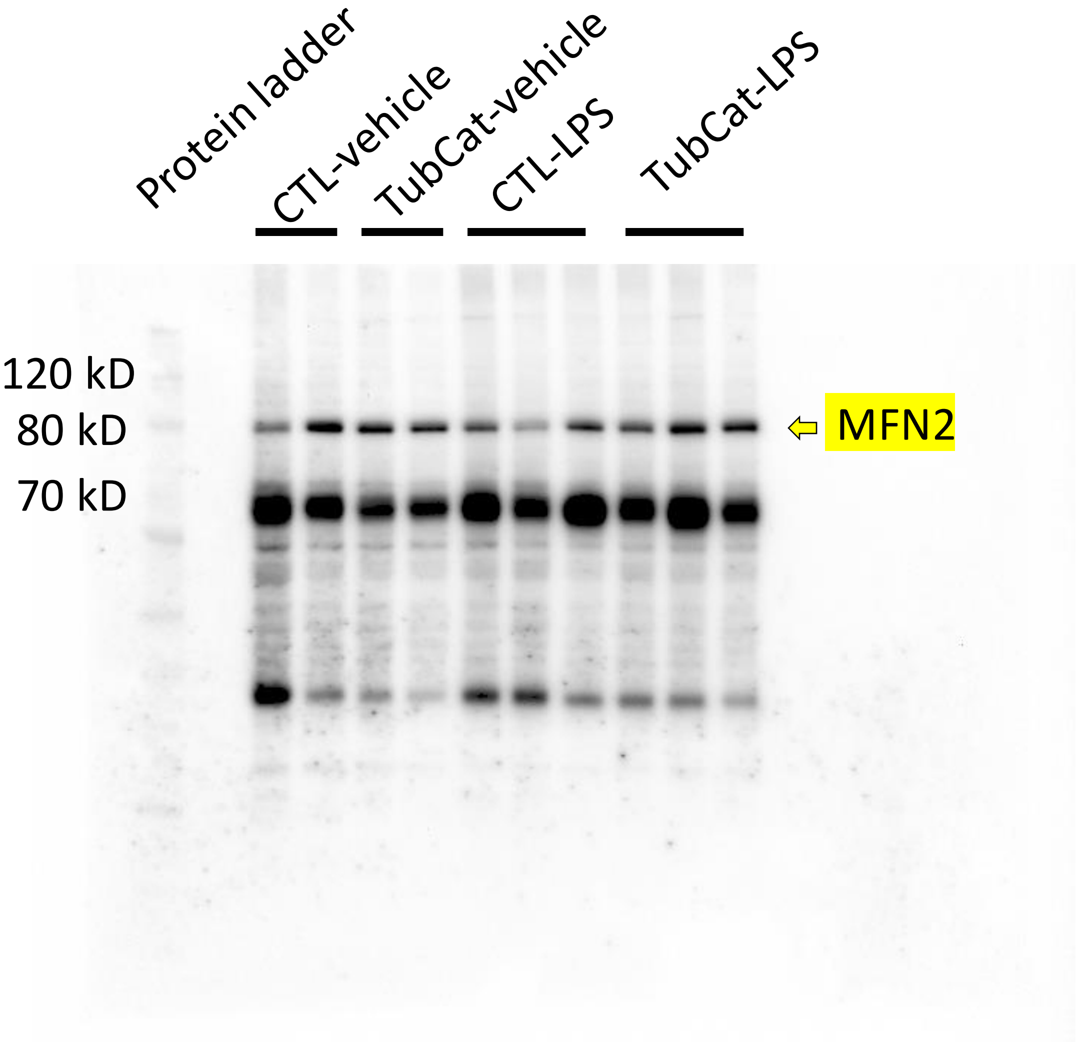

Review files– western blot

76. Fig 7C, β-ACTIN for MFN2

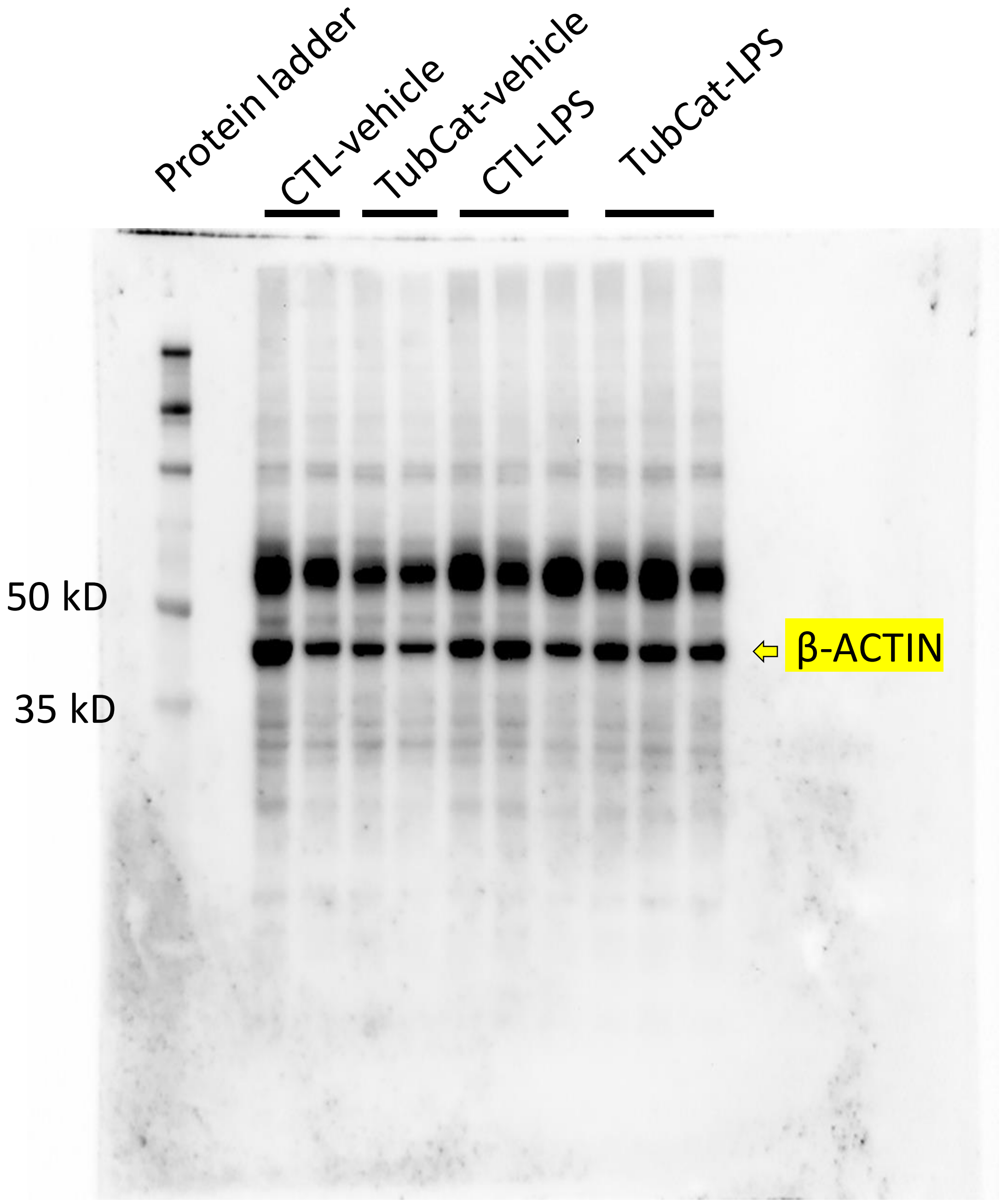

Review files– western blot

77. Fig 7C, DRP1

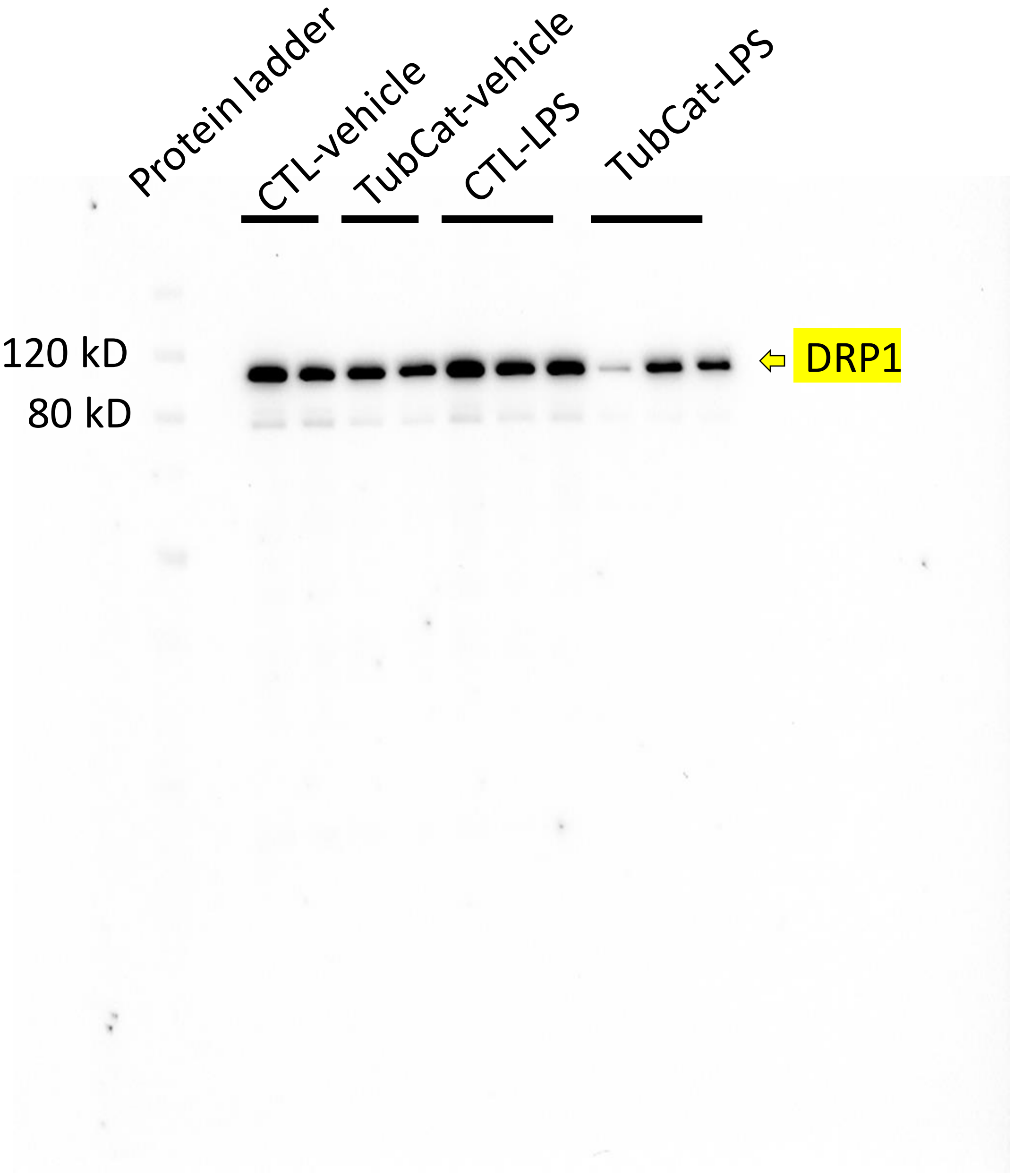

Review files– western blot

78. Fig 7C, β-ACTIN for DRP1

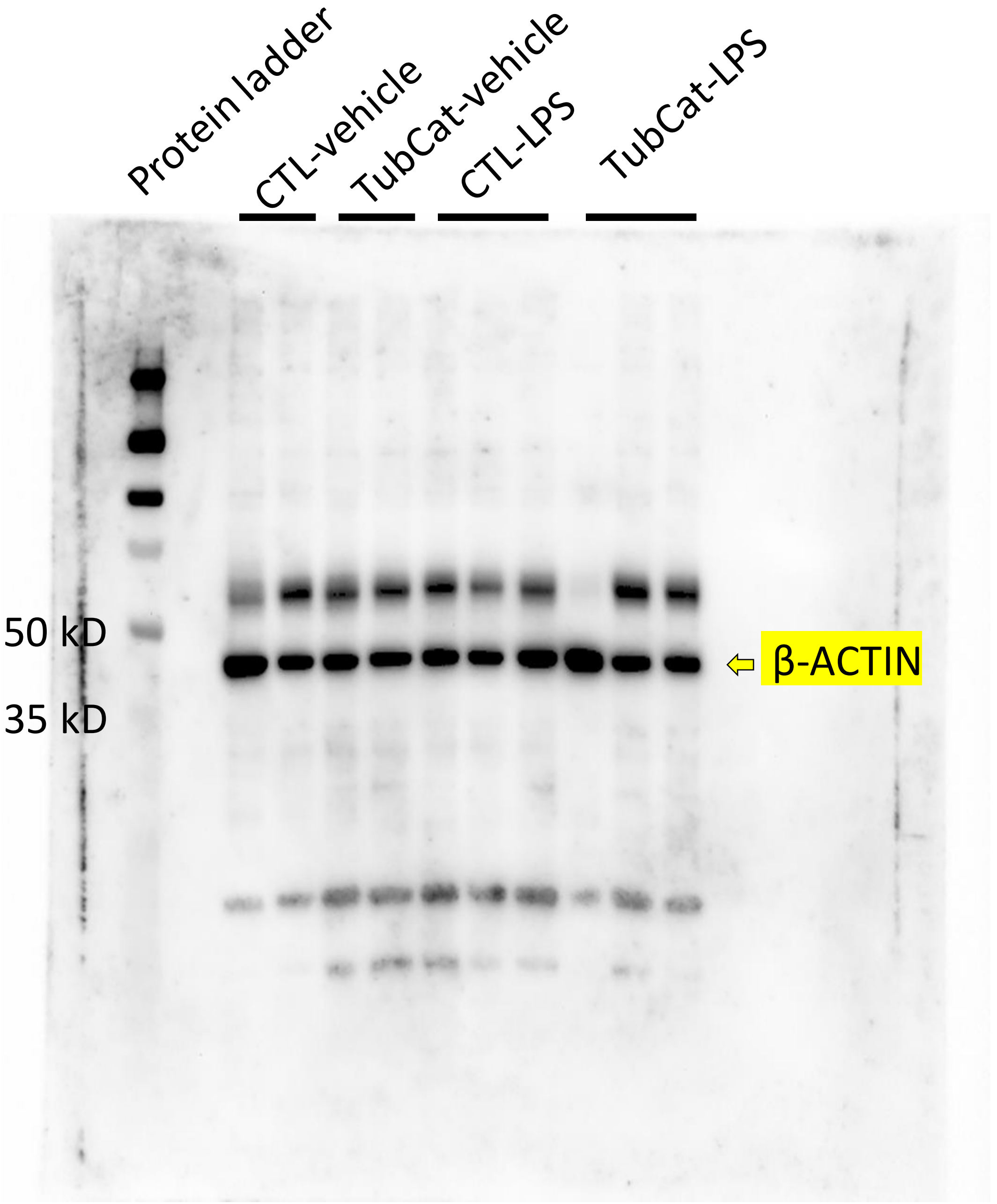

Review files– western blot

79. Fig 7D, OPA1

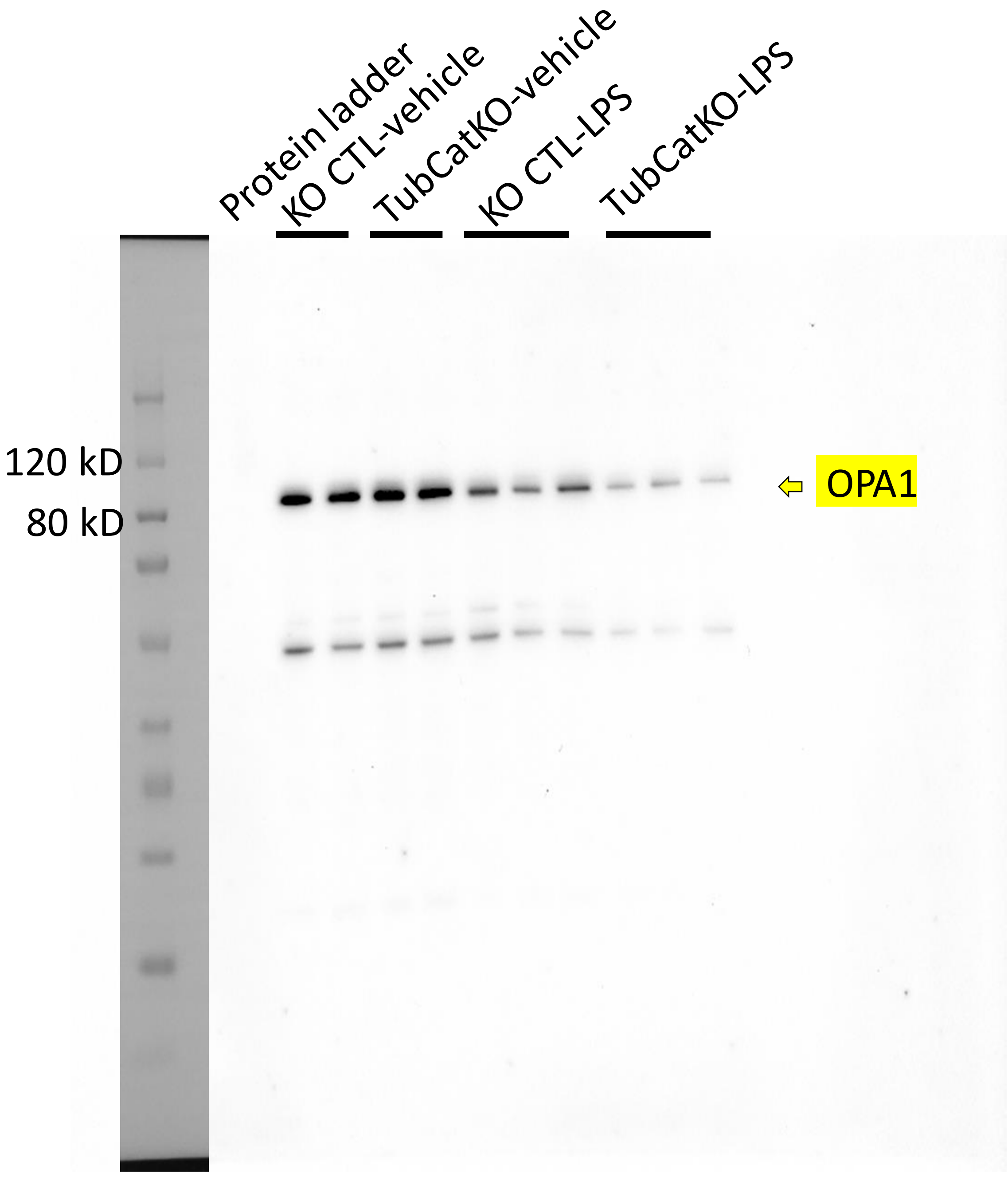

Review files– western blot

80. Fig 7D, β-ACTIN for OPA1

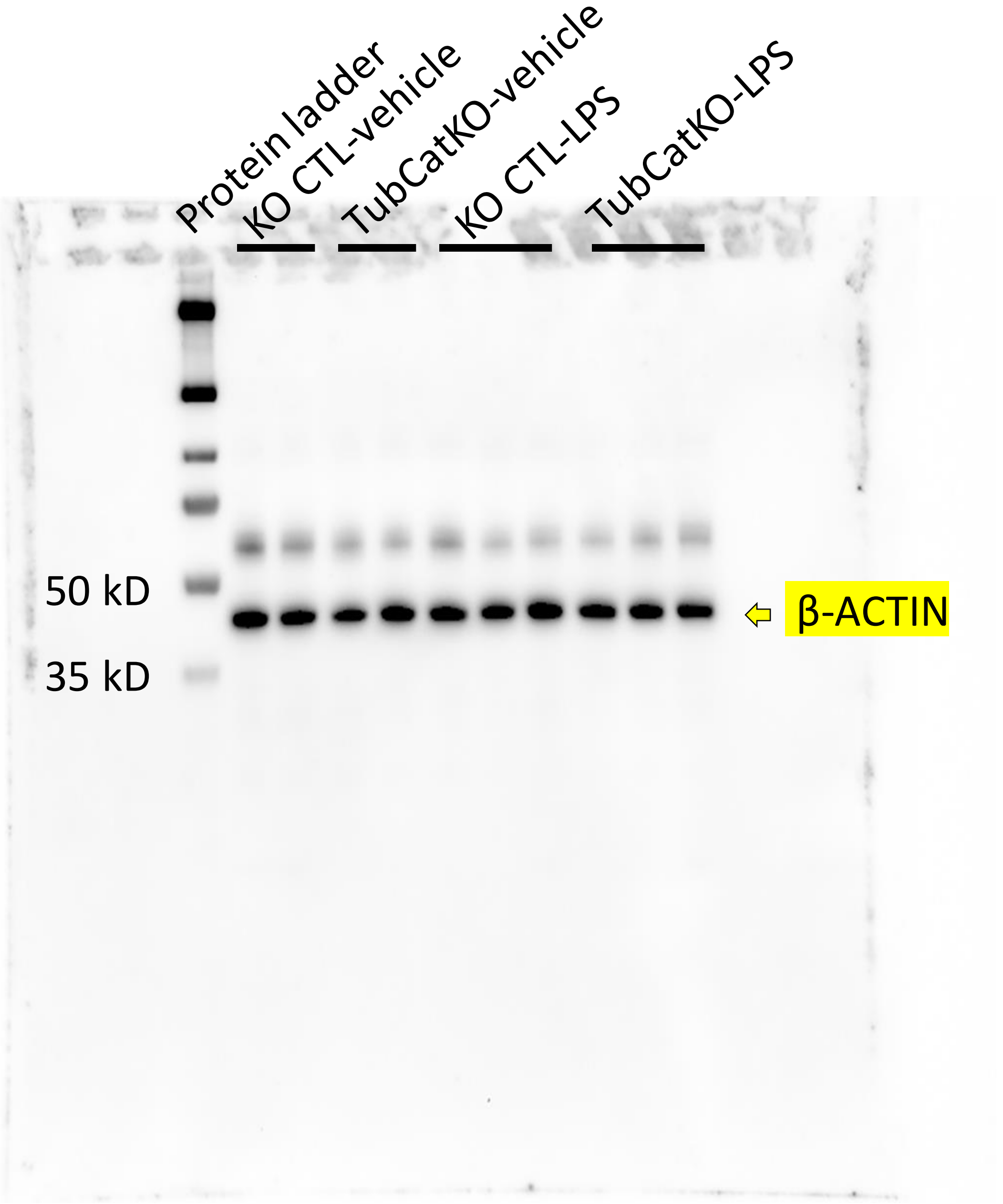

Review files– western blot

81. Fig 7D, MFN2

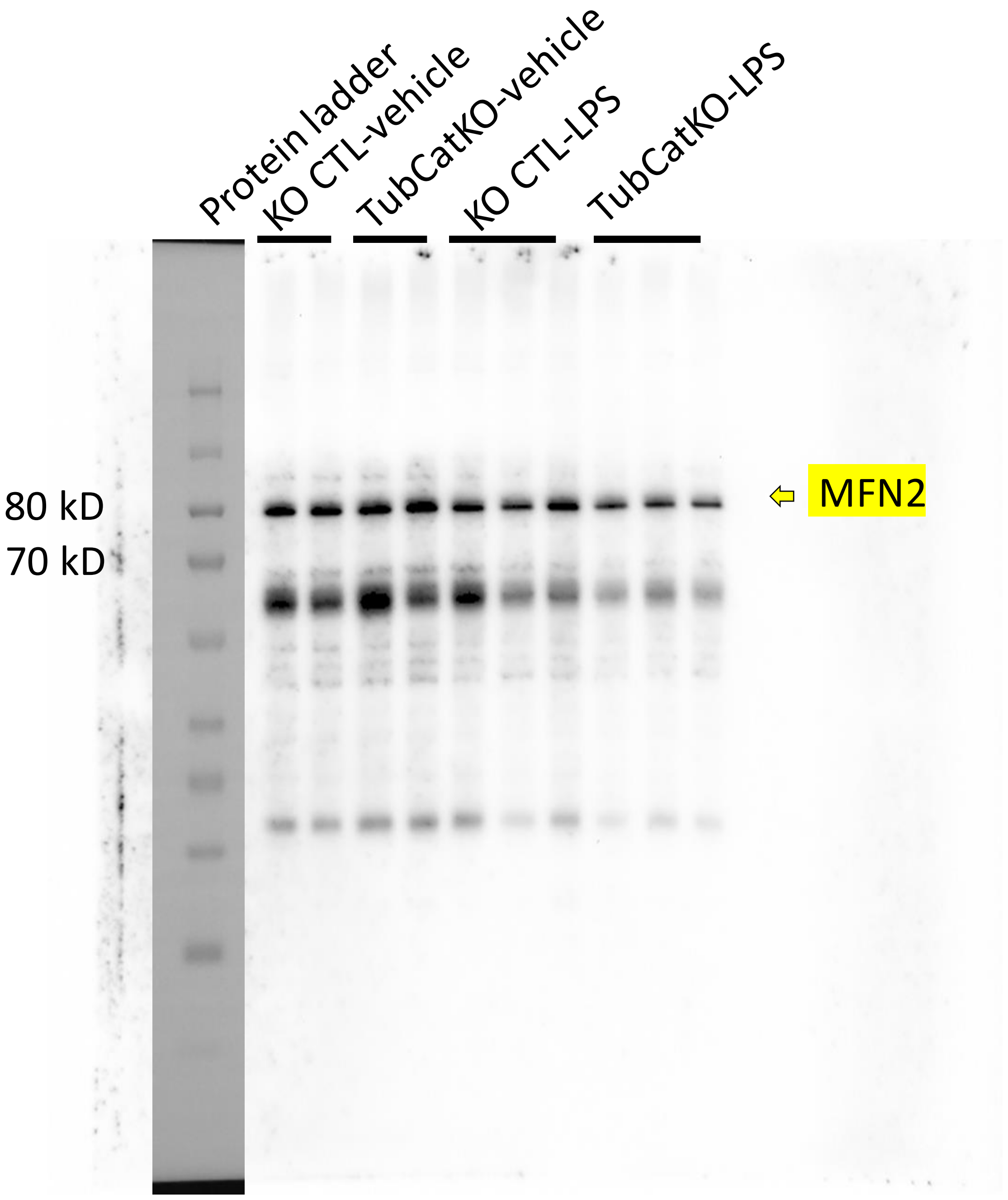

Review files– western blot

82. Fig 7D, β-ACTIN for MFN2

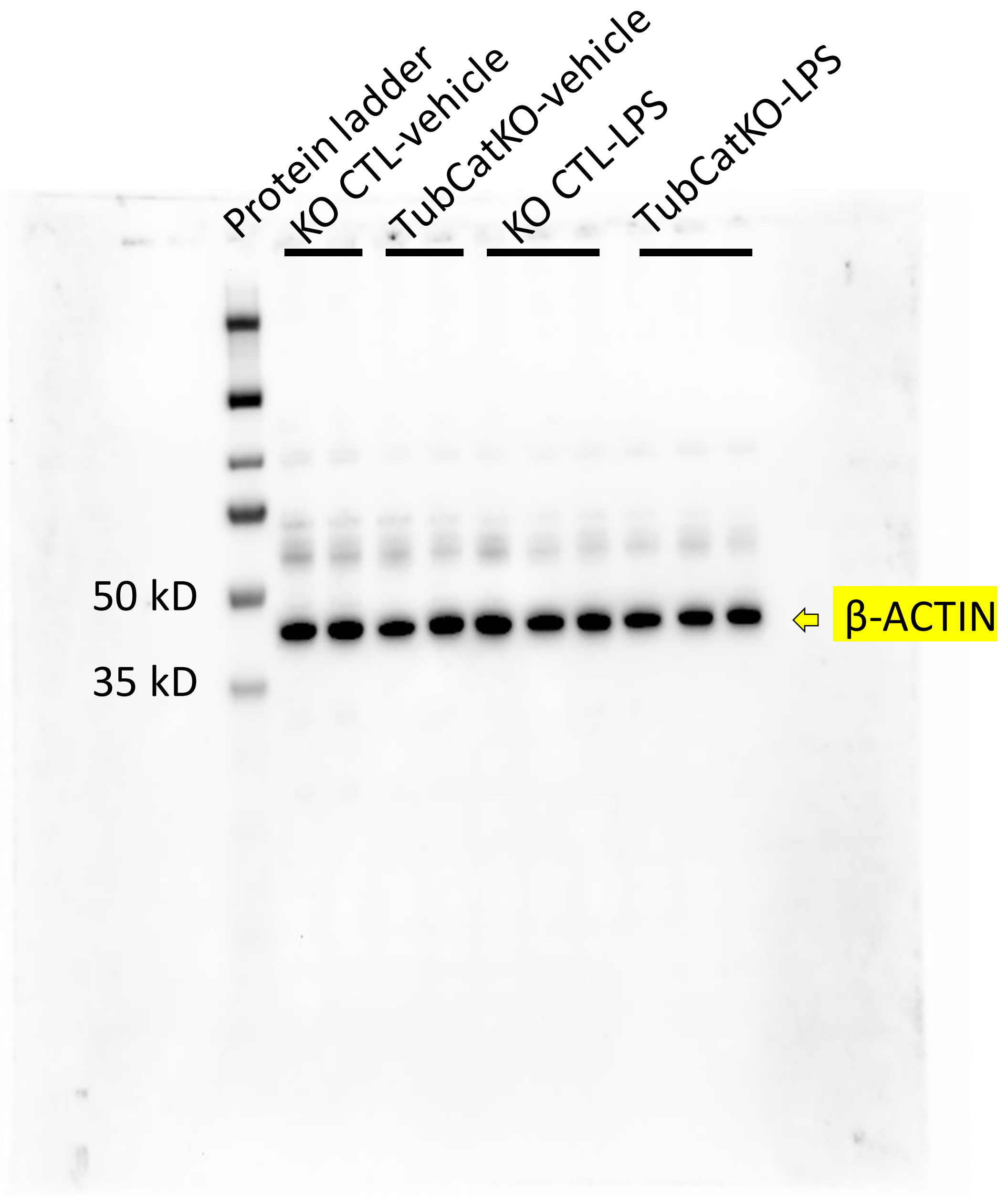

Review files– western blot

83. Fig 7D, DRP1

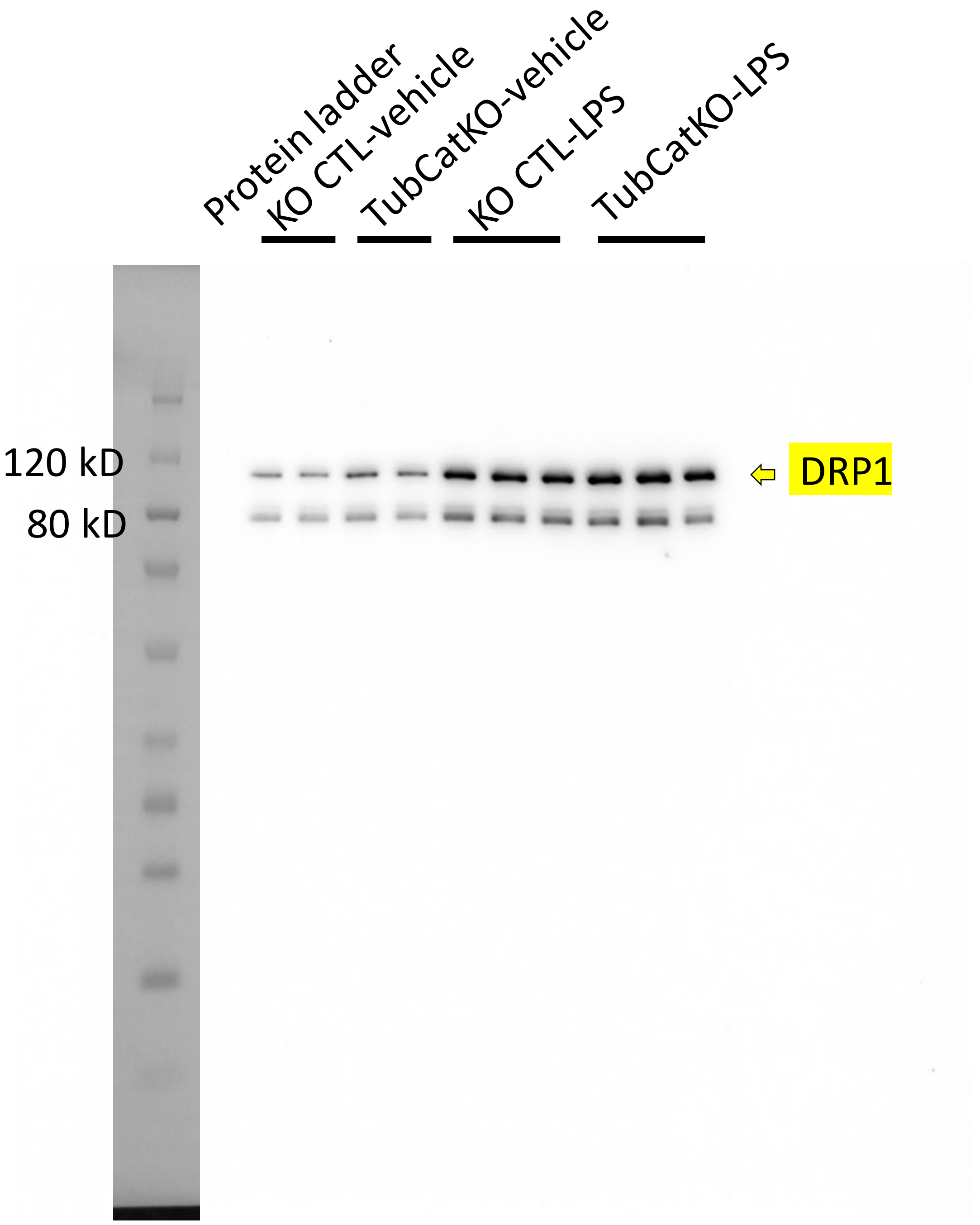

Review files– western blot

84. Fig 7D, β-ACTIN for DRP1, representative band showed in Fig 7D

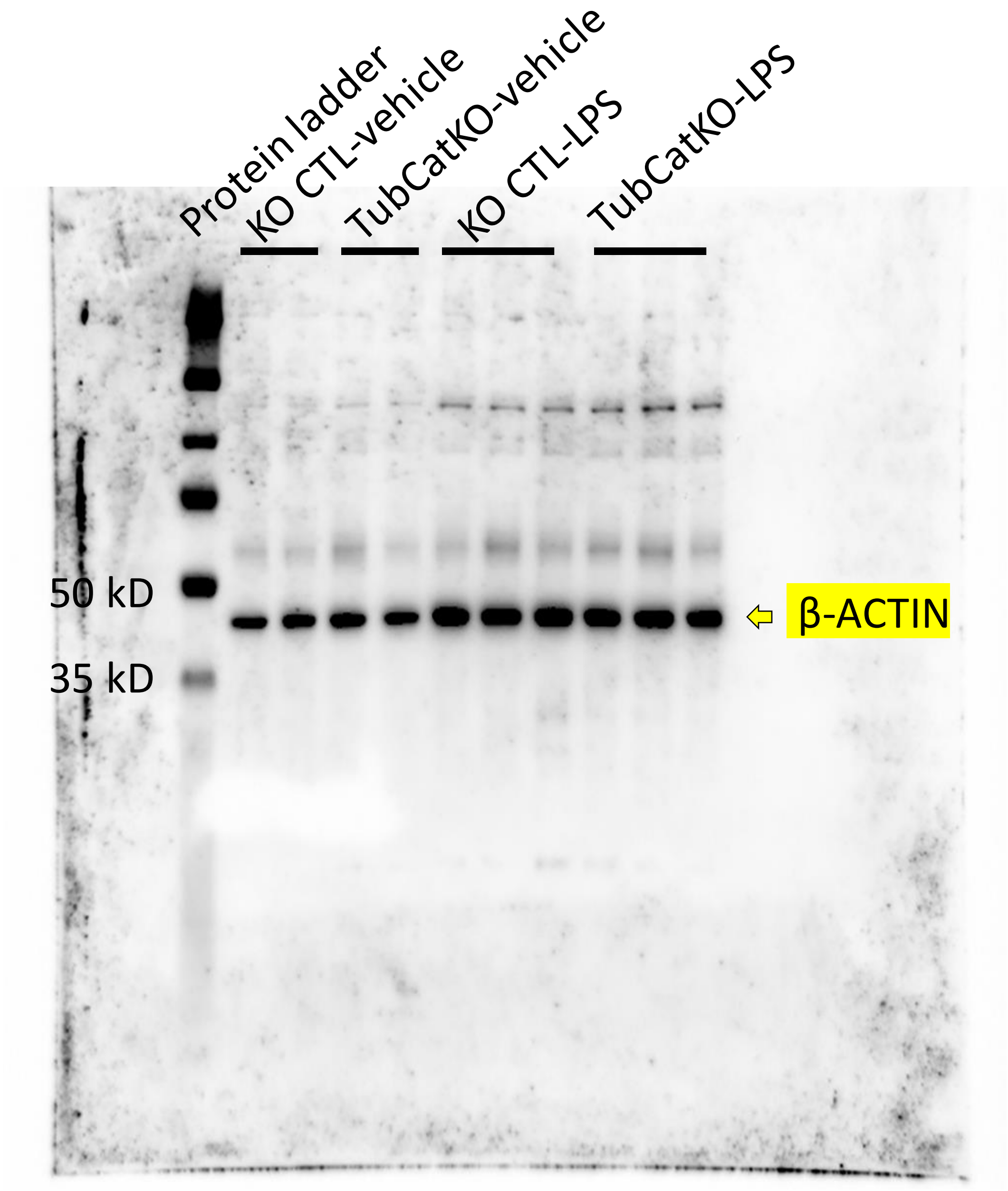

Review files– western blot

85. Fig 8A, PGC-1α

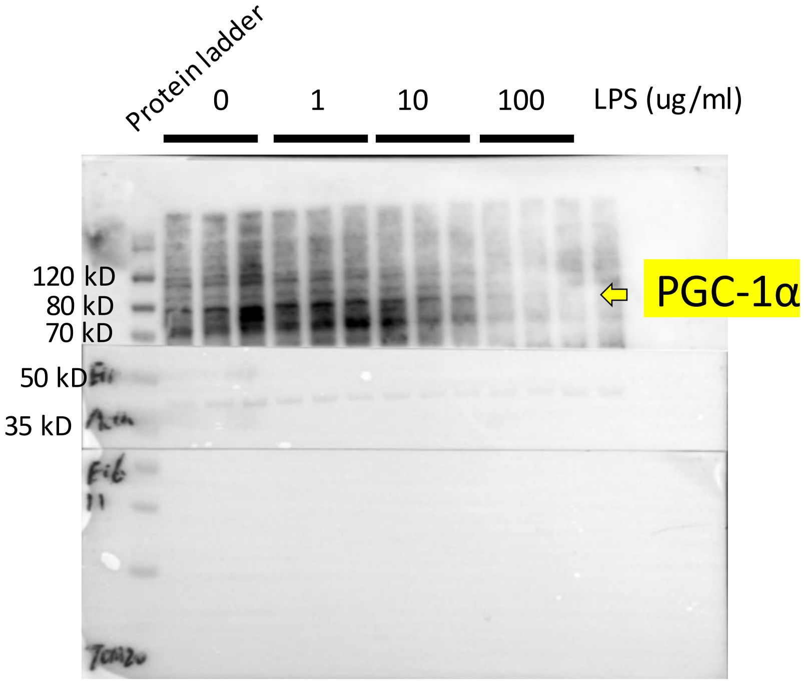

Review files– western blot

86. Fig 8A, β-ACTIN for PGC-1α and NRF1

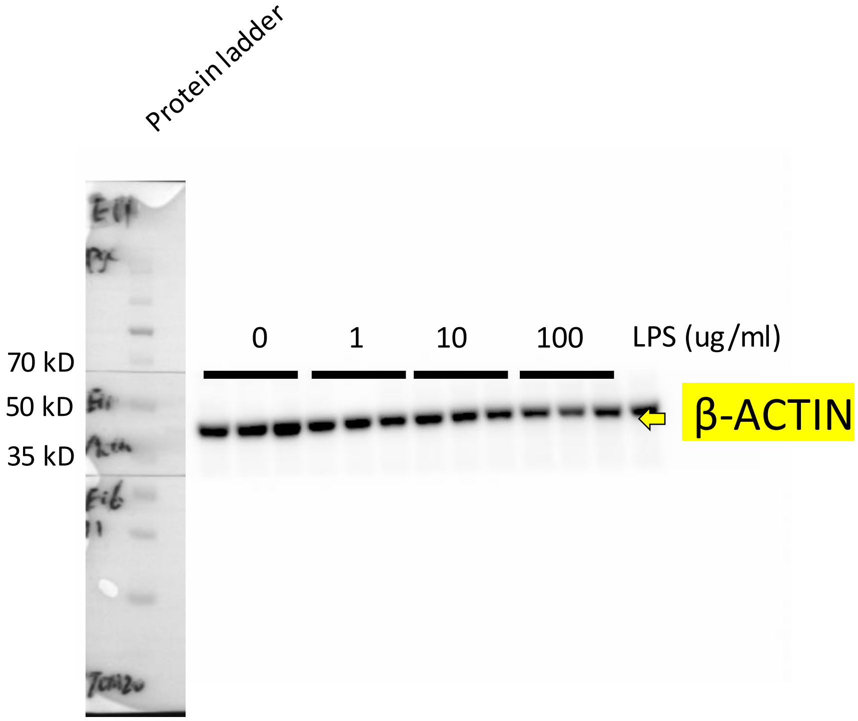

Review files– western blot

87. Fig 8A, NRF1

Note: NRF1 Gel loaded the same sample and volume as PGC-1α in Fig 8A so they shared the same loading control (β-ACTIN)

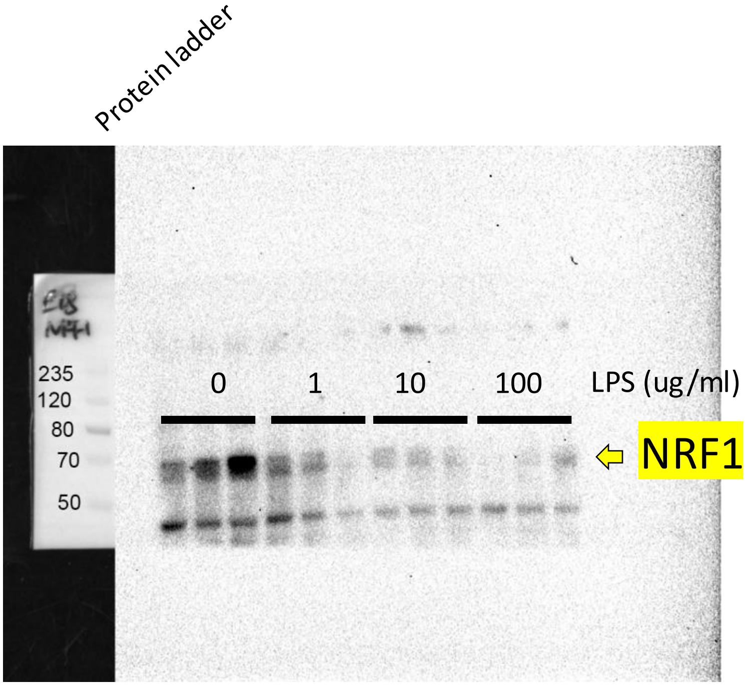

Review files– western blot

88. Fig 8B, PGC-1α

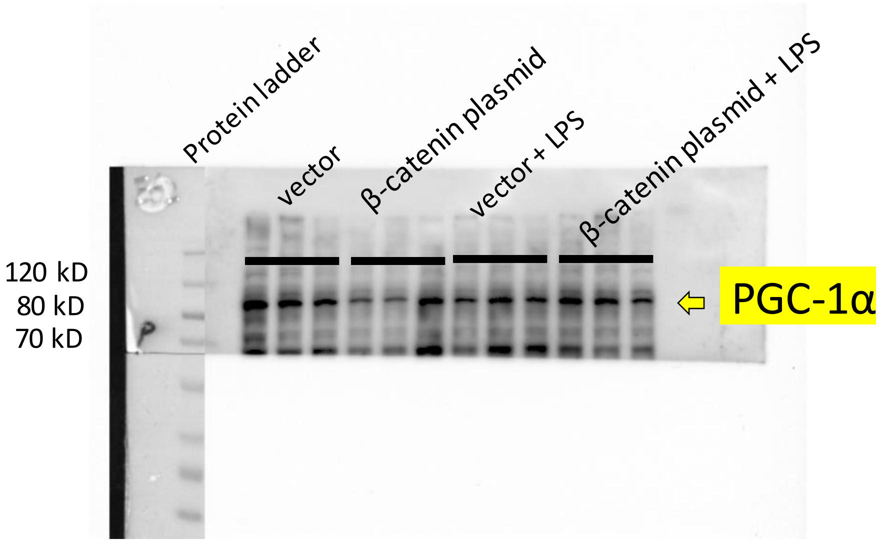

Review files– western blot

89. Fig 8B, β-ACTIN

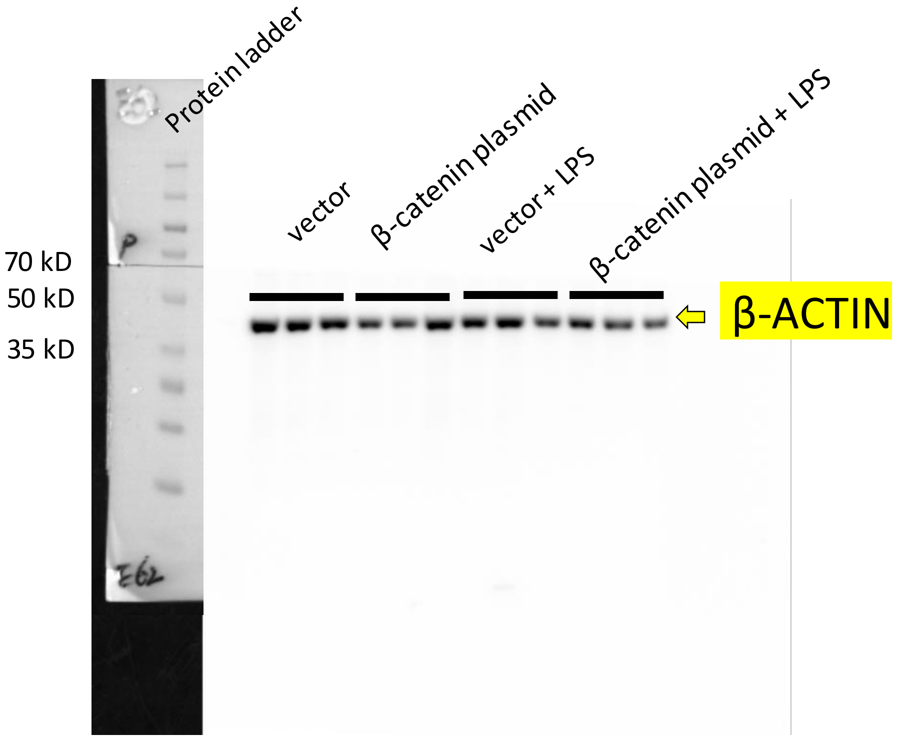

Review files– western blot

90. Fig 8D, FOXO3

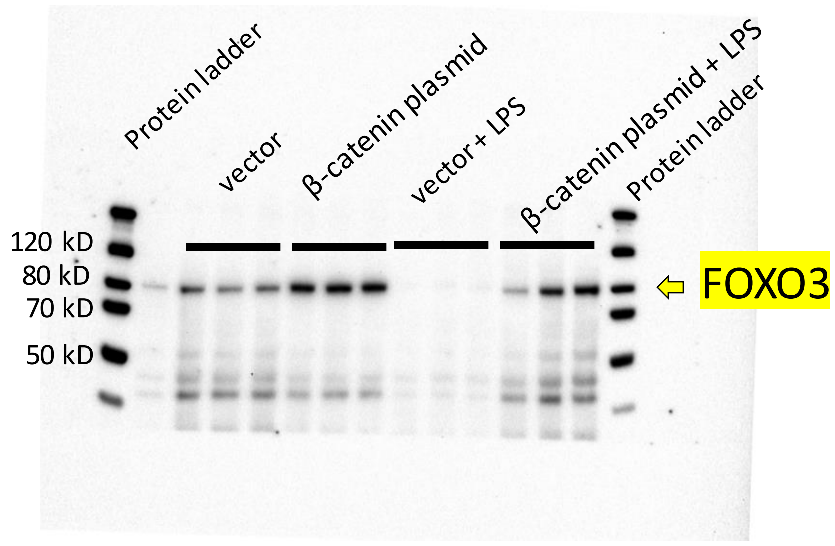

Review files– western blot

91. Fig 8D, HDAC1 for FOXO3

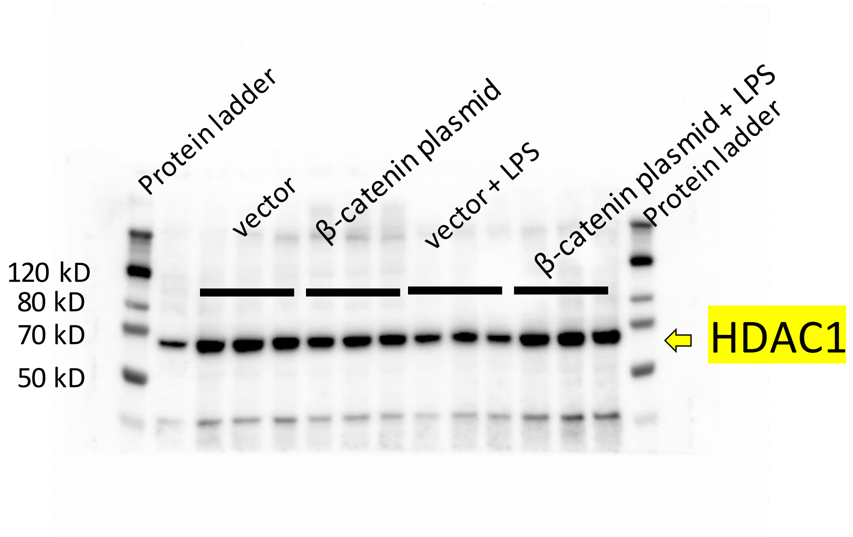

Review files– western blot

92. Fig 8F, β-catenin from untreated group

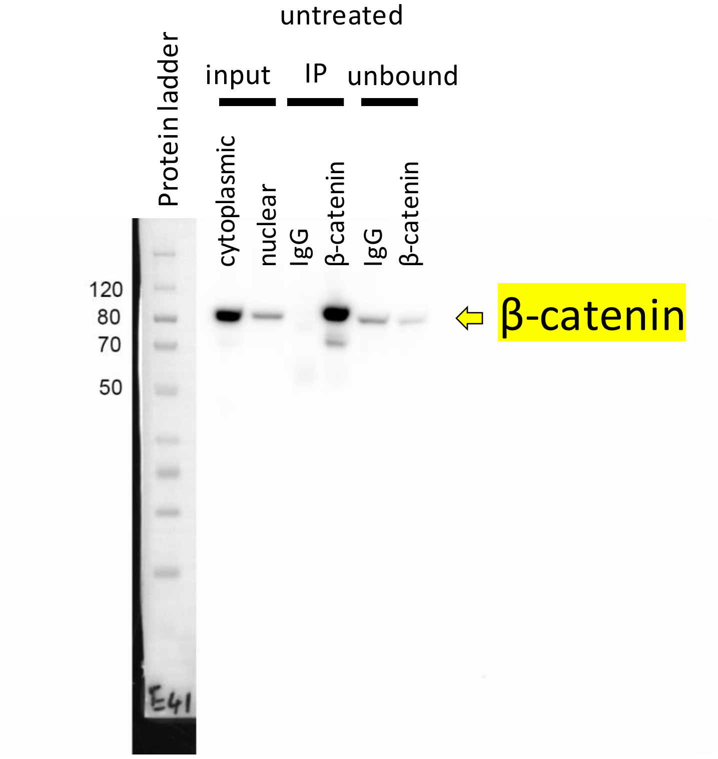

Review files– western blot

93. Fig 8F, FOXO3 from untreated group

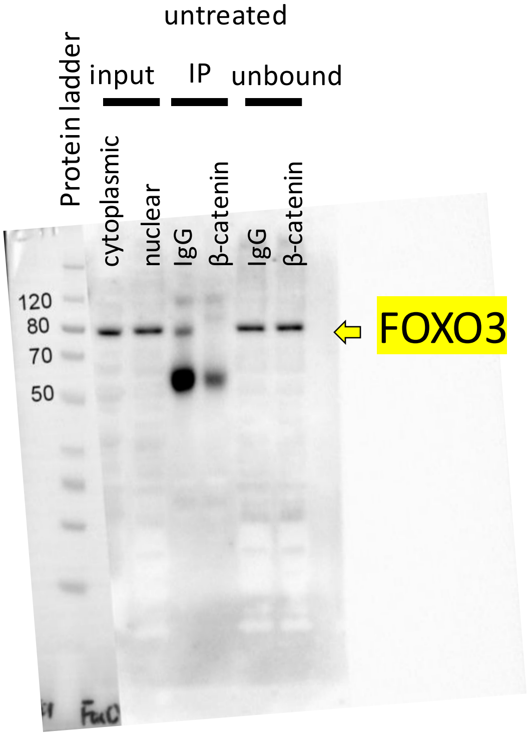

Review files– western blot

94. Fig 8F, GAPDH from untreated group

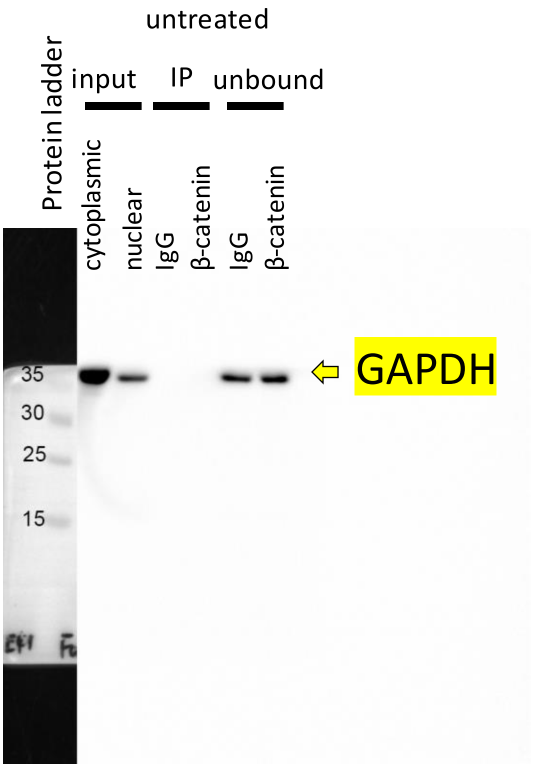

Review files– western blot

95. Fig 8F, β-catenin from LPS-treated group

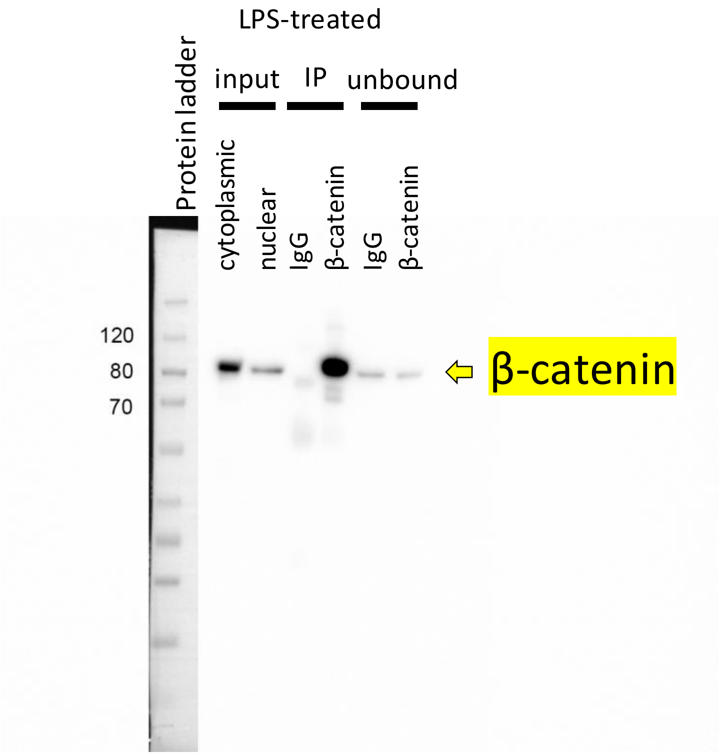

Review files– western blot

96. Fig 8F, FOXO3 from LPS-treated group

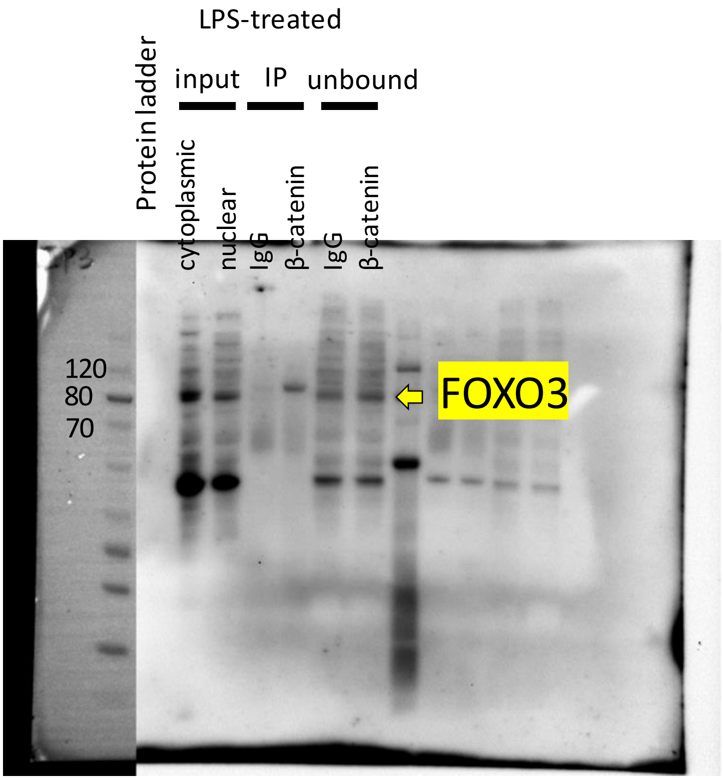

Review files– western blot

97. Fig 8F, GAPDH from LPS-treated group

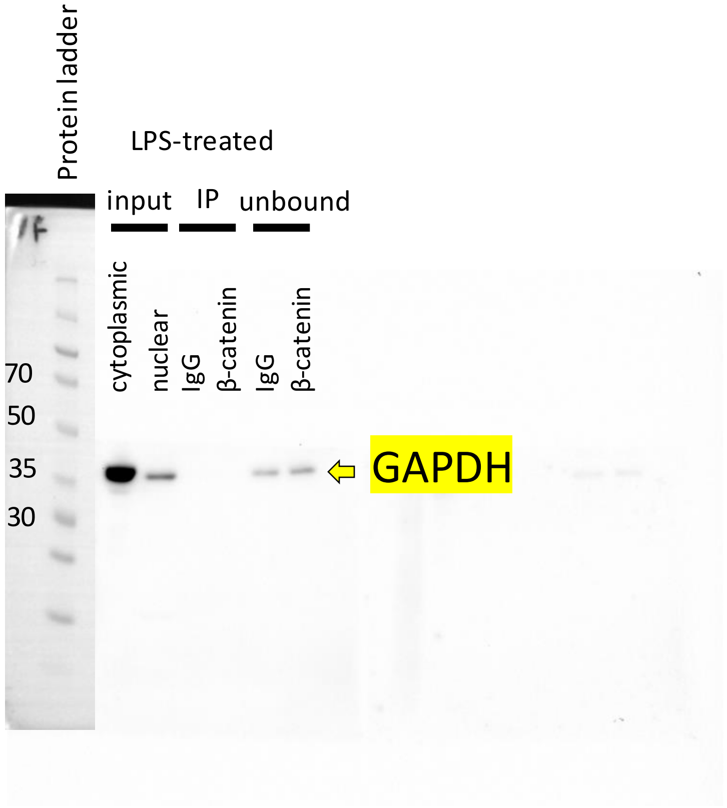

Supplement: Supplementary file 2 — Original Western blots [file 41419_2022_5395_MOESM2_ESM.pdf]
